# Ultrasound and Microwave-Assisted Synthesis and Antidiabetic and Hematopoietic Activity of Diphenhydramine Derivatives

**DOI:** 10.3390/molecules30142967

**Published:** 2025-07-15

**Authors:** Anuar Dauletbakov, Yelizaveta Belyankova, Saniya Assylbekova, Darya Zolotareva, Sarah Bayazit, Layilya Baktybayeva, Ulan Kemelbekov, Valentina Yu, Nailya Ibragimova, Alexey Zazybin

**Affiliations:** 1 School of Chemical Engineering, Kazakh-British Technical University, 59 Tole bi Str., Almaty 050000, Kazakhstan; dayletbakovanuar@gmail.com (A.D.); belyankovae@gmail.com (Y.B.); erzhanovnasss@gmail.com (S.A.); zolotareva.2909@mail.ru (D.Z.); bayazitsarah@gmail.com (S.B.); 2 Department of Biophysics, Biomedicine and Neuroscience, Al-Farabi Kazakh National University, al-Farabi Ave, 71, Almaty 050040, Kazakhstan; landau753@gmail.com; 3 Pharmacy Scientific Research Center, South Kazakhstan Medical Academy, 1 Al-Farabi Square, Shymkent 160019, Kazakhstan; kemelbekovulan@gmail.com; 4 JCS «A.B. Bekturov Institute of Chemical Sciences», Almaty 050010, Kazakhstan; yuvkconst@gmail.com; 5 Scientific Center for Anti-Infectious Drugs, Laboratory of Pharmacology and Toxicology, Auezov Str., 84, Almaty 050000, Kazakhstan; nailya.73@mail.ru

**Keywords:** antidiabetic activity, α-glucosidase inhibition, α-amylase inhibition, hematopoietic activity, microwave synthesis, ultrasonic synthesis, diphenhydramine

## Abstract

This study presents the synthesis and antidiabetic and hematopoietic activity of ionic compounds based on 2-(diphenylmethoxy)-*N*,*N*-dimethylethanamine (diphenhydramine). Synthesis is carried out under ultrasonic (US) and microwave (MW) irradiation as well as using a conventional method (thermal activation). The synthesized ionic compounds have been tested for antidiabetic effect according to the inhibitory action against α-glucosidase and α-amylase (in vitro). All the synthesized derivatives of diphenhydramine showed higher inhibitory activity against α-glucosidase than commercially available diphenhydramine hydrochloride. Moreover, two of them, **1m** (66.9%) and **1k** (64.2%), had a greater inhibitory activity than the reference drug acarbose (51.8%). The hematopoietic activity was studied in albino laboratory female rats (in vivo). The compounds **1b**, **1f**, and **1k** can restore immune blood cells (hematopoietic activity), equal to or exceeding that of the commercially available diphenhydramine hydrochloride and control (methyluracil).

## 1. Introduction

In recent years, great progress has been made in creating various designs of effective ultrasound (US) and microwave (MW)-promoted synthesis, and, therefore, there is an increased interest in the use of ultrasound and microwave activation to intensify various chemical reactions [1,2]. Ultrasound and microwave irradiation have a significant impact on the reaction rate and the direction of the process [3]. Many reactions carried out using ultrasound and microwave activation do not proceed in their absence or cannot be used for preparative purposes. In some cases, the use of ultrasound and microwave irradiation can increase the selectivity of chemical processes [4]. Novel synthesis methods that require less energy and time are playing an important role in green chemistry. The use of alternative synthesis methods, such as US and MW, becomes more efficient by reducing the synthesis time, raising the yield, and increasing the purity of the product [5,6]. Furthermore, due to milder reaction conditions, fewer or suppressed side reactions, energy-saving quality, and the use of moderate amounts of solvents, the reactions become environmentally friendly. In this regard, conducting research aimed at studying the possibility of widespread use of microwave radiation and ultrasound to intensify the reactions underlying the synthesis of valuable organic compounds is an urgent task.

Currently, both types of diabetes mellitus (DM type 1 and DM type 2) pose serious health problems worldwide due to their prevalence and the significant macro- and microvascular complications that contribute to increased disability and mortality rates in the population. According to the International Diabetes Federation, there were 382 million people suffering from diabetes worldwide in 2013, and according to forecasts, the number of such patients may increase to 592 million by 2035, i.e., by 55%. At the same time, type 2 diabetes is diagnosed in 85–95% of all diabetes cases [7]. Modern medicines have a significant number of antidiabetic drugs that affect various links in the pathogenesis of this disease—stimulating the secretion of endogenous insulin, increasing the sensitivity of peripheral tissues to insulin (sensitizers), and slowing the absorption of glucose in the intestine. Due to the appearance of new information about the pathogenetic mechanisms of the development of type 2 diabetes, incretin mimics appeared, whose use significantly expanded the possibilities for effective and safe treatment of this form of diabetes [8,9,10,11]. Considering the steadily increasing number of patients with type 2 diabetes, an urgent problem of modern pharmacology continues. There remains the need for the development of antidiabetic preparations based on new principles of action, possessing high therapeutic activity, and having an improved safety profile [12,13]. These may be new medicinal substances of synthetic or herbal origin, as well as drugs that have long been used in medical practice, which can prevent the further progression of diabetes and the development of its complications.

Up to 18% of cancer patients have diabetes, according to [14]. Diabetes has been linked to a 25–41% increased risk of mortality from any form of cancer, according to [15,16]. In this regard, the pursuit of novel hematopoietic drugs that also exhibit anti-diabetic properties is pertinent.

The most important stage in the creation of new medicinal substances or the study of new properties of known drugs is their preclinical investigation. In this regard, it is of great importance to use experimental models of DM, allowing us to reliably detect the presence of a particular pharmacological activity or new details in the mechanism of action of drugs.

Diphenhydramine, the core compound for the synthesis of new derivatives in the current study, has a wide range of biological activity, including local anesthetic [17] and antihistamine properties. The previous investigations show that antihistamines might cause genotoxicity; also, diphenhydramine has an ecotoxic activity towards bacteria *A. fischeri* [18,19]. So, the search for the new diphenhydramine derivatives, expanding the typical range of bioactivity of diphenhydramine, is of great relevance to medicinal chemistry.

In this article, we report on the synthesis of ionic compounds based on diphenhydramine under US and MW irradiation compared with the conventional method (thermal heating). The synthesized ionic compounds were found to possess strong antidiabetic activity according to the degree of inhibition of α-glucosidase and α-amylase, as well as hematopoietic activity.

## 2. Results and Discussion

### 2.1. Synthesis of Diphenhydramine Derivatives

**Figure 1:**
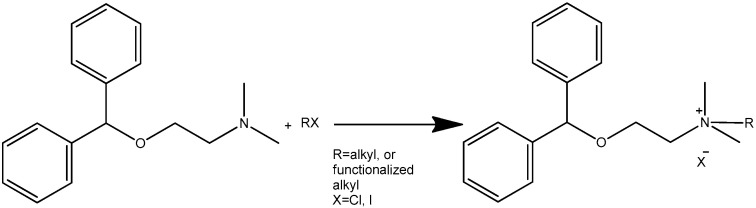
Synthesis of diphenhydramine derivatives (**1a**–**1m**).

The core compound 2-(diphenylmethoxy)-*N*,*N*-dimethylethanamine (diphenhydramine base) was obtained from commercially available hydrochloride by neutralization. The general equation for *N*-alkylation of diphenhydramine with alkyl halides is given in Figure 1.

Synthesized ionic compounds based on diphenhydramine, their names and structural formulas can be found in Supplementary Materials in Table S1. The results of the *N*-alkylation reaction were collected and are shown in Table 1.

The results from Table 1 show that the use of MW activation in the *N*-alkylation of diphenhydramine has the advantages of increasing the rate of reaction, dramatically reducing the reaction time, saving energy, and increasing the yields. The effectiveness of US activation in terms of lower reaction temperature and shorter reaction time can be explained by cavitation phenomena, increasing the mass transfer and homogenization of the reaction mixture. Most of the chemical reactions accelerated in a microwave oven are due to thermal effects caused by the extremely high rate at which heat can be introduced into a bulk medium using microwaves. Faster heating to high temperatures has even been shown to affect product selectivity in reactions. Other related thermal processes postulated for homogeneous systems are solvent superheating, nucleation-limited boiling, hot spot formation, selective heating of certain reactants in solution, and the elimination of the so-called near-wall effects occurring during convective heating.

### 2.2. Antidiabetic Activity

#### 2.2.1. Inhibitory Activity Against the Enzyme α-Glucosidase

Bioactivity of diphenhydramine, a potent antihistamine drug, was recently expanded to antidiabetic activity [20]. The antidiabetic activity of the synthesized derivatives of diphenhydramine was studied based on the degree of inhibition of α-glucosidase. The study of the degree of inhibition of α-glucosidase activity by the tested compounds was performed using a standard method with minor modifications. Acarbose, a well-known drug with α-glucosidase inhibitory activity (but also with deleterious side effects), was used as a reference drug at a concentration of 15 mM (positive control), and in parallel, a negative control was set without the addition of test compounds [21]. Inhibitory activity was expressed as a percentage (%) by the degree of inhibition of α-glucosidase in comparison with the negative control, which was calculated by Formula (1):

Inhibitory activity (%) = (1 − As/Ac) · 100%,(1)

where As is the optical density of the test compound. Ac is the optical density of the control.

The results of the study of the inhibitory activity of the tested compounds towards the enzyme α-glucosidase are shown in Table 2.

Strong inhibitory activity against α-glucosidase, exceeding the inhibitory activity of acarbose, was observed in **1m** (66.9%) and **1k** (64.2%).

Strong inhibitory activity against α-glucosidase, comparable to the inhibitory activity of acarbose, was observed in the following compounds: **1i** (50.2%), **1e** (49.0%), and **1f** (49.8%).

Moderate inhibitory activity against α-glucosidase was observed in the following compounds: **1c** (28.9%), **1d** (35.7%), **1g** (38.9%), and **1h** (27.8%).

Weak inhibitory activity against α-glucosidase was observed in the following compounds: **1a** (13.4%), **1b** (12.0%), and **1l** (20.7%).

Inhibitory activity against α-glucosidase was absent in the compounds 1·HCl and **1j**.

The comparison drug acarbose showed standard inhibitory activity against α-glucosidase, which was 51.8%.

Overall, two compounds (**1m** and **1k**) showed strong inhibitory activity against α-glucosidase, exceeding the inhibitory activity of the reference compound (acarbose). Compounds **1i**, **1e**, and **1f** showed inhibitory activity against α-glucosidase comparable to the inhibitory activity of acarbose.

#### Molecular Docking Study Between α-Glucosidase and Diphenhydramine Derivatives

A common computer simulation method for examining the binding process between macromolecules (such as enzymes) and small molecules is molecular docking. It uses the concepts of energy and structure/geometry matching to identify the optimal mode of binding and can be adjusted based on the conformation of the molecules (bond length, bond angle, dihedral angle, and other characteristics) [22]. Electrostatic, hydrogen bonding, hydrophobic, and van der Waals forces are the primary forces that determine the binding between macromolecules and small molecules.

The binding mechanism between α-glucosidase and diphenhydramine derivatives was clarified by the use of molecular docking. As it is seen from Table 3, most of the *N*-substituted diphenhydramine derivatives with short alkyl chains or polar groups (-CN, -OH, -COOH) show low or moderate inhibitory activity against α-glucosidase, which can be attributed to the weak hydrophobic interactions or the repulsion between polar groups of diphenhydramine derivatives and aromatic rings of tryptophan and phenylalanine inside the hydrophobic pocket within α-glucosidase. The best inhibitory behavior of *N*-substituted alkylaromatic diphenhydramine derivatives can be explained in terms of effective π–π stacking interactions between *N*-alkyl aromatic groups of diphenhydramine derivatives and aromatic rings inside the hydrophobic pocket within α-glucosidase. However, high inhibitory activity of **1k** (R = CH_2_C_6_H_5_) and **1m** (R = (CH_2_)_3_C_6_H_5_), exceeding inhibitory activity of reference drug acarbose, comes in contrast with negligible inhibitory activity of **1l** (R = (CH_2_)_2_C_6_H_5_).

**Figure 2:**
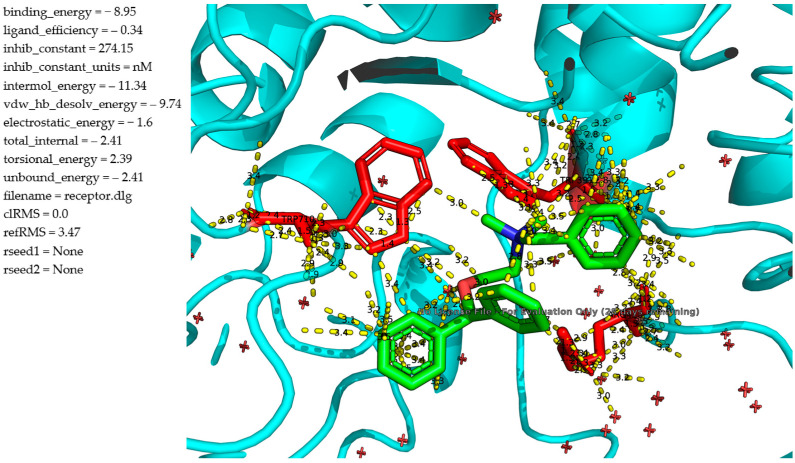
Molecular docking analysis of the binding of **1k** and α-glucosidase.

The docking analysis of **1k** against α-glucosidase (Saccharomyces cerevisiae, PDB ID: 4J5T) reveals enhanced binding characteristics compared to the parent compound diphenhydramine. The ligand **1k** (Figure 2) fits snugly within the enzyme’s active site and forms an extensive network of hydrophobic contacts, π–π interactions, and hydrogen bonds with key residues (the figures are available at Supplementary Materials). Notably, TRP391 and PHE310 participate in π–π stacking with the aromatic rings of the ligand at distances around 3.3–3.5 Å, reinforcing the binding orientation. The positively charged tertiary amine is positioned to engage in electrostatic and hydrogen bonding interactions with surrounding polar residues, including GLU and ASP side chains, at distances of 2.8–3.2 Å, as visualized by multiple interaction vectors. The calculated binding energy of the complex is −8.95 kcal/mol, indicating a stronger interaction than previously observed with the unmodified ligand. The inhibition constant (K_i_) is estimated at 274.15 nM, suggesting moderate affinity, significantly improved over the earlier compound (K_i_ ≈ 917 nM). The intermolecular interaction energy of −11.34 kcal/mol, driven largely by van der Waals/desolvation contributions (−9.74 kcal/mol), further supports the stability of the complex.

**Figure 3:**
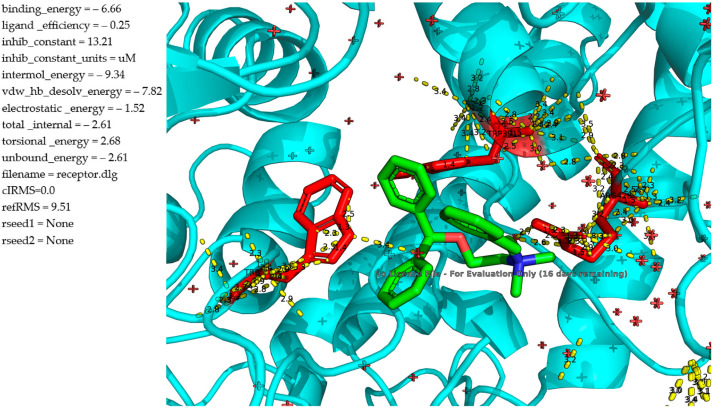
Molecular docking analysis of the binding of **1l** and α-glucosidase.

The molecular docking analysis of **1l** against α-glucosidase reveals comparatively weaker binding. The ligand (Figure 3) occupies the enzyme’s binding pocket and engages in a limited number of stabilizing interactions. π–π stacking with aromatic residues such as TRP391 is still present but appears suboptimal, with increased spatial separation and fewer parallel alignments, suggesting a reduced aromatic overlap compared to previous analogues. Despite an intermolecular energy of −9.34 kcal/mol, the total binding energy is only −6.66 kcal/mol, with a ligand efficiency of 0.25, both lower than those of the methylbenzyl analogue. The predicted inhibition constant (K_i_) is 13.21 μM, indicating a significantly lower affinity and weak inhibition potential. This is consistent with experimental data, which demonstrated negligible α-glucosidase inhibition even at high micromolar concentrations. The decline in activity can be attributed to the increased steric bulk and conformational flexibility introduced by the ethylphenyl chain, which likely prevents optimal ligand orientation and disrupts key interactions within the active site. Additionally, the higher torsional energy (2.68 kcal/mol) suggests a substantial energetic penalty for adopting the bound conformation. These findings indicate that ethylbenzyl modification negatively impacts both binding affinity and biological activity, underlining the importance of steric constraints and electronic alignment in ligand design for α-glucosidase inhibition.

**Figure 4:**
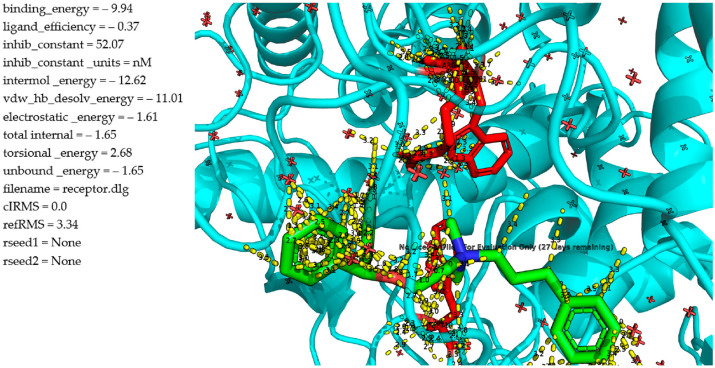
Molecular docking analysis of the binding of **1m** and α-glucosidase.

Compound **1m** shows the most favorable binding profile among all tested analogues when docked to α-glucosidase. The ligand (Figure 4) is deeply embedded within the active site and forms a dense network of stabilizing interactions. Notably, π–π stacking interactions with TRP391 and surrounding aromatic residues occur at ideal distances (~3.2–3.4 Å), while the flexible propyl linker appears to optimize spatial accommodation within the hydrophobic cavity. Multiple hydrogen bonds and van der Waals contacts with polar and hydrophobic side chains contribute to the binding stability, as seen in the dense interaction map with distances ranging from 2.5 to 3.3 Å. The docking results support these observations, with a binding energy of −9.94 kcal/mol, an intermolecular energy of −12.62 kcal/mol, and a van der Waals/desolvation contribution of −11.01 kcal/mol, all indicating a strongly favorable binding pose. Most importantly, the predicted inhibition constant (K_i_) is 52.07 nM, indicating high affinity and potent inhibitory potential in the nanomolar range. This is fully consistent with experimental findings, which confirmed that this derivative exhibits strong α-glucosidase inhibition at low micromolar concentrations. The superior performance of this compound is attributed to the optimal balance between flexibility and hydrophobicity provided by the propylphenyl substituent, which enhances the ligand’s ability to occupy the active pocket without incurring significant torsional strain (torsional energy: 2.68 kcal/mol). These features make this derivative the most promising candidate for further development as an α-glucosidase inhibitor.

AutoDock analysis demonstrated that the binding free energy between **1k** and α-glucosidase (−8.95 kcal/mol) and **1m** and α-glucosidase (−9.94 kcal/mol) is sufficient to provide tight binding, while the binding free energy between **1l** and α-glucosidase (−6.66 kcal/mol) is significantly lower and results in its negligible inhibitory activity.

#### 2.2.2. Inhibitory Activity Against the Enzyme α-Amylase

Antidiabetic activity was assessed by the degree of inhibition of α-amylase activity by the synthesized substances. The study of the degree of inhibition of α-amylase activity by the tested compounds was performed using a standard method with minor modifications [23]. Inhibitory activity was expressed as a percentage (%) of the degree of inhibition of α-amylase in comparison with the negative control, which was calculated by the formula (1).

The results of the study of the inhibitory activity of the tested compounds against the α-amylase enzyme are shown in Table 3.

**Table 3:** Inhibitory activity of the tested compounds against the enzyme α-amylase.

No.	Code	The Degree of Inhibition of the Activity of α-Amylase, %
1	1·HCl	34.7 ± 1.8
2	**1a**	31.8 ± 1.1
3	**1b**	8.7 ± 1.3
4	**1c**	No inhibition
5	**1d**	No inhibition
6	**1e**	No inhibition
7	**1f**	No inhibition
8	**1g**	No inhibition
9	**1h**	No inhibition
10	**1i**	No inhibition
11	**1j**	No inhibition
12	**1k**	No inhibition
13	**1l**	No inhibition
14	**1m**	No inhibition
15	Acarbose	62.0 ± 0.7

Moderate inhibitory activity against α-amylase was observed in the following compounds: 1·HCl (34.7%) and **1a** (31.8%).

Weak inhibitory activity against α-amylase was observed in compound **1b** (8.7%).

Inhibitory activity against α-amylase was absent in the other compounds.

The comparison drug acarbose showed standard inhibitory activity against α-amylase, which was 62.0%.

This test showed that almost all compounds had no inhibitory activity against α-amylase. Only **1a** and **1b** showed moderate and weak inhibitory activity against α-amylase, respectively. Moreover, the inhibitory activity drops drastically with the length/bulkiness of an added group: H > CH_3_ >> C_2_H_5_ >> C_3_H_7_, increasing the bulkiness and the chain length of *N*-substituents has been shown to decrease the inhibitory activity.

### 2.3. Hematopoietic Activity

The hematopoietic activity was studied in Wistar albino female rats. All animals survived up to the end of the experiment: no clinical signs, body weight loss, or deviation of food and water consumption were observed. Thus, no exclusion of animals from the experimental group was conducted (*n* = 6). Intact animals had hematological parameters corresponding to the values of conditionally healthy animals; the total erythrocyte index was (7.09 ± 1.17) × 10^12^/L of blood with hemoglobin (158.5 ± 16.54) g/L of blood. The hematocrit index was (36.95 ± 3.21)%, which is the lower limit of normal values, but blood sampling from animals was carried out in the morning, and 12 h before blood sampling, animals were deprived of food. Therefore, this value is the norm. The total leukocyte index was (10.74 ± 1.11) × 10^9^/L of blood with an absolute value of neutrophils (0.99 ± 0.79) × 10^9^/L of blood and an absolute value of lymphocytes (8.84 ± 1.51) × 10^9^/L of blood, with relative values of neutrophils (9.3 ± 1.05)% and lymphocytes (82.4 ± 3.16)% that fit into the regulatory scale for animals free of pathogenic microflora. The platelet level was (561.2 ± 12.21) × 10^9^/L of blood, which is the optimal indicator. Thus, the main blood counts of white laboratory rats fell within the normative values. After administration of the cytostatic drug (cyclophosphamide), the following changes in the blood hemogram were recorded. The total erythrocyte index from the value of intact animals (7.09 ± 1.17) × 10^12^/L of blood decreased to (4.09 ± 1.64) × 10^12^/L of blood, i.e., by 1.73 times. The hemoglobin value of intact animals decreased from (158.5 ± 16.54) g/L of blood to (71.0 ± 6.04) g/l of blood. The hematocrit index, which shows exactly the percentage of shaped blood elements, decreased by 3.27 times from the value of intact animals (36.95 ± 3.21)% to (11.0 ± 0.31)%. Such a significant decrease in the content of shaped blood elements already indicates a decrease in the number of blood cells. The total leukocyte index decreased by 2.76 times from the level of intact animals (10.74 ± 1.11) × 10^9^/L of blood to (3.88 ± 0.92) × 10^9^/L of blood, with a decrease in the relative values of lymphocytes and an increase in the relative values of monocytes.

Thus, we obtained a decrease in the proliferative activity of erythrocytes, platelets, and leukopoiesis. Further, against the background of erythrocytes, platelets, and leukopenia, newly synthesized compounds and methyluracil (control) were administered intramuscularly to stimulate erythropoiesis, thrombocytopoiesis, and leukopoiesis. The following parameters were analyzed: WBC—white blood cell count; NEU—neutrophil–lymphocyte ratio; LYM—absolute lymphocyte count; MON—monocyte count; EO—eosinophil count; BAS—basophils; RBC—red blood cell count; HGB—hemoglobin; HCT—hematocrit; MCV—mean corpuscular volume; MCH—mean corpuscular hemoglobin; MCHC—mean corpuscular hemoglobin concentration; RDW—red blood cell distribution width; PLT—total platelet volume; MPV—mean platelet volume.

Before testing for hematopoietic activity, the activity of the compounds was predicted using the PASS program [24]. Compounds that showed non-zero activity in the PASS program were subjected to experimental testing for hematopoietic activity (see Supplementary Materials: Table S2). According to the results of the blood hemogram, three compounds—**1f**, **1b**, and **1k**—were the most active and therefore their results are presented in Table 4. The results of the hematopoietic activity of the remaining compounds (**1a**–**1m**) can be found in the Supplementary Materials in Table S3.

Compound **1b** showed the best results. It was more active than all other compounds tested. The total leukocyte index reached a value of (11.25 ± 1.45) × 10^9^/L of blood and was identical to the average value of the intact group. Absolute and relative granulocyte indices were significantly higher than normal. However, the absolute lymphocyte index was (6.91 ± 0.31) × 10^9^/L of blood, which approached the value of the intact group. The total erythrocyte index (RBC indices) was quite high and reached a value of (6.88 ± 0.44) × 10^12^/L, correlating with the value of the intact animal group (7.09 ± 1.17) × 10^12^/L of blood and the value of the control group (7.42 ± 1.12) × 10^12^/L of blood. The hemoglobin level correlated with the value of the intact animal group. The total platelet count in the **1b** compound was quite high (554.5 ± 19.4) × 10^9^/L, which corresponded to the value of the intact animal group (561.2 ± 12.21) × 10^9^/L of blood and was higher than the value of the control group (340.2 ± 26.10) × 10^9^/L of blood. The compound 1·HCl showed comparatively slightly higher activity in this group than all the other compounds. The total leukocyte index reached a value of (7.2–7.3) × 10^9^/L of blood and was identical to the average value of the control group (7.28 ± 1.26) × 10^9^/L of blood, but was lower than the value of intact animals (10.74 ± 1.11) × 10^9^/L of blood by 1.48 times. The absolute values of neutrophils also increased from the values in the intoxication group (1.72 ± 0.18) × 10^9^/L to the values (2.15 ÷ 3.93) × 10^9^/L of blood. This indicator was at the level of the value of the control group (2.17 ± 0.64) × 10^9^/L of blood. Accordingly, the hemoglobin level ranged from (147 to 162) g/L, which also corresponded to the values of the control group. In the groups of administration of compound 1·HCl, the total platelet count ranged from (412.2 ÷ 441.5) × 10^9^/L of blood, almost reaching the value of intact animals (561.2 ± 12.21) × 10^9^/L of blood and exceeding the value of the control group (340.2 ± 26.10) × 10^9^/L of blood.

The same group also included compounds **1f** and **1k**. They were slightly lower than the values of the control group, but not significantly. The total leukocyte count ranged from (6.59 ÷ 6.65) × 10^9^/L of blood, almost corresponding to the value of the control group (7.28 ± 1.26) × 10^9^/L of blood. The absolute values of neutrophils also increased from the value in the intoxication group (1.72 ± 0.18) × 10^9^/L to the value (2.51 ÷ 3.15) × 10^9^/L of blood. But according to the values of the absolute lymphocytic index, the compounds **1f** and **1k** were inferior to the activity of the control drug methyluracil. The lymphocyte level was low and ranged from (2.92 ÷ 3.72) × 10^9^/L of blood, which was significantly lower than the values of the control group and the group of intact animals, but the placebo group values were higher.

The total erythrocyte index (RBC indices) was quite high and reached a value of (6.43 ÷ 7.66) × 10^12^/L, correlating with the value of the intact animal group (7.09 ± 1.17) × 10^12^/L of blood and the value of the control group (7.42 ± 1.12) × 10^12^/L of blood. The hemoglobin level was lower than the values of the control group. Also, the hematocrit index indicated an insufficient rate of cell repair. The total platelet count in the **1f** compound administration group was quite high and amounted to (524.5 ± 18.95) × 10^9^/L, which corresponded to the value of the intact animal group (561.2 ± 12.21) × 10^9^/L of blood and was higher than the value of the control group (340.2 ± 26.10) × 10^9^/L of blood. But in the group of administration of the compound **1k**, the total platelet count was low (290.5 ± 21.44) × 10^9^/L of blood and did not correlate with either the values of the control group or the intact group.

The compounds **1b**, **1f**, and **1k** have the ability to restore immune blood cells (hematopoietic activity), equal to or exceeding that of the commercially available diphenhydramine hydrochloride and control (methyluracil).

## 3. Materials and Methods

### 3.1. Chemical Research Methods

The ionic substance’s m.p. was measured in an open capillary tube using an OptiMelt (Stanford Research System). ^1^H and ^13^C NMR spectra were recorded on a JNM-ECA “Jeol 400” spectrometer (Jeol, Tokyo, Japan) (frequency 399.78 and 100.53 MHz, respectively) and benchtop NMReady-60 (Nanalysis, Calgary, AB, Canada) (frequency 60 and 15 MHz, respectively) using DMSO-*d*_6_ and CDCl_3_ solvents. Chemical shifts were measured relative to the signals of residual protons or carbon atoms of deuterated dimethyl sulfoxide. Mass Spectra were recorded on a Thermo Q Exactive Plus (Orbitrap, Thermo Fisher Scientific, San Jose, CA, USA). Full MS—SIM; Scan Range—160–600 m/z; method: duration, 10 min; UHPLC—Thermo Dionex Ultimate 3000; column—Agilent SB-CB; 2.1/100 mm; RRHD—1.8 um; mobile phase: A: can/B: 0.1% formic acid in H_2_O; flow—0.3 mL/min; gradient conditions. IR spectra were recorded on a «Nicolet 5700 FT-IR» spectrometer (Thermo Fisher Scientific, Waltham, MA, USA). UV spectra were recorded on a «Lambda-35» spectrometer (Perkin Elmer, Waltham, MA, USA). Elemental analysis was conducted on a THERMO FlashSmart CHNS/O elemental analyzer (Thermo Fisher Scientific, Waltham, MA, USA). Thin layer chromatography on selective plates (Sigma Aldrich^®^, St. Louis, MO, USA) with appropriately developed vectors was used to test the product’s purity. The ethylene mixtures (4:1 *v*/*v* and 5:1 *v*/*v*) were used as eluents. The developed plates’ TLC spots were exposed to UV light (λ = 254 nm). A direct current generator (42 kHz, 100 W) and a domestic microwave generator (80 W) were used for the reaction. The separation and purification of substances was carried out by crystallization from appropriate solutions [25].

### 3.2. General Procedure of Synthesis

The initial compound, 2-(diphenylmethoxy)-*N*,*N*-dimethylethanamine (diphenhydramine free base), was synthesized from commercially available hydrochloride by neutralization with potassium carbonate. The 0.01 mol of diphenhydramine hydrochloride was dissolved in 20 mL of water. The initial solution of diphenhydramine hydrochloride had a pH < 7, so potassium carbonate was added till pH = 9. The extraction was carried out three times with benzene. The extract obtained was dried with anhydrous calcium chloride for 12 h. The solvent was removed by simple distillation. The product was dried for two hours in a vacuum at 80 °C. The 0.01 mol of diphenhydramine base was dissolved in 15 mL of acetonitrile in a 100 mL flask. Following that, 0.011 mol of alkyl halides was added, and the mixture was then heated using the traditional procedure (75–82 °C). The same solution mixture was used in different ways. The reaction mixture was put in a US reactor, and its contents were reacted under US conditions, which included 42 kHz and 100 W at 25–35 °C. Meanwhile, the mixture was put in a microwave reactor, and its contents reacted under 80–160 W of microwave irradiation at 25–60 °C [25] (the melting points and yields can be found in Table 2).

2-(benzhydryloxy)-*N*,*N*,*N*-trimethylethanaminium iodide (**1a**). White powder. Anal. for C_17_H_22_ClNO (291.82 g/mol): calcd. C, 69.97; H, 7.60; Cl, 12.15; N, 4.80; O, 5.48; found C, 69.95; H, 7.54; Cl, 12.11; N, 4.74; O, 5.46, %. IR spectrum, υ, cm^−1^: 1152 (C-O), 1527, 1586, 1611 (C_aromatic_ = C_aromatic_), 1173, 1196 (C-N).

2-(benzhydryloxy)-*N*-ethyl-*N*,*N*-dimethylethanaminium iodide (**1b**). White powder. Anal. for C_19_H_26_INO (411.32 g/mol): calcd. C, 55.48; H, 6.37; I, 30.85; N, 3.41; O, 3.89; found C, 55.44; H, 6.35; I, 30.82; N, 3.40; O, 3.82, %. IR spectrum, υ, cm^−1^: 1155 (C-O), 1529, 1576, 1609 (C_aromatic_ = C_aromatic_), 1159, 1189 (C-N).

*N*-(2-(benzhydryloxy)ethyl)-*N*,*N*-dimethylpropan-1-aminium iodide (**1c**). Pale yellow powder. Anal. for C_20_H_28_INO (425.35 g/mol): calcd. C, 56.47; H, 6.64; I, 29.84; N, 3.29; O, 3.76; found C, 56.46; H, 6.60; I, 29.79; N, 3.25; O, 3.73, %. IR spectrum, υ, cm^−1^: 1160 (C-O), 1531, 1580, 1612 (C_aromatic_ = C_aromatic_), 1161, 1199 (C-N).

*N*-(2-(benzhydryloxy)ethyl)-*N*,*N*-dimethylbutan-1-aminium iodide (**1d**). Pale yellow powder. Anal. for C_21_H_30_INO (439.37 g/mol): calcd. C, 57.41; H, 6.88; I, 28.88; N, 3.19; O, 3.64; found C, 57.38; H, 6.83; I, 28.86; N, 3.17; O, 3.60, %. IR spectrum, υ, cm^−1^: 1154 (C-O), 1533, 1574, 1610 (C_aromatic_ = C_aromatic_), 1149, 1175 (C-N).

2-(benzhydryloxy)-*N*-(cyanomethyl)-*N*,*N*-dimethylethanaminium iodide (**1e**). Pale yellow powder. Anal. for C_19_H_23_IN_2_O (422.30 g/mol): calcd. C, 54.04; H, 5.49; I, 30.05; N, 6.63; O, 3.79; found C, 54.01; H, 5.45; I, 30.01; N, 6.59; O, 3.78, %. IR spectrum, υ, cm^−1^: 1161 (C-O), 1546, 1591, 1625 (C_aromatic_ = C_aromatic_), 2210 (C≡N).

2-(benzhydryloxy)-*N*-(2-hydroxyethyl)-*N*,*N*-dimethylethanaminium iodide (**1f**). White powder. Anal. for C_19_H_26_INO_2_ (427.32 g/mol): calcd. C, 53.40; H, 6.13; I, 29.70; N, 3.28; O, 7.49; found C, 53.38; H, 6.10; I, 29.64; N, 3.24; O, 7.45, %. IR spectrum, υ, cm^−1^: 1192, 1215 (C-O), 1539, 1590, 1611 (C_aromatic_ = C_aromatic_), 3390 (O-H).

*N*-(2-(benzhydryloxy)ethyl)-3-hydroxy-*N*,*N*-dimethylpropan-1-aminium iodide (**1g**). Pale yellow powder. Anal. for C_20_H_28_INO_2_ (441.35 g/mol): calcd. C, 54.43; H, 6.39; I, 28.75; N, 3.17; O, 7.25; found C, 54.42; H, 6.35; I, 28.72; N, 3.15; O, 7.21, %. IR spectrum, υ, cm^−1^: 1210, 1250 (C-O), 1542, 1595, 1617 (C_aromatic_ = C_aromatic_), 3365 (O-H).

*N*-(2-(benzhydryloxy)ethyl)-4-hydroxy-*N*,*N*-dimethylbutan-1-aminium iodide (**1h**). Anal. for C_21_H_30_INO_2_ (455.37 g/mol): calcd. C, 55.39; H, 6.64; I, 27.87; N, 3.08; O, 7.03; found C, 55.35; H, 6.61; I, 27.85; N, 3.02; O, 7.01, %. IR spectrum, υ, cm^−1^: 1190, 1230 (C-O), 1544, 1575, 1612 (C_aromatic_ = C_aromatic_), 3350 (O-H).

*N*-(2-(benzhydryloxy)ethyl)-2-ethoxy-*N*,*N*-dimethyl-2-oxoethanaminium iodide (**1i**). Pale yellow powder. Anal. for C_21_H_28_INO_3_ (469.36 g/mol): calcd. C, 53.74; H, 6.01; I, 27.04; N, 2.98; O, 10.23; found C, 53.70; H, 5.98; I, 27.01; N, 2.97; O, 10.20, %. IR spectrum, υ, cm^−1^: 1730 (C=O), 1110, 1230 (C-O), 1522, 1549, 1605 (C_aromatic_ = C_aromatic_).

2-(benzhydryloxy)-*N*-(2-carboxyethyl)-*N*,*N*-dimethylethanaminium iodide (**1j**). Pale yellow powder. Anal. for C_20_H_26_INO_3_ (455.33 g/mol): calcd. C, 52.76; H, 5.76; I, 27.87; N, 3.08; O, 10.54; found C, 52.74; H, 5.73; I, 27.85; N, 3.04; O, 10.52, %. IR spectrum, υ, cm^−1^: 1710 (C=O), 1180, 1225 (C-O), 1531, 1559, 1611 (C_aromatic_ = C_aromatic_), 3105 (O-H).

2-(benzhydryloxy)-*N*-benzyl-*N*,*N*-dimethylethanaminium chloride (**1k**). White powder. Anal. for C_24_H_28_ClNO (381.94 g/mol): calcd. C, 75.47; H, 7.39; Cl, 9.28; N, 3.67; O, 4.19; found C, 75.44; H, 7.359; Cl, 9.27; N, 3.65; O, 4.15, %. IR spectrum, υ, cm^−1^: 1178 (C-O), 1528, 1585, 1605 (C_aromatic_ = C_aromatic_), 1110, 1150, 1180 (C-N).

2-(benzhydryloxy)-*N*,*N*-dimethyl-*N*-phenethylethanaminium iodide (**1l**). White powder. Anal. for C_25_H_30_INO (487.42 g/mol): calcd. C, 61.60; H, 6.20; I, 26.04; N, 2.87; O, 3.28; found C, 61.57; H, 6.17; I, 26.01; N, 2.84; O, 3.25, %. IR spectrum, υ, cm^−1^: 1120 (C-O), 1540, 1560, 1612 (C_aromatic_ = C_aromatic_), 1165, 1188, 1195 (C-N).

*N*-(2-(benzhydryloxy)ethyl)-*N*,*N*-dimethyl-3-phenylpropan-1-aminium iodide (**1m**). White powder. Anal. for C_26_H_32_INO (501.44 g/mol): calcd. C, 62.28; H, 6.43; I, 25.31; N, 2.79; O, 3.19; found C, 62.26; H, 6.42; I, 25.27; N, 2.76; O, 3.16, %. IR spectrum, υ, cm^−1^: 1142 (C-O), 1511, 1565, 1613 (C_aromatic_ = C_aromatic_), 1156, 1205, 1210 (C-N).

### 3.3. Structural Confirmation via UV Analysis

UV spectra were recorded in the UV region of 190 to 400 nm using 10^−5^ M solutions of **1a**–**1m**. The maximum absorption bands and the extinction coefficients are presented in Table 5.

Spectra images can be found in the Supplementary Materials section (Figures S40 and S41: UV spectra of obtained compounds).

### 3.4. Structural Confirmation via NMR Analysis

Spectra images can be found in the Supplementary Materials (Figures S1–S26: ^1^H and ^13^C NMR spectra of obtained compounds).

2-(benzhydryloxy)-*N*,*N*,N-trimethylethanaminium iodide (**1a**)

In the ^1^H NMR (400 MHz, DMSO-*d*_6_) spectrum of compound **1a**, the trimethylammonium protons appeared as a nine-proton singlet at 3.11 ppm. In this region of the spectrum, the ethylene protons H-9 and H-10 also resonated with two-proton multiplets at 3.60–3.62 and 3.74–3.76 ppm, respectively. The methine proton H-7 was detected as the expected singlet at 5.60 ppm. The phenyl protons of the compound appeared in the aromatic region of the spectrum as two multiplets at 7.22–7.25 (2H, H-4, 16) and 7.30–7.36 (8H, H-2, 3, 5, 6, 14, 15, 16, 17) ppm.

In the ^13^C NMR (101 MHz, DMSO-*d*_6_) spectrum of compound **1a**, signals of trimethylammonium carbon nuclei are observed at 53.74 (C-12, 19, 20) ppm. Methylene carbons appeared at 62.80 (C-10) and 65.27 (C-19) ppm. Carbon C-7 resonated at 83.18 ppm. Carbon atoms of aromatic nuclei resonated at 127.09 (C-2, 6, 14, 16), 128.10 (C-4, 16), 129.06 (C-3, 5, 15, 17) and 142.22 (C-1, 13) ppm.

2-(benzhydryloxy)-*N*-ethyl-*N*,*N*-dimethylethanaminium iodide (**1b**)

In the ^1^H NMR (400 MHz, DMSO-*d*_6_) spectrum of compound **1b**, the N-ethyl protons H-21 and H-20 appeared as a three-proton triplet at 1.33 with ^3^J 7.3 Hz and a two-proton quadruplet at 3.68 ppm with ^3^J 7.3 Hz, respectively. The *N*-methyl protons H-12, 19 resonated as a six-proton singlet at 3.28 ppm. The ethylene protons H-9 and H-10 appeared as a four-proton multiplet at 3.85–3.86 ppm. The expected singlet at 5.49 ppm was the methine proton H-7. The phenyl protons of the compound appeared in the aromatic region of the spectrum as two multiplets at 7.19–7.24 (2H, H-4, 16) and 7.25–7.30 (8H, H-2, 3, 5, 6, 14, 15, 17, 18) ppm.

In the ^13^C NMR (101 MHz, DMSO-*d*_6_) spectrum of compound **1b**, the signals of the *N*-ethyl-*N*,*N*-dimethylammonium carbon nuclei were observed at 9.01 (C-21), 51.76 (C-12, 19), and 61.57 (C-20) ppm. The methylene carbons appeared at 62.59 (C-10) and 62.98 (C-9) ppm. The C-7 carbon resonated at 84.34 ppm. Carbon atoms of aromatic nuclei resonated at 126.92 (C-2, 6, 14, 18), 128.13 (C-4, 16), 128.81 (C-3, 5, 15, 17), and 140.72 (C-1, 13) ppm.

N-(2-(benzhydryloxy)ethyl)-*N*,*N*-dimethylpropan-1-aminium iodide (**1c**)

In the ^1^H NMR (400 MHz, CDCl_3_) spectrum of compound **1c**, the *N*-propyl protons H-22, H-21, and H-20 appeared as a three-proton triplet at 0.84 ppm with ^3^J 7.3 Hz and two two-proton multiplets at 1.67–1.77 and 3.47–3.51 ppm, respectively. For the *N*-methyl protons H-12, 19 resonated as a 6-proton singlet at 3.28 ppm. The ethylene protons H-9 and H-10 appeared as a four-proton multiplet at 3.82–3.84 ppm. The expected singlet at 5.47 ppm was the methine proton H-7. The phenyl protons of the compound appeared in the aromatic region of the spectrum as two multiplets at 7.16–7.21 (2H, H-4, 16) and 7.22–7.28 (8H, H-2, 3, 5, 6, 14, 15, 17, 18) ppm.

In the ^13^C NMR (101 MHz, CDCl_3_) spectrum of compound **1c**, the signals of the *N*-propyl-*N*,*N*-dimethylammonium carbon nuclei were observed at 10.47 (C-22), 16.58 (C-21), 15.38 (C-12, 19), and 67.14 (C-20) ppm. The methylene carbons appeared at 62.62 (C-10) and 63.378 (C-9) ppm. The C-7 carbon resonated at 84.30 ppm. Carbon atoms of aromatic nuclei resonated at 126.93 (C-2, 6, 14, 18), 128.11 (C-4, 16), 128.93 (C-3, 5, 15, 17), and 140.70 (C-1, 13) ppm.

N-(2-(benzhydryloxy)ethyl)-*N*,*N*-dimethylbutan-1-aminium iodide (**1d**)

In the ^1^H NMR (400 MHz, CDCl_3_) spectrum of compound **1d**, *N*-butyl protons H-23, H-22, H-21, and H-20 appeared as a three-proton triplet at 0.89 ppm with ^3^J 7.3 Hz and three two-proton multiplets at 1.23–1.32, 1.65–1.74, and 3.54–32.59 ppm, respectively. *N*-methyl protons H-12, 19 resonated as a six-proton singlet at 3.35 ppm. Ethylene protons H-9 and H-10 appeared as a four-proton multiplet at 3.86–3.93 ppm. The expected singlet at 5.48 ppm was the methine proton H-7. The phenyl protons of the compound appeared in the aromatic region of the spectrum as two multiplets at 7.22–7.25 (2H, H-4, 16) and 7.27–7.32 (8H, H-2, 3, 5, 6, 14, 15, 17, 18) ppm.

In the ^13^C NMR (101 MHz, CDCl_3_) spectrum of compound **1d**, the signals of the *N*-butyl-*N*,*N*-dimethylammonium carbon nuclei are observed at 13.78 (C-23), 19.52 (C-22), 24.89 (C-21), 52.33 (C-12, 19), and 65.83 (C-20) ppm. The methylene carbons appeared at 62.67 (C-10) and 63.33 (C-9) ppm. At 84.50 ppm, carbon C-7 resonated. Carbon atoms of aromatic nuclei resonated at 126.94 (C-2, 6, 14, 18), 128.18 (C-4, 16), 128.80 (C-3, 5, 15, 17), and 140.62 (C-1, 13) ppm.

2-(benzhydryloxy)-*N*-(cyanomethyl)-*N*,*N*-dimethylethanaminium iodide (**1e**)

In the ^1^H NMR (400 MHz, CDCl_3_) spectrum of compound **1e**, the methyl protons H-19, 19, 19, 20, 20, 20) appeared as a six-proton singlet at 3.56 ppm. The methylene protons resonated as multiplets at 3.86–3.88 (2H, H-16, 16) and 4.11–4.13 (2H, H-15, 15) ppm. Closest protons to the nitrile group showed as a singlet at 5.39 (2H, H-18,18) ppm. The tertiary proton H-7 resonated as a single-proton singlet at 5.48 ppm. Phenyl protons appeared in the aromatic zone as a multiplet at 7.18–7.30 (10H, H-1, 3, 4, 5, 6, 10, 11, 12, 13, 14) ppm.

In the ^13^C NMR (101 MHz, CDCl_3_) spectrum of compound **1e**, the signals of the aliphatic carbon nuclei are observed at 52.81 (C-19, 20), 53.97 (C-18), 62.25 (C-16), 64.97 (C-15), and 84.72 (C-7) ppm. The carbon atom of the nitrile group showed at 110.88 ppm. Carbon atoms of aromatic nuclei resonated at 126.92 (C-1, 3, 10, 14), 128.39 (C-5, 12), 128.99 (C-4, 6, 11, 13), and 140.22 (C-2, 9) ppm.

2-(benzhydryloxy)-N-(2-hydroxyethyl)-N,N-dimethylethanaminium iodide (**1f**)

In the ^1^H NMR (400 MHz, CDCl_3_) spectrum of compound **1f**, the methyl protons H-12, 12, 12, 19, 19, 19) appeared as a six-proton singlet at 3.34 ppm. The methylene protons resonated as multiplets at 3.74–3.76 (2H, H-9, 9), 3.86–3.88 (4H, H-10, 10, 20, 20), and 4.07–4.10 (2H, H-21, 21) ppm. In the region of 4.07–4.10 ppm, the hydroxyl protons H-22 also appeared as a single-proton multiplet. The tertiary proton H-7 resonated as a single-proton singlet at 5.49 ppm. Phenyl protons appeared in the aromatic zone as multiplets at 7.23–7.25 (2H, H-4, 16) and 7.29–7.32 (H-2, 6, 14, 18, 3, 5, 15, 17) ppm.

In the ^13^C NMR (101 MHz, CDCl_3_) spectrum of the compound **1f**, signals of aliphatic carbon nuclei are observed at 53.44 (C-12, 19), 55.86 (C-21), 62.66 (C-10), 65.02 (C-9), 66.09 (C-20), and 84.41 (C-7) ppm. Carbon atoms of aromatic nuclei resonated at 126.91 (C-2, 6, 14, 18), 128.23 (C-4, 16), 128.88 (C-3, 5, 15, 17), and 140.69 (C-1, 13) ppm.

N-(2-(benzhydryloxy)ethyl)-3-hydroxy-*N*,*N*-dimethylpropan-1-aminium iodide (**1g**)

In the ^1^H NMR (400 MHz, CDCl_3_) spectrum of compound **1g**, the methyl protons H-12, 12, 12, 19, 19, 19) appeared as a six-proton singlet at 3.27 ppm. The methylene protons resonated as multiplets at 1.97–2.04 (2H, H-21, 21), 3.53–3.64 (2H, H-22, 22), 3.74–3.78 (4H, H-9, 9, 20, 20), and 3.82–3.86 (2H, H-10, 10) ppm. The hydroxyl protons H-23 also appeared as a single-proton multiplet in the region of 1.97–2.04 ppm. The tertiary proton H-7 resonated as a single-proton singlet at 5.50 ppm. Phenyl protons appeared in the aromatic zone as multiplets at 7.23–7.25 (2H, H-4, 16) and 7.29–7.31 (8H, H-2, 6, 14, 18, 3, 5, 15, 17) ppm.

In the ^13^C NMR (101 MHz, CDCl_3_) spectrum of compound **1g**, signals of aliphatic carbon nuclei are observed at 26.13 (C-21), 52.41 (C-12, 19), 58.20 (C-22), 63.49 (C-20), 63.90 (C-10), 64.50 (C-9), and 84.43 (C-7) ppm. Carbon atoms of aromatic nuclei resonated at 126.98 (C-2, 6, 14, 18), 128.21 (C-4, 16), 128.88 (C-3, 5, 15, 17), and 140.68 (C-1, 13) ppm.

N-(2-(benzhydryloxy)ethyl)-4-hydroxy-*N*,*N*-dimethylbutan-1-aminium iodide (**1h**)

The ^1^H NMR (400 MHz, DMSO-*d*_6_) spectrum of compound **1h** is characterized by the presence of a six-proton singlet signal of methyl protons H-12, 12, 12 and H-19, 19, 19 at 3.02 ppm. Methylene protons H-21, 21, H-9, 9, and H-10, 10 were recorded as two-proton singlets at 1.60, 3.57, and 3.75 ppm, respectively. The methine proton H-7 resonated as a one-proton singlet at 5.62 ppm. Aromatic protons H-2-6 and H-14-18 were recorded as a ten-proton multiplet at 7.20–7.36 ppm and a singlet at 6.72 ppm. The hydroxyl proton H-24 appeared as a singlet at 2.74 ppm. The remaining methylene protons of the oxybutyl fragment are possibly present together with the solvent signal at 3.32 ppm.

In the ^13^C NMR (101 MHz, DMSO-*d*_6_) spectrum of compound **1h**, distinct signals of carbon atoms appeared at 83.26 (C-7), 127.02 (C-3, 5, 15, 17), 129.16 (C-2, 6, 14, 18), and 142.11 (C-1,3) ppm. The remaining carbon atoms were determined by heteronuclear correlation of the spectra.

N-(2-(benzhydryloxy)ethyl)-2-ethoxy-*N*,*N*-dimethyl-2-oxoethanaminium iodide (**1i**)

In the ^1^H NMR (400 MHz, CDCl_3_) spectrum of compound **1i**, the methyl protons H-25, 25, 25 of the ethylate group appeared as a six-proton multiplet signal at 1.07–0.010 ppm. The methylene protons of this fragment appeared as a two-proton quartet-like multiplet at 3.97–4.02 ppm. The *N*-methyl protons H-12, 12, 12, 19, 19, 19 resonated as a six-proton singlet at 3.62 ppm. The methylene protons H-20, 20, which do not have neighboring protons, appeared as a two-proton singlet at 4.68 ppm. The neighboring methylene protons H-9, 9 and H-10, 10 were registered as two-proton broadened singlets at 3.84 and 4.20 ppm, respectively. The one-proton singlet signal at 5.46 ppm belongs to the H-7 proton. The ten-proton multiplet signal at 7.20–7.29 ppm contains aromatic protons H-2-6, 14–18.

In the ^13^C NMR (101 MHz, CDCl_3_) spectrum of compound **1i**, the signals of aliphatic carbon nuclei are observed at 13.87 (C-25), 53.17 (C-12,19), 62.24 (C-20), 62.58 (C-10), 63.47 (C-9), and 84.48 (C-7) ppm. The carbon atoms of the aromatic nuclei resonated at 126.95 (C-2, 6, 14, 18), 128.21 (C-4, 16), 128.78 (C-3, 5, 15, 17), and 140.44 (C-1, 13) ppm. The carboxyl carbon atom C-21 appeared at 164.47 ppm.

2-(benzhydryloxy)-*N*-(2-carboxyethyl)-*N*,*N*-dimethylethanaminium iodide (**1j**)

In the ^1^H NMR (400 MHz, DMSO-*d*_6_) spectrum of compound **1j**, phenyl protons appeared in the aromatic zone as a multiplet at 7.21–7.40 (10H, H-2, 3, 4, 5, 6, 9, 10, 11, 12, 13) ppm. The tertiary proton H-7 resonated as a single-proton singlet at 5.60 ppm. The methylene protons resonated at 2.30 (H-19, 19), 2.67–2.75 (2H, H-18, 18), 3.09 (6H, H-22, 23), 3.60–3.64 (2H, H-15, 15), 3.77–3.79 (2H, H-16, 16) ppm. The carboxylic proton showed at 4.80 (H-21) ppm.

In the ^13^C NMR (101 MHz, DMSO-*d*_6_) spectrum of compound **1j**, the signals of the aliphatic carbon nuclei are observed at 28.74 (C-19), 50.74 (C-22), 50.91 (C-23), 60.96 (C-18), 62.06 (C-16), 62.81 (C-15), and 82.79 (C-7). Carbon atoms of aromatic nuclei resonated at 126.35–127.55 (C-4, 11), 128.30–128.50 (C-3, 5, 10, 12), 141.55 (C-1, 8), and carboxylic carbon showed at 171.17 (C-20).

2-(benzhydryloxy)-*N*-benzyl-*N*,*N*-dimethylethanaminium chloride (**1k**)

In the ^1^H NMR (CDCl_3_, 60 MHz) spectrum of compound **1k**, the methyl protons appeared at 3.16 (H-25, 26), 3.82 (H-15, 16), 4.92 (H-18), 5.33 ppm (H-7). Phenyl protons appeared in the aromatic zone at 7.14–7.47 (H-2, 3, 4, 5, 6, 9, 10, 11, 12, 13, 20, 21, 22, 23, 24) ppm.

In the ^13^C NMR (CDCl_3_, 15 MHz) spectrum of the compound, the signals of the aliphatic carbon nuclei are observed at 50.32 (C-25, 26), 62.84 (C-15, 16), 68.78 (C-18), and 84.48 (C-7) ppm. Carbon atoms of aromatic nuclei resonated at 126.83 (C-2, 6, 9, 13), 127.55 (C-22), 127.91 (C-4, 11), 128.59 (C-3, 5, 10, 12), 128.97 (C-21, 23), 130.47 (C-19), 133.38 (C-20, 24), and 140.62 (C-1, 8).

2-(benzhydryloxy)-*N*,*N*-dimethyl-*N*-phenethylethanaminium iodide (**1l**)

In the ^1^H NMR (400 MHz, CDCl_3_) spectrum of compound **1l**, aliphatic protons appeared as a six-proton singlet at 3.46 ppm, four two-proton singlets at 3.92 (H-21), 4.04 (H-20), 4.21 (H-10), and 4.41 (H-9) ppm, and a one-proton singlet at 5.47 (H-7) ppm. The phenyl protons of the compound appeared in the aromatic region of the spectrum as three multiplets at 6.84–6.86 (2H, H-4, 16), 6.96–6.99 (1H, H-2), and 7.22–7.28 (12H, H-2, 3, 5, 6, 14, 15, 17, 18, 23, 24, 26, 27) ppm.

In the ^13^C NMR (101 MHz, CDCl_3_) spectrum of compound **1l**, the signals of the aliphatic carbon nuclei are observed at 52.95 (C-12, 19), 62.23 (C-9), 62.85 (C-20), 64.33 (C-21), 65.09 (C-10), and 84.52 (C-7) ppm. Carbon atoms of aromatic nuclei resonated at 114.55 (C-4, 16), 122.25 (C-25), 126.96 (C-2, 6, 14, 18), 128.16 (C-23, 27), 128.81 (C-3, 5, 15, 17), 129.93 (C-24, 26), 140.69 (C-1, 13), and 157.04 (C-22) ppm.

N-(2-(benzhydryloxy)ethyl)-*N*,*N*-dimethyl-3-phenylpropan-1-aminium iodide (**1m**)

In the ^1^H NMR (400 MHz, CDCl_3_) spectrum of compound **1m**, aliphatic protons appeared as two-proton multiplets at 2.04–2.08 (H-21, 21), 3.59–3.63 (H-20, 20), two broadened singlets at 3.80 (H-9, 9) and 3.90 (H-10, 10), one-proton at 5.41 (H-7) and six-proton singlets at 3.32 (H-12, 12, 12, 19, 19, 19), and a two-proton triplet at 2.59 ppm (H-22, 22, 3J 7.6 Hz). The phenyl protons of the compound appeared in the aromatic zone as multiplets at 7.10–7.12 (H-24, 26) and 7.17–7.34 (H-2-6, H-14-18, H-25-27) ppm.

In the ^13^C NMR (101 MHz, CDCl_3_) spectrum of compound **1m**, the signals of the aliphatic carbon nuclei are observed at 29.93 (C-21), 32.01 (C-22), 52.51 (C-12, 19), 62.03 (C-10), 63.35 (C-9), 5.06 (C-20), and 84.52 (C-7) ppm. Carbon atoms of aromatic nuclei resonated at 127.00 (C-26, 24, 28, 4, 6), 128.50 (C-2, 6, 14, 18, 25, 27), 128.85 (C-3, 5, 15, 17), 139.45 (C-23), and 140.52 (C-1, 13) ppm.

### 3.5. Structural Confirmation via MASS Spectrometry

Spectrum images can be found in the Supplementary Materials (Figures S27–S39: mass spectra of obtained compounds).

The base peak for the cation [C_18_H_24_NO]^+^ (**1a**) was observed at 270.18491 m/z (calc. 270.185789). The base peak for the cation [C_19_H_26_NO]^+^ (**1b**) was observed at 284.20056 m/z (calc. 284.201439). The base peak for the cation [C_20_H_28_NO]^+^ (**1c**) was observed at 298.21617 m/z (calc. 298.217089). The base peak for the cation [C_21_H_30_NO]^+^ (**1d**) was observed at 312.23181 m/z (calc. 312.232739). The base peak for the cation [C_19_H_23_N_2_O]^+^ (**1e**) was observed at 295.18035 m/z (calc. 295.181038). The base peak for the cation [C_19_H_26_NO_2_]^+^ (**1f**) was observed at 300.19550 m/z (calc. 300.196354). The base peak for the cation [C_20_H_28_NO_2_]^+^ (**1g**) was observed at 314.21111 m/z (calc. 314.212004). The base peak for the cation [C_21_H_30_NO_2_]^+^ (**1h**) was observed at 328.22674 m/z (calc. 328.227654). The base peak for the cation [C_21_H_28_NO_3_]^+^ (**1i**) was observed at 342.20611 m/z (calc. 342.206919). The base peak for the cation [C_20_H_26_NO_3_]^+^ (**1j**) was observed at 328.19029 m/z (calc. 328.191269). The base peak for the cation [C_24_H_28_NO]^+^ (**1k**) was observed at 346.21617 m/z (calc. 346.217089). The base peak for the cation [C_25_H_30_NO]^+^ (**1l**) was observed at 360.23188 m/z (calc. 360.232739). The base peak for the cation [C_26_H_32_NO]^+^ (**1m**) was observed at the 374.24745 m/z (calc. 374.248389).

### 3.6. Biological Research Methods

#### 3.6.1. Experiment on the Inhibitory Activity Against the Enzyme α-Glucosidase

A reaction mixture containing 500 µL of phosphate buffer (0.1 M, pH 6.8) (1 unit/mL) was added to 100 µL of α-glucosidase and 200 µL of the test sample solution (15 mm). The resulting mixture was incubated for 15 min at 37 °C, then 200 µL of a solution of 4-Nitrophenyl α-D-glucopyranoside (p-Nitrophenyl α-D-glucopyranoside, P-NPG) (5 mM) was added, after which it was incubated at 37 °C for 20 min. Then the reaction was stopped by adding 500 µL of sodium carbonate (0.1 M). The solution was used as a form of α-glucosidase (1 unit/mL). A solvent was used as a negative control at 200 µL in four repetitions.

#### 3.6.2. Experiment on the Inhibitory Activity Against the Enzyme α-Amylase

A reaction mixture containing 50 µL of phosphate buffer (100 mM, pH = 7.2), 10 µL of α-amylase (2 units/mL) and 20 µL of the tested compounds at a concentration of 150 µM/mL was added to each well of the 96-well tablet, which was then incubated at 37 °C for 30 min. Then, 50 µL of 1% soluble starch (100 mM phosphate buffer, pH = 7.2) was added as a substrate and incubated at 37 °C for 10 min. After that, 100 µL of DNS staining reagent was added and boiled for 5 min. The optical density of the resulting mixture was measured at 540 nm on a flatbed spectrophotometer. Acarbose at a concentration of 150 µM/mL was used as a comparison drug (positive control). In parallel, a negative control (20 µL of solvent) was performed without the addition of the tested compounds. All samples were examined in triplicate.

#### 3.6.3. Animal Studies: Hematopoietic Activity

Adult Wistar rats (single animal) were chosen for the test model of this experiment due to their physiological similarity with humans. All experimental procedures that involve animals were carried out in compliance with the “Guide for the Care and Use of Laboratory Animals” and ARRIVE guidelines, as well as the Law of the Republic of Kazakhstan dated 4 March 2022, No. 45-r, “On Responsible Treatment of Animals”. The study protocol was reviewed and approved by the Local Ethics Commission of the Al-Farabi Kazakh National University, No. IRB-A498 (IRB00010790 Al-Farabi Kazakh National University IRB #1) dated 21.10.2022. Animals were maintained under standard laboratory conditions: a controlled temperature of 24 ± 2 °C, relative humidity of 35–60%, and a 12 h light/dark cycle with free access to standard chow and water at the animal facility. They were housed in polypropylene cages, 3–5 animals per cage, and were acclimatized for 7 days before the start of the experiment.

Animals were monitored daily. Any clinical signs, deviation of food and water intake, and/or mortality observed in animals were set a priori as criteria for their exclusion from the experiment.

One hundred and two Wistar albino rats (8–10 weeks old, 250 ± 20 g) were used for the study of the hematopoietic-stimulating activity of new compounds (**1a**–**1m**). Before the start of the experiment, animals were randomly assigned into 18 groups (six animals per group, *n* = 6) as follows: intact (UT), placebo (PL), control (MU), 1·HCl group (1·HCl), 1f group (**1f**), 1b group (**1b**), 1k group (**1k**), etc. Animal procedures were performed at 9.00 am, and the order of each animal used was randomized daily. The administration procedure was conducted by one investigator, who was the only person aware of the treatment received by each group.

A priori sample size was calculated to be 3–6 animals per group, since it is a recommended number of animals used for in vivo studies from the validity as well as bioethics point of view.

Hematopoietic suppression was induced by the administration of the cytostatic drug cyclophosphamide (Baxter Oncology GmbH). On the 1st, 3rd, and 5th days of the experiment, animals of the PL, MU, 1·HCl, and **1a**–**1m** groups were intramuscularly injected with a 3% solution of sodium cyclophosphamide at a dose of 30 mg/kg (solvent: isotonic saline sodium chloride solution). The average volume of the administered drug was 0.2–0.24 mL. Then, on the 6th, 7th and 8th days of the experiment, at 9:00 a.m., the 1·HCl, and **1a**–**1m** compounds were injected into the animals of the 1·HCl, ad **1a**–**1m** groups, respectively (the compounds were dissolved in saline solution, administered intramuscularly at a dose of 10 mg/kg, in a volume of 0.2–0.25 mL (1% solution)), the control group (MU) animals were injected with 6-methyluracil (dissolved in saline solution and administered intramuscularly at a dose of 10 mg/kg in a volume of 0.2–0.25 mL (1% solution)), and animals of the placebo group (PL) were injected with a saline solution of 0.2–0.25 mL. Compounds were not injected into the intact (untreated) group (UT) of animals. On the 15th day of the experiment (7 days after the last injection), blood was taken at 9:00 a.m. from the orbital sinus of rats (under light anesthesia with ketamine/xylazine (91.0 mg/kg ketamine/9.1 mg/kg xylazine)) into hematology tubes VF-052SDK (2 mL) with EDTA (K2). The feed was removed from the feeders 12 h before blood collection. Blood tests were performed on the hematological analyzer of animal blood “MicroCC-20 Plus” (China). For double cytological control, blood smears were performed to count the leukogram of the blood. Blood smears were stained using the Giemsa method and were counted under a Leica microscope (magnification 7 × 100) with an immersion of 100 cells in each smear sample, after which the relative number of cells of each type was converted to an absolute value [26,27].

### 3.7. Molecular Docking Study

The binding mode of *N*-alkylphenyl derivatives of diphenhydramine to α-glucosidase was investigated by AutoDock 4.2 simulation software with the graphical interface AutoDock Tools (ADT) version 1.5.7 developed by the Scripps Research Institute. The crystal structure of α-glucosidase was downloaded from the RCSB PDB database [28]. The molecular structures of N-alkylphenyl derivatives of diphenhydramine were optimized using density functional theory (B3LYP/6-31G+).

### 3.8. Statistical Data Processing

The data obtained were processed by mathematical statistics methods using Microsoft Excel and the “Statistica 6.0” software. The data are presented as an average value (M) ± standard deviation (SD) (*n* = 6 rats/group). We used one-way (single factor) ANOVA (analysis of variance) to determine a statistically significant difference, and the values were considered reliable at *p* < 0.05 (95% confidence interval) and F > F_crit_.

## 4. Conclusions

The results of the experiments performed have confirmed that microwave and ultrasound activation can effectively compete with conventional methods of synthesis in N-alkylation reactions. The findings of our investigation support published data indicating a trend for a rise in the efficacy of supporting the reaction in a certain order using the classical approach, ultrasound-assisted synthesis, and microwave irradiation. In some reactions, ultrasound activation results in a somewhat lower product yield than under standard conditions, but with a threefold reduction in synthesis time. Iodomethane, iodoacetonitrile, and benzyl chloride showed higher yields, with greater reactivity as alkylating agents. The ionic compounds **1k** and **1m** had a better inhibitory property than the reference medication acarbose, and five of the synthesized derivatives of diphenhydramine demonstrated similar or higher inhibitory action against α-glucosidase than acarbose. The hematopoietic activity of novel synthesized compounds **1b**, **1f**, and **1k** was similar or higher than that of the commercially available diphenhydramine hydrochloride and control (methyluracil). The *N*-benzyl derivative of diphenhydramine **1k** showed the most promising results (due to the highest antidiabetic and significant hematopoietic activities contemporaneously) and can be recommended for further investigations as a dual-target hematopoietic-stimulating and antidiabetic agent. This research may serve as a roadmap for the synthesis of new smart chemicals with antidiabetic and hematopoietic activity. Thus, the findings of this study are promising in a preclinical animal model; further validation through clinical trials in human populations is required.

## Figures and Tables

**Table 1:** The parameters of *N*-alkylation reaction.

Products	Reaction Conditions *	Time, Min	Yield, %	Melting Point
**1a**	Classical	20	85	205–207 °C
-CH_3_	US	10	81	
	MW	1	89	
**1b**	Classical	60	78	161–163 °C
-C_2_H_5_	US	30	62	
	MW	3	79	
**1c**	Classical	120	81	145–147 °C
-n-C_3_H_7_	US	40	65	
	MW	4	83	
**1d**	Classical	180	81	141–143 °C
-n-C_4_H_9_	US	60	70	
	MW	7	96	
**1e**	Classical	90	85	128–130 °C
-CH_2_CN	US	30	45	
	MW	3	96	
**1f**	Classical	180	91	106–108 °C
-CH_2_CH_2_OH	US	60	80	
	MW	6	93	
**1g**	Classical	180	87	105–107 °C
-(CH_2_)_3_OH	US	60	79	
	MW	6	89	
**1h**	Classical	210	81	104–106 °C
-(CH_2_)_4_OH	US	120	71	
	MW	15	85	
**1i**	Classical	180	93	147–149 °C
-CH_2_COOEt	US	60	81	
	MW	10	94	
**1j**	Classical	300	81	108–110 °C
-(CH_2_)_3_COOH	US	120	71	
	MW	30	85	
**1k**	Classical	120	86	121–123 °C
-CH_2_C_6_H_5_	US	60	74	
	MW	30	85	
**1l**	Classical	350	76	116–118 °C
-CH_2_CH_2_C_6_H_5_	US	150	69	
	MW	50	85	
**1m**	Classical	350	78	104–106 °C
-(CH_2_)_3_C_6_H_5_	US	150	72	
	MW	50	86	

* Classical conditions (thermal activation): reflux at 82 °C. Ultrasound activation: 42 kHz and 100 W at 25–35 °C. Microwave activation: 80–160 W at 25–60 °C.

**Table 2:** Inhibitory activity of the tested compounds against the enzyme α-glucosidase.

No.	Code	The Degree of Inhibition of the Activity of α-Glucosidase, %
1	1·HCl	No inhibition
2	**1a**	13.4 ± 1.6
3	**1b**	12.0 ± 1.6
4	**1c**	28.9 ± 3.1
5	**1d**	35.7 ± 4.4
6	**1e**	49.0 ± 0.8
7	**1f**	49.8 ± 1.1
8	**1g**	38.9 ± 1.1
9	**1h**	27.8 ± 2.9
10	**1i**	50.2 ± 1.2
11	**1j**	No inhibition
12	**1k**	64.2 ± 1.3
13	**1l**	20.7 ± 2.1
14	**1m**	66.9 ± 2.3
15	Acarbose	51.8 ± 2.1

**Table 4:** Hemogram parameters of peripheral blood in rats, M ± m (*n* = 6).

Parameters	1·HCl	1f	1b	1k	Control	Placebo	Intact
WBC·10^9^/L	7.22 ± 2.21 *****	6.59 ± 1.31	11.25 ± 1.45	6.65 ± 1.12	7.28 ± 1.26	3.88 ± 0.92 ****	10.74 ± 1.11
NEU·10^9^/L	2.15 ± 1.15	3.15 ± 0.90 ******	3.44 ± 0.68	2.51 ± 0.60	2.17 ± 0.64	1.72 ± 0.18	0.99 ± 0.79
LYM·10^9^/L	4.67 ± 1.07	2.92 ± 0.30 *******	6.91 ± 0.31	3.72 ± 1.1 ********	4.57 ± 0.19	1.57 ± 0.13	8.84 ± 1.51
MON·10^9^/L	0.3 ± 0	0.19 ± 0	0.34 ± 0	0.27 ± 0	0.31 ± 0	0.47 ± 0.24	0.29 ± 0
EO·10^9^/L	0.03 ± 0	0.17 ± 0	0.41 ± 0	0.06 ± 0	0.2 ± 0	0.07 ± 0	0.38 ± 0
BAS·10^9^/L	0.08 ± 0	0.17 ± 0	0.12 ± 0	0.03 ± 0	0.03 ± 0	0.03 ± 0	0.2 ± 0
NEU%	29.8 ± 0.24	47.6 ± 1.20	30.6 ± 0.15	37.5 ± 1.10	29.8 ± 0.65	44.4 ± 1.61	9.3 ± 1.05
LYM%	64.7 ± 1.75	44.0 ± 0.96	61.5 ± 1.5	56.4 ± 0.8	62.8 ± 1.75	40.6 ± 1.44	82.4 ± 3.16
MON%	4.05 ± 0.75	3.9 ± 0.94	3.1 ± 0.04	4.45 ± 0.41	4.2 ± 0.72	12.2 ± 0.92	2.7 ± 0.07
EO%	0.5 ± 0	2.4 ± 0.2	3.65 ± 0.15	1.05 ± 0	2.8 ± 0	2.0 ± 0	3.5 ± 0.85
BAS%	1.3 ± 0	2.1 ± 0.42	1.1 ± 0	0.65 ± 0	0.4 ± 0	0.8 ± 0	2.0 ± 0
RBC 10^12^/L	8.3 ± 0.74	7.66 ± 0.17	6.88 ± 0.44	6.43 ± 1.31	7.42 ± 1.12	4.09 ± 1.64 *	7.09 ± 1.17
HGB. g/L	162.0 ± 13.2	146.4 ± 16.9	126.0 ± 11.6	125.5 ± 13.1	139.6 ± 2.2	71.0 ± 6.04 **	158.5 ± 16.5
HCT%	37.8 ± 1.82	33.1 ± 0.91	28.45 ± 1.15	28.1 ± 1.01	30.2 ± 2.34	11.0 ± 0.31 ***	36.95 ± 3.21
MCV	45.5 ± 1.15	43.3 ± 2.32	41.3 ± 1.54	43.7 ± 1.71	40.8 ± 1.02	26.9 ± 1.62	43.5 ± 2.31
MCH	19.6 ± 0.6	19.1 ± 1.51	18.25 ± 0.95	19.65 ± 1.42	18.7 ± 1.03	17.4 ± 0.02	19.45 ± 1.65
MCHC. g/L	430.2 ± 16.4	440.5 ± 25.6	442.5 ± 17.9	448.5 ± 13.1	459 ± 22.5	647 ± 28.8	446.5 ± 16.5
RDWsd	19.1 ± 4.1	21.35 ± 1.65 *********	15.2 ± 1.02	16.6 ± 1.1	10.1 ± 0	11.5 ± 0.05	19.8 ± 4.65
RDWcv	18.8 ± 0.8	21.9 ± 1.11 *********	19.75 ± 0.35	18.4 ± 1.04	17.0 ± 1.05	31.2 ± 0.31	20.95 ± 2.05
PLT·10^9^/L	412.2 ± 24.4 **********	524.5 ± 18.9	554.5 ± 19.4	290.5 ± 21.4	340.2 ± 26	381 ± 19.6 **	561.2 ± 12.2
MPV	4.2 ± 0	3.8 ± 0.2	3.9 ± 0.4	3.3 ± 0	4.1 ± 0	3.5 ± 0	3.9 ± 0.3

* P7–8 = 0.007; ** P7–8 = 0.001; *** P7–8 = 0.0001; **** P7–8 = 0.0002; ***** P2–8 = 0.001; ****** P3–7 = 0.0002; ******* P3–7 = 0.001; ******** P5–7 = 0.0002; ********* P3–7 = 0.001; ********** P2–8 = 0.01.

**Table 5:** Maximum absorption bands and the extinction coefficients of the compounds **1a**–**1m** in UV spectra.

Compound	C (mol/L × 10^+5^)	λ_max_ (nm)	A_max_	ε (L·mol^−1^·cm^−1^ × 10^−5^)
**1a**	0.5106	194, 223	2.48, 0.95	4.9613, 1.8952
**1b**	0.5106	192, 223	1.88, 0.63	3.7602, 1.2547
**1c**	0.5106	192, 223	0.68, 0.21	1.3563, 0.4176
**1d**	0.5106	192, 223	1.39, 0.46	2.7759, 0.9229
**1e**	0.5106	194, 223	2.52, 0.97	5.0322, 1.9395
**1f**	0.5106	192, 223	0.71, 0.24	1.4108, 0.4867
**1g**	0.5106	192, 223	1.87, 0.65	3.7328, 1.2922
**1h**	0.5106	192, 223	1.17, 0.48	2.3389, 0.9636
**1i**	0.5106	194, 223	2.06, 0.82	4.1296, 1.6392
**1j**	0.5106	192, 223	0.70, 0.21	1.3974, 0.4279
**1k**	1.105	203, 257	2.92, 0.14	2.9165, 0.1374
**1l**	1.105	192, 223	1.99, 0.61	1.9926, 0.6054
**1m**	1.105	190, 223	1.18, 0.25	1.1791, 0.2485

## Data Availability

All Supplementary Materials can be found in the Supplementary Materials, or you can freely request them from the authors.

## Supplementary Materials

The following supporting information can be downloaded at: https://www.mdpi.com/article/10.3390/molecules30142967/s1, The original contributions presented in this study are included in the article/supplementary material. Further inquiries can be directed to the corresponding author. Figures S1–S26: ^1^H and ^13^C NMR spectra of obtained compounds; Figures S27–S39: mass spectra of obtained compounds; Figures S40 and S41: UV spectra of obtained compounds; Table S1: Diphenhydramine hydrochloride and synthesized ionic derivatives of diphenhydramine; Table S2: PASS prediction of hematopoietic activities for the studied compounds 1*HCl and **1a**–**1m**; Table S3: Hemogram parameters of peripheral blood.

## Abbreviations

The following abbreviations are used in this manuscript:

US	Ultrasonic
MW	Microwave
DM	Diabetes mellitus
WBC	White blood cell count
NEU	Neutrophil–lymphocyte ratio
LYM	Absolute lymphocyte count
MON	Monocyte count
EO	Eosinophil count
BAS	Basophils
RBC	Red blood cell count
HGB	Hemoglobin
HCT	Hematocrit
MCV	Mean corpuscular volume
MCH	Mean corpuscular hemoglobin
MCHC	Mean corpuscular hemoglobin concentration
RDW	Red blood cell distribution width
PLT	Total platelets volume
MPV	Mean platelet volume

## References

[1] Santos H.M., Lodeiro C., Capelo-Martinez J., Capelo-Martinez J.-L. (2009). Ultrasounds in Chemistry: Analytical Applications.

[2] Kappe C.O. (2004). Controlled Microwave Heating in Modern Organic Synthesis. Angew. Chem..

[3] Cravotto G., Boffa L., Mantegna S., Perego P., Avogadro M., Cintas P. (2008). Improved extraction of vegetable oils under high-intensity ultrasound and/or microwaves. Ultrason. Sonochem..

[4] Shah J.J., Mohanraj K. (2014). Comparison of Conventional and Microwave-assisted Synthesis of Benzotriazole Derivatives. Indian J. Pharm. Sci..

[5] Gedye R., Smith F., Westaway K., Ali H., Baldisera L., Laberge L., Rousell J. (1986). The use of microwave ovens for rapid organic synthesis. Tetrahedron Lett..

[6] Suslick K.S. (1990). Sonochemistry. Science.

[7] (2013). IDF Diabetes Atlas.

[8] Ahren B. (2005). New strategy in type 2 diabetes tested in clinical trials: Glucagon-like peptide 1 (GLP-1) affects basic caused of the disease. Lakartidningen.

[9] Ahren B., Pacini G., Foley J., Schweizer A. (2005). Improved meal-related (beta)-cell function and insulin sensitivity by the dipeptidyl peptidase-iv inhibitor vildagliptin in metformin-treated patients with type 2 diabetes over 1 year. Diabetes Care.

[10] Amori R.E., Lau J., Pittas A.G. (2007). Efficacy and safety of incretin therapy in type 2 diabetes: Systemic review and meta-analysis. JAMA.

[11] Srinivasan K., Ramarao P. (2007). Animal models in type 2 diabetes research: An overview. Indian J. Med. Res..

[12] Risbud M.V., Bhonde R.R. (2002). Models of pancreatic regeneration in diabetes. Diabetes Res. Clin. Pr..

[13] Leiter E.H., Prochazka M., Shultz L.D. (1987). Effect of immunodeficiency on diabetogenesis in getenocally diabetic (db/db) mice. J. Immunol..

[14] Pliszka M., Szablewski L. (2024). Associations between Diabetes Mellitus and Selected Cancers. Int. J. Mol. Sci..

[15] Rao Kondapally Seshasai S., Kaptoge S., Thompson A., Di Angelantonio E., Gao P., Sarwar N., Whincup P.H., Mukamal K.J., Gillum R.F., Holme I. (2011). Diabetes Mellitus, Fasting Glucose, and Risk of Cause-Specific Death. N. Engl. J. Med..

[16] Barone B.B., Yeh H.C., Snyder C.F., Peairs K.S., Stein K.B., Derr R.L., Wolff A.C., Brancati F.L. (2008). Long-Term All-Cause Mortality in Cancer Patients With Preexisting Diabetes Mellitus: A Systematic Review and Meta-Analysis. JAMA.

[17] Pavlidakey P.G., Brodell E.E., Helms S.E. (2009). Diphenhydramine as an alternative local anesthetic agent. J. Clin. Aesthet. Dermatol..

[18] Brambilla G., Mattioli F., Robbiano L., Martelli A. (2011). Genotoxicity and carcinogenicity studies of antihistamines. Arch. Toxicol..

[19] Giner B., Mergenbayeva S., Lomba L., Rafikova K., Dauletbakov A., Belyankova Y., Seilkhanov T., Zazybin A. (2020). Synthesis and Ecotoxicological Studies of ionic compounds based on Tolperisone, Diphenhydramine and Trimecaine. ChemistrySelect.

[20] Chayng P.J., Ain N., Ambia K., Noah R. (2020). Anti-diabetic activity of diphenhydramine in diabetic rats. Int. J. Res. Pharm. Sci..

[21] Telagari M., Hullatti K. (2015). In-vitro α-amylase and α-glucosidase inhibitory activity of Adiantum caudatum Linn. and Celosia argentea Linn. extracts and fractions. Indian J. Pharmacol..

[22] Haiwei R., Nana D., Xiaoqian N., Yonggang W., Wenguang F.N. (2021). Inhibitory effects of l-3-phenyllacitc acid on the activity of mushnroom pholyphenol oxidase. Food Sci. Technol..

[23] Bernfeld P., Colowick S.P., Kaplan N.O. (1955). Amylase, α and β. Methods in Enzymology.

[24] PASS Online Way2Drug Predictive Service. https://www.way2drug.com/passonline/.

[25] Dauletbakov A., Belyankova Y., Tursynbek S., Anapiayev B., Zolotareva D., Ten A., Zazybin A. (2022). Synthesis and growth-stimulating activity of trimecaine ethyl iodoethanoate. Chem. J. Kaz..

[26] Giemsa G. (1904). Eine Vereinfachung und Vervollkommnung meiner Methylenazur-Methylenblau-Eosin-Färbemethode zur Erzielung der Romanowsky-Nochtschen Chromatinfärbung. Cent. Bakt..

[27] Order of the Minister of Healthcare of the Republic of Kazakhstan No. KR DSM-181/2020 of 4 November 2020 On approval of the Rules for the Assessment of Materials and Compliance of Preclinical (non-clinical) Studies with Good Laboratory Practice (GLP) Requirements of the Republic of Kazakhstan and/or the Eurasian Economic Union within the Framework of Pharmaceutical Inspection. (Registered with the Ministry of Justice of the Republic of Kazakhstan on 5 November 2020 under No. 21596). https://adilet.zan.kz/eng/docs/V2000021596.

[28] RCSB Protein Data Bank (RCSB PDB). https://www.rcsb.org/.

