# Supplementary material for: Ultrasound and Microwave-Assisted Synthesis and Antidiabetic and Hematopoietic Activity of Diphenhydramine Derivatives
Source: Molecules. 2025 Jul 15;30(14):2967. doi: 10.3390/molecules30142967 (PMC12299238; doi:10.3390/molecules30142967)
Supplement: Supplementary file 1 [file molecules-30-02967-s001.zip › molecules-3647216-supplementary.pdf]

Supplementary materials

Ultrasound and Microwave-Assisted Synthesis and Antidiabetic and Hematopoietic Activity of Diphenhydramine Derivatives

Anuar Dauletbakov [1], Yelizaveta Belyankova [1], Saniya Assylbekova [1], Darya Zolotareva [1], Sarah Bayazit [1], Layilya Baktybayeva [2], Ulan Kemelbekov [3], Valentina Yu [4], Nailya Ibragimova [5] and Alexey Zazybin [1,*]

**Figure S1**. ^{1}H NMR spectrum of compound **1a**	2
**Figure S2**. ^{13}C NMR spectrum of compound **1a**	3
**Figure S3**. ^{1}H NMR spectrum of compound **1b**	4
**Figure S4**. ^{13}C NMR spectrum of compound **1b**	5
**Figure S5**. ^{1}H NMR spectrum of compound **1c**	6
**Figure S6**. ^{13}C NMR spectrum of compound **1c**	7
**Figure S7**. ^{1}H NMR spectrum of compound **1d**	8
**Figure S8**. ^{13}C NMR spectrum of compound **1d**	9
**Figure S9**. ^{1}H NMR spectrum of compound **1e**	10
**Figure S10**. ^{13}C NMR spectrum of compound **1e**	11
**Figure S11**. ^{1}H NMR spectrum of compound **1f**	12
**Figure S12**. ^{13}C NMR spectrum of compound **1f**	13
**Figure S13**. ^{1}H NMR spectrum of compound **1g**	14
**Figure S14**. ^{13}C NMR spectrum of compound **1g**	15
**Figure S15**. ^{1}H NMR spectrum of compound **1h**	16
**Figure S16**. ^{13}C NMR spectrum of compound **1h**	17
**Figure S17**. ^{1}H NMR spectrum of compound **1i**	18
**Figure S18**. ^{13}C NMR spectrum of compound **1i**	19
**Figure S19**. ^{1}H NMR spectrum of compound **1j**	20
**Figure S20**. ^{13}C NMR spectrum of compound **1j**	21
**Figure S21**. ^{1}H NMR spectrum of compound **1k**	22
**Figure S22**. ^{13}C NMR spectrum of compound **1k**	23
**Figure S23**. ^{1}H NMR spectrum of compound **1l**	24
**Figure S24**. ^{13}C NMR spectrum of compound **1l**	25
**Figure S25**. ^{1}H NMR spectrum of compound **1m**	26
**Figure S26**. ^{13}C NMR spectrum of compound**1m**	27
**Figure S27.** Mass-spectrum of compound **1a**	28
**Figure S28.** Mass-spectrum of compound **1b**	29
**Figure S29.** Mass-spectrum of compound **1c**	30
**Figure S30.** Mass-spectrum of compound **1d**	31
**Figure S31.** Mass-spectrum of compound **1e**	32
**Figure S32.** Mass-spectrum of compound **1f**	33
**Figure S33.** Mass-spectrum of compound **1g**	34
**Figure S34.** Mass-spectrum of compound **1h**	35
**Figure S35.** Mass-spectrum of compound **1i**	36
**Figure S36.** Mass-spectrum of compound **1j**	37
**Figure S37.** Mass-spectrum of compound **1k**	38
**Figure S38.** Mass-spectrum of compound **1l**	39
**Figure S39.** Mass-spectrum of compound **1m**	40
**Figure S40.** UV spectra of compounds **1a-1g**	41
**Figure S41.** UV spectra of compounds **1h-1m**	42
**Table S1**. Diphenhydramine hydrochloride and synthesized ionic derivatives of diphenhydramine.	43
**Table S2.** PASS prediction of hematopoietic activities for the studied compounds 1*HCl and **1a-1m**	45
**Table S3.** Hemogram parameters of peripheral blood	46

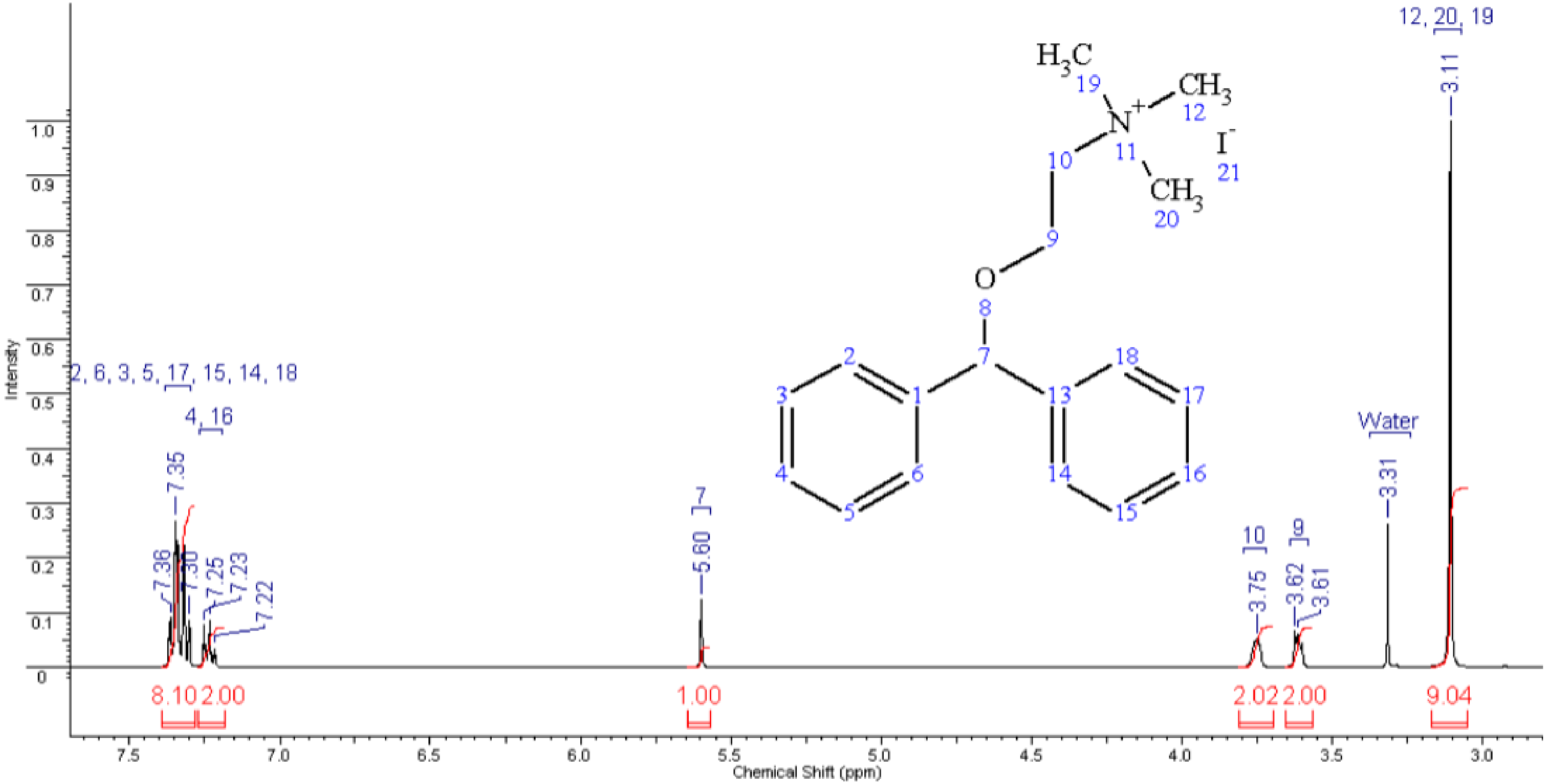

Figure S1. ^{1}H NMR spectrum (DMSO) of compound **1a**

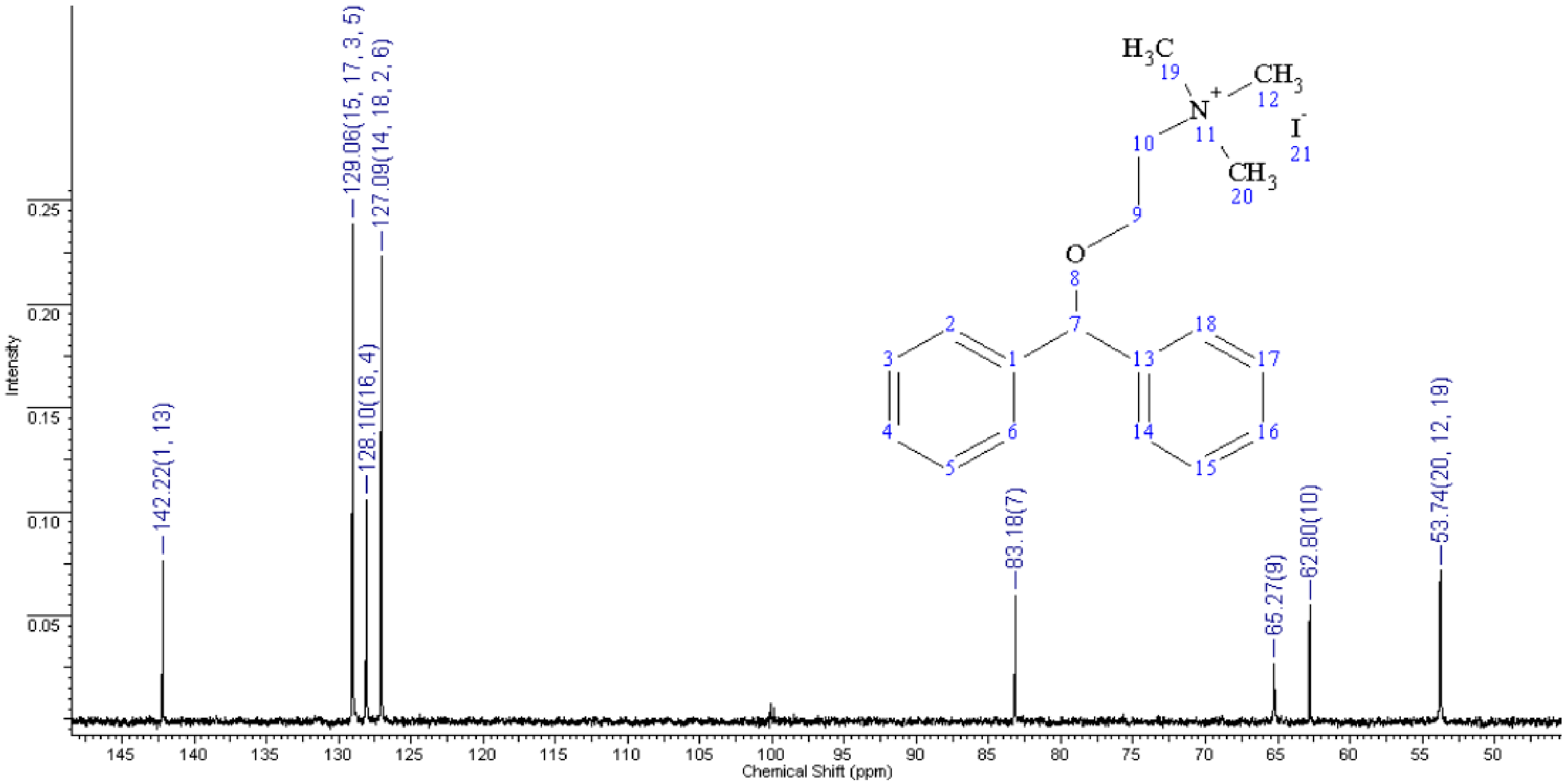

Figure S2. ^{13}C NMR spectrum (DMSO) of compound **1a**

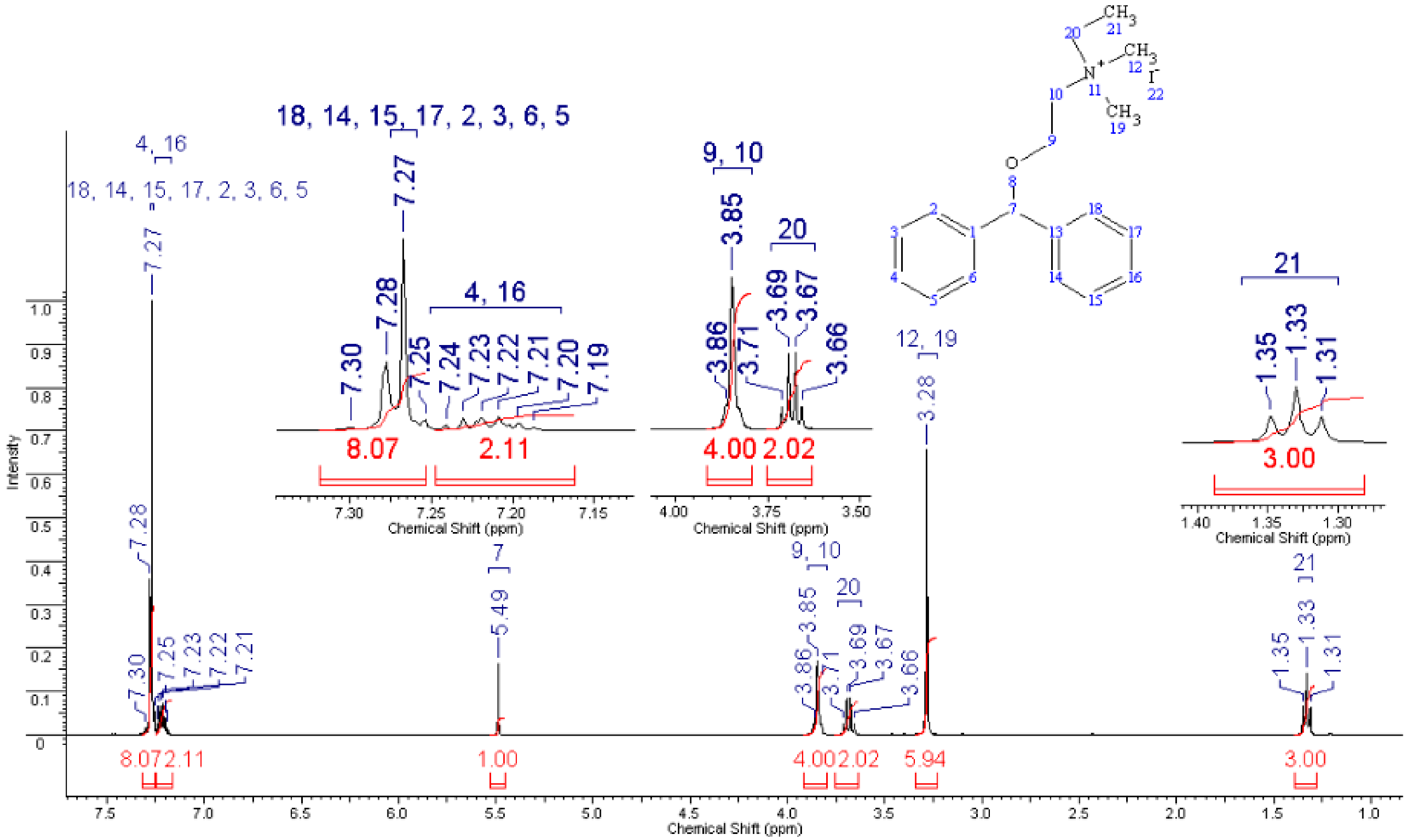

Figure S3. ^{1}H NMR spectrum (DMSO) of compound **1b**

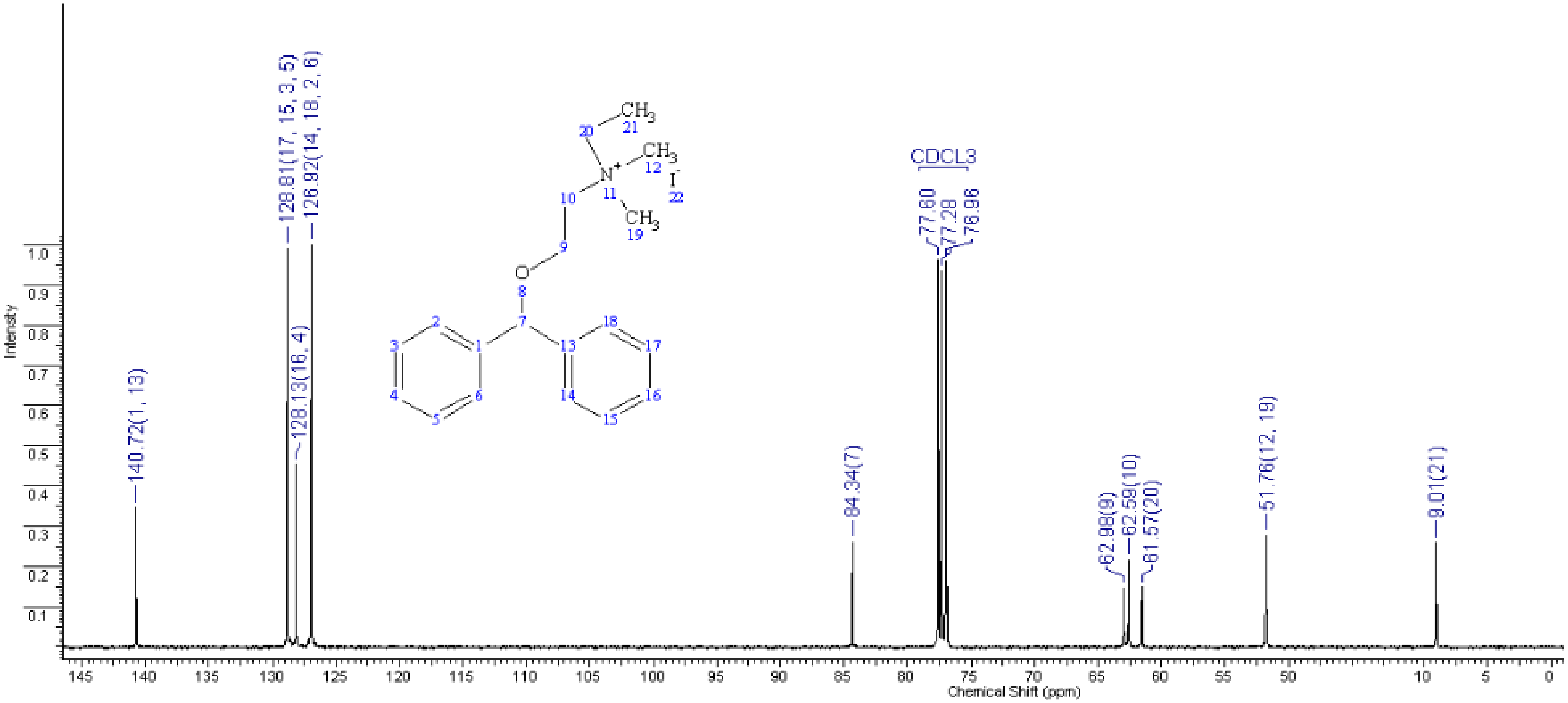

Figure S4. ^{13}C NMR spectrum (DMSO) of compound **1b**

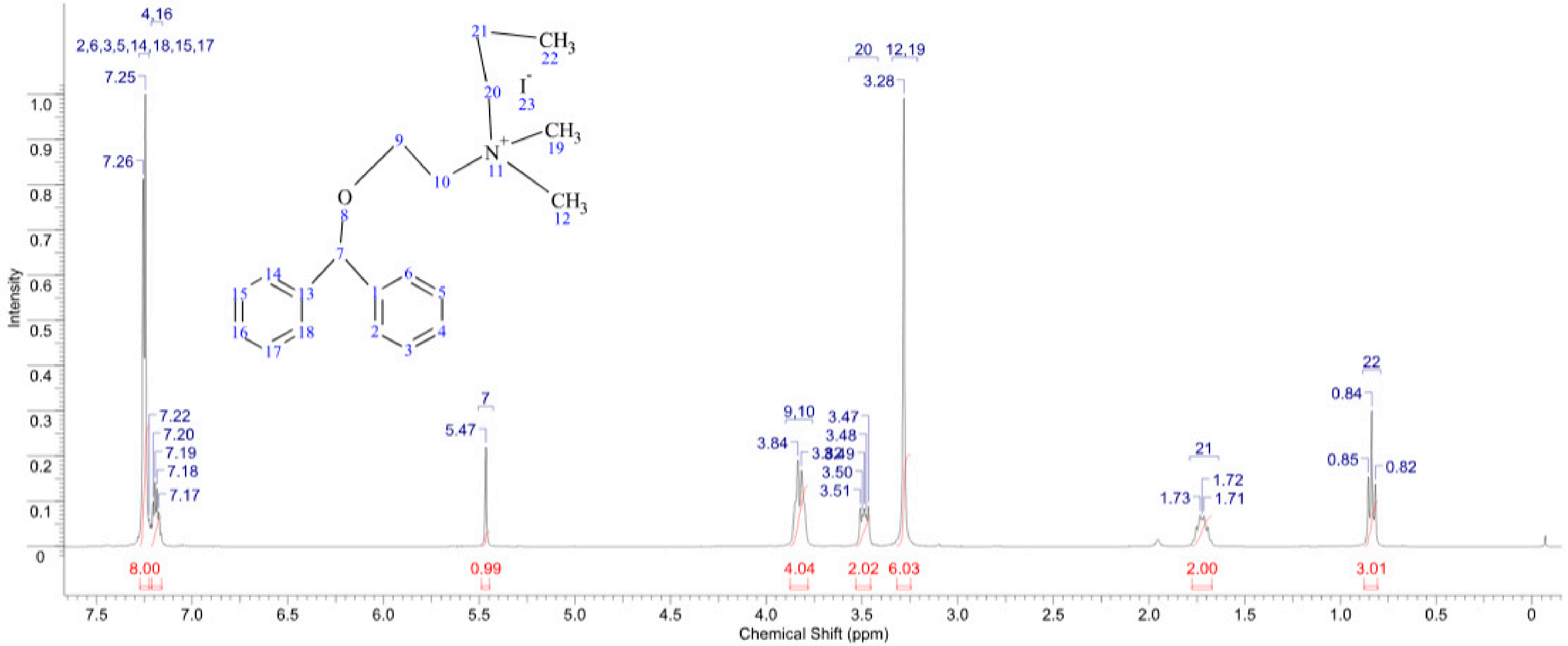

Figure S5. ^{1}H NMR spectrum ($CDCl_3$) of compound **1c**

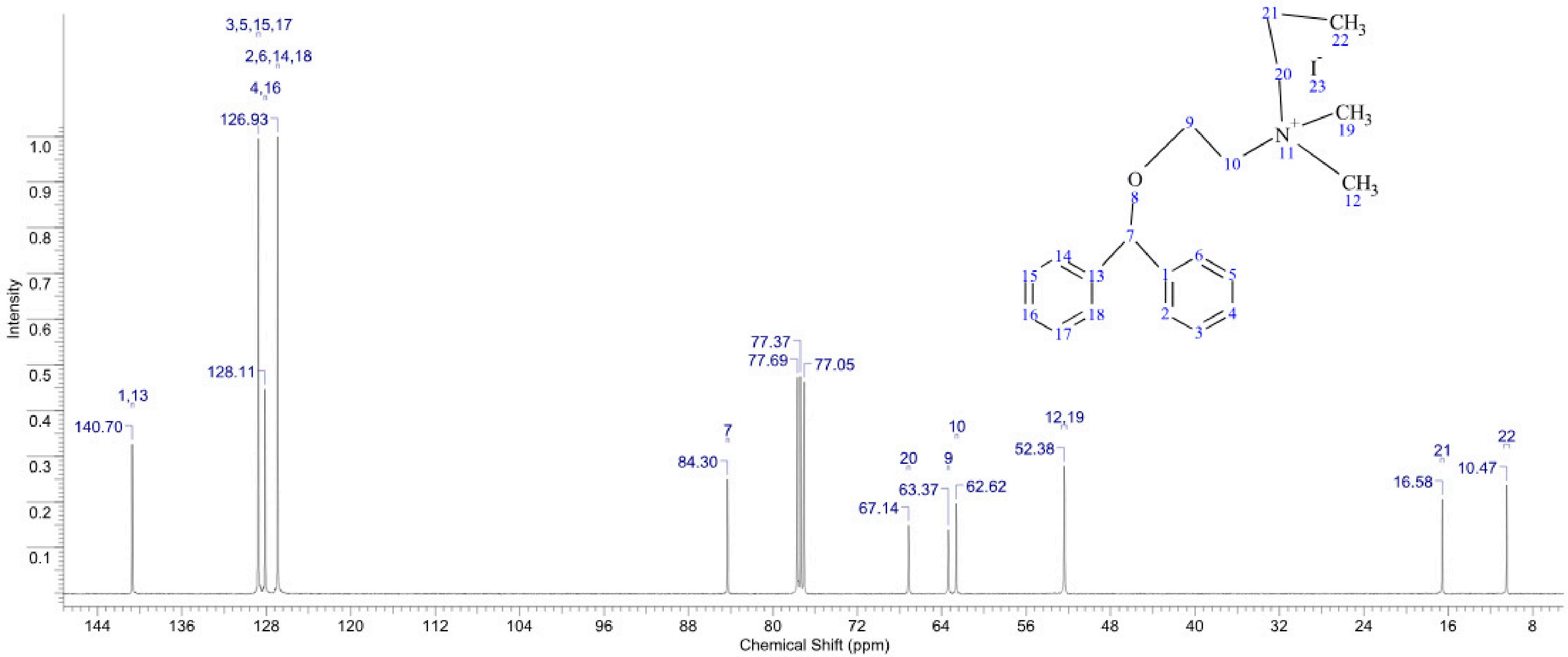

Figure S6. ^{13}C NMR spectrum ($CDCl_3$) of compound **1c**

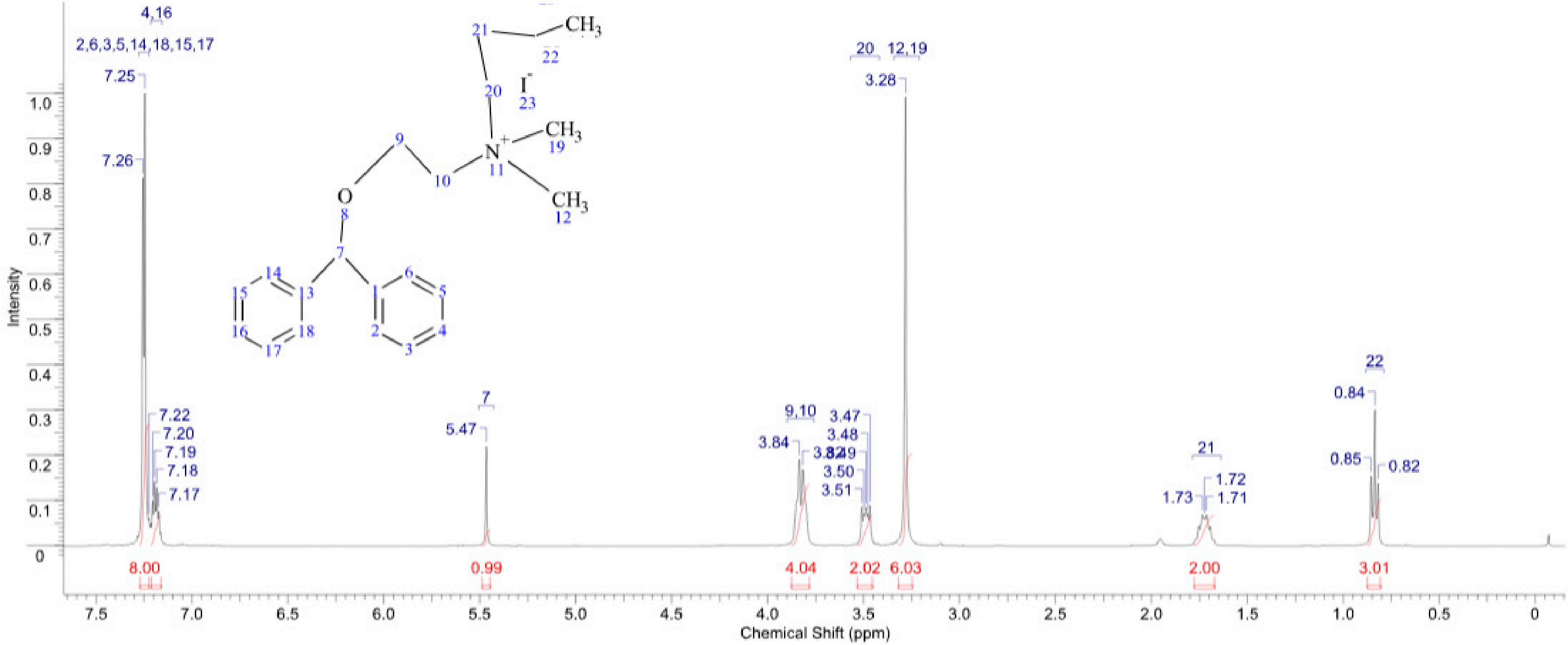

Figure S7. ^{1}H NMR spectrum ($CDCl_3$) of compound **1d**

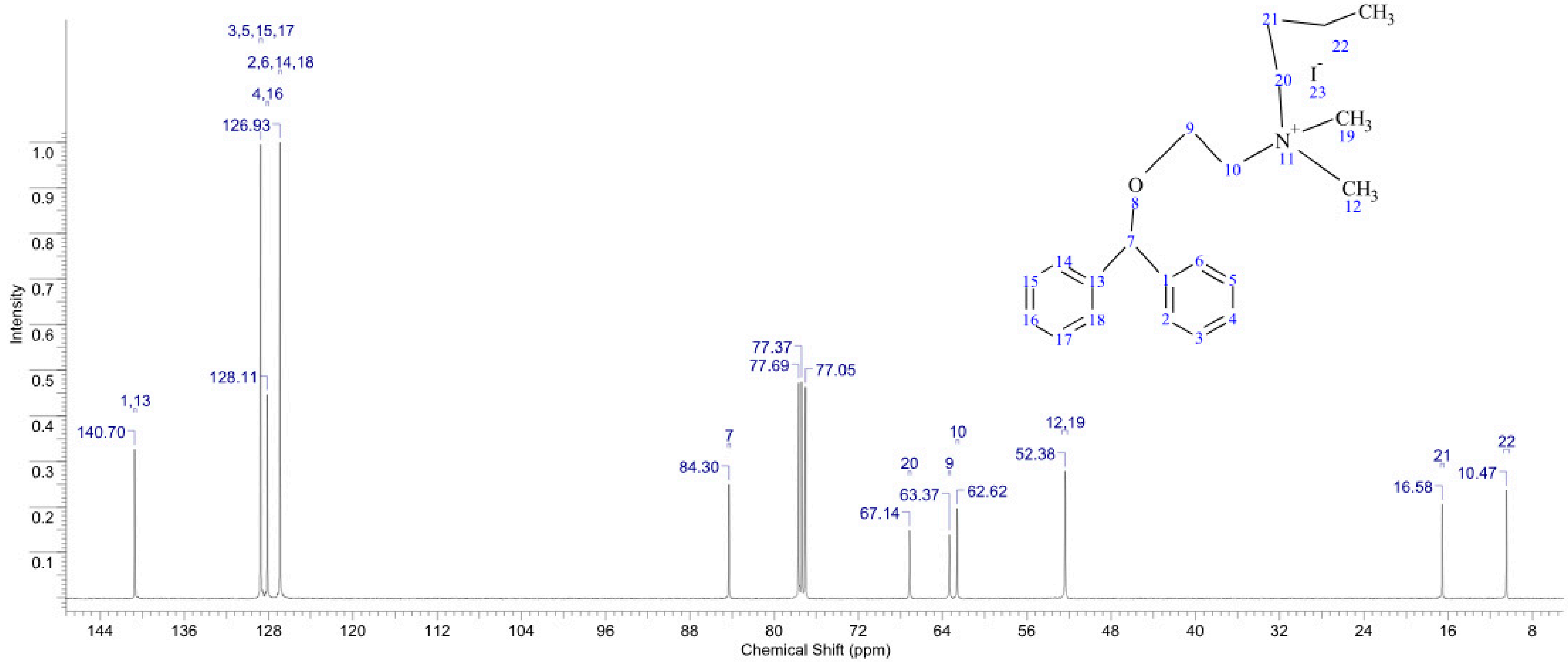

Figure S8. ^{13}C NMR spectrum ($CDCl_3$) of compound **1d**

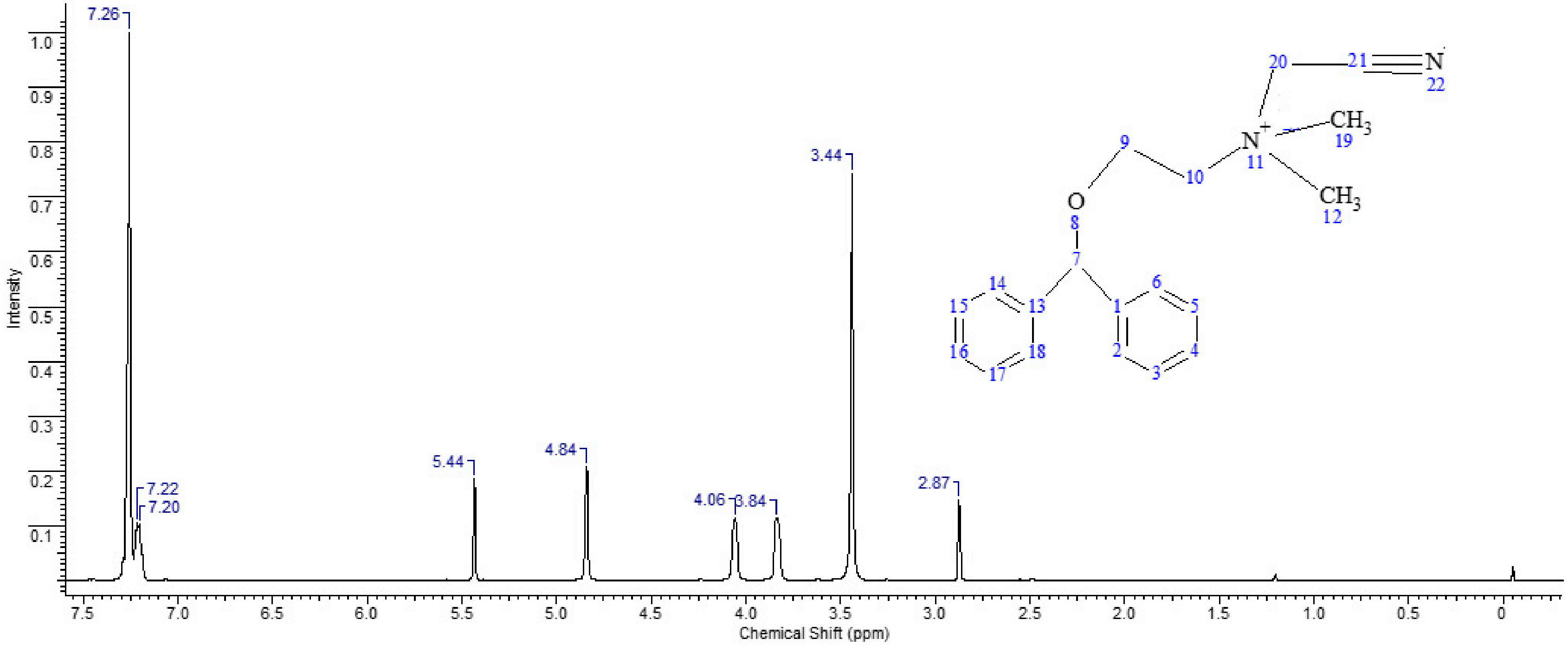

Figure S9. ^{1}H NMR spectrum ($CDCl_3$) of compound **1e**

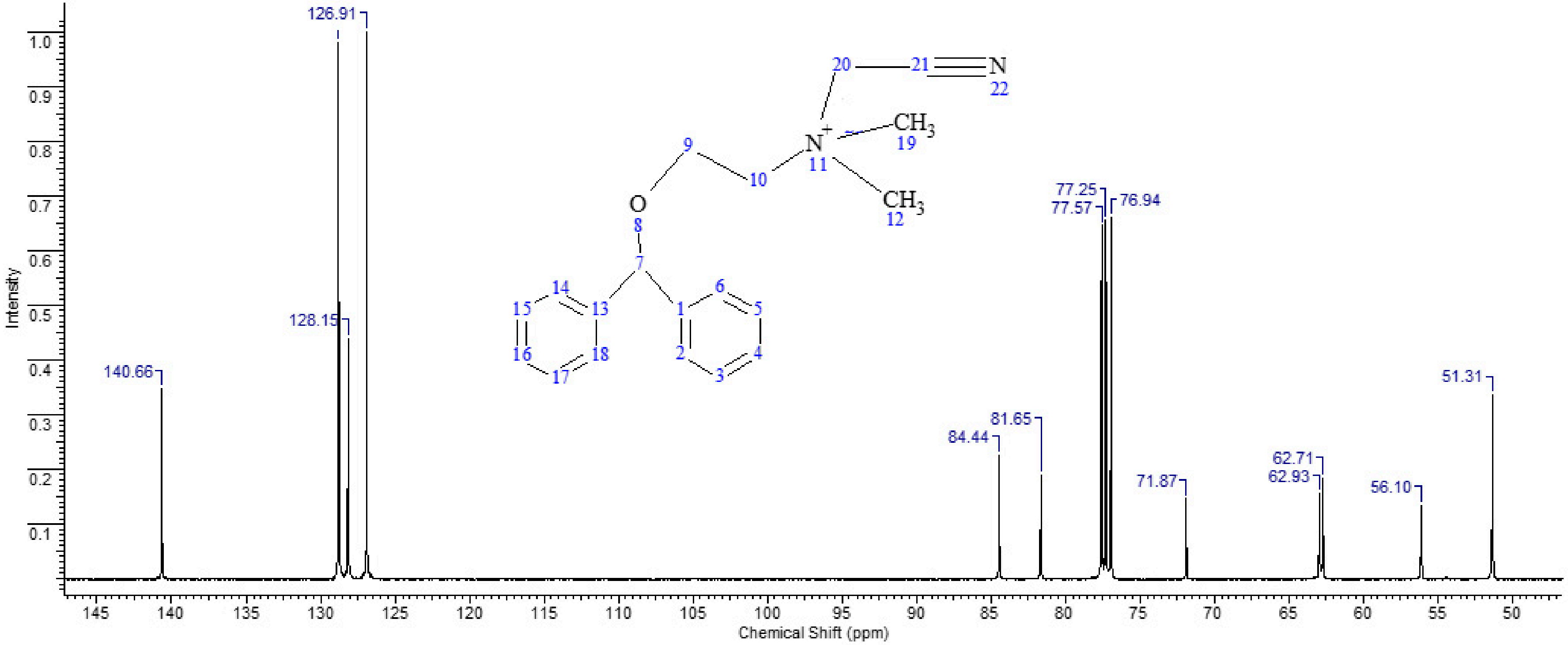

Figure S10. ^{13}C NMR spectrum ($CDCl_3$) of compound **1e**

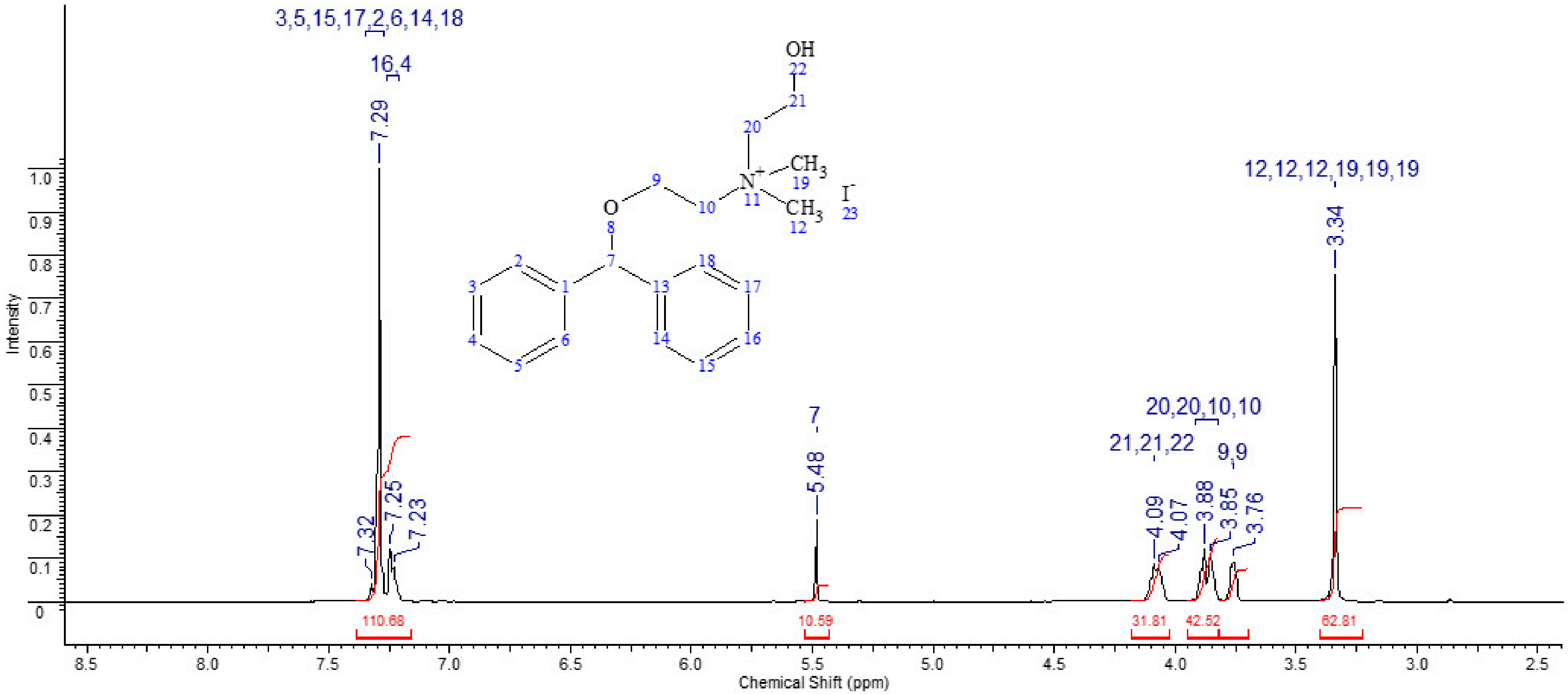

Figure S11. ^{1}H NMR spectrum ($CDCl_3$) of compound **1f**

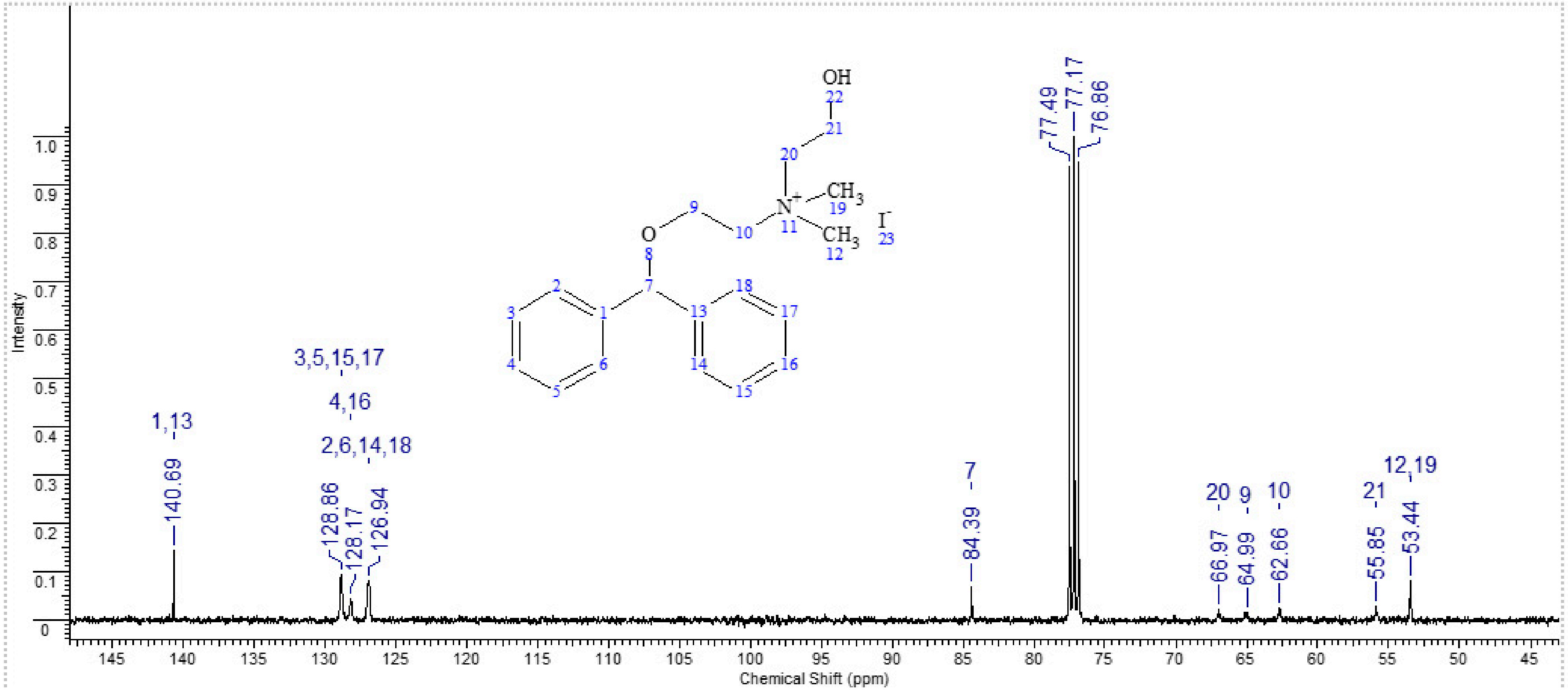

Figure S12. ^{13}C NMR spectrum ($CDCl_3$) of compound **1f**

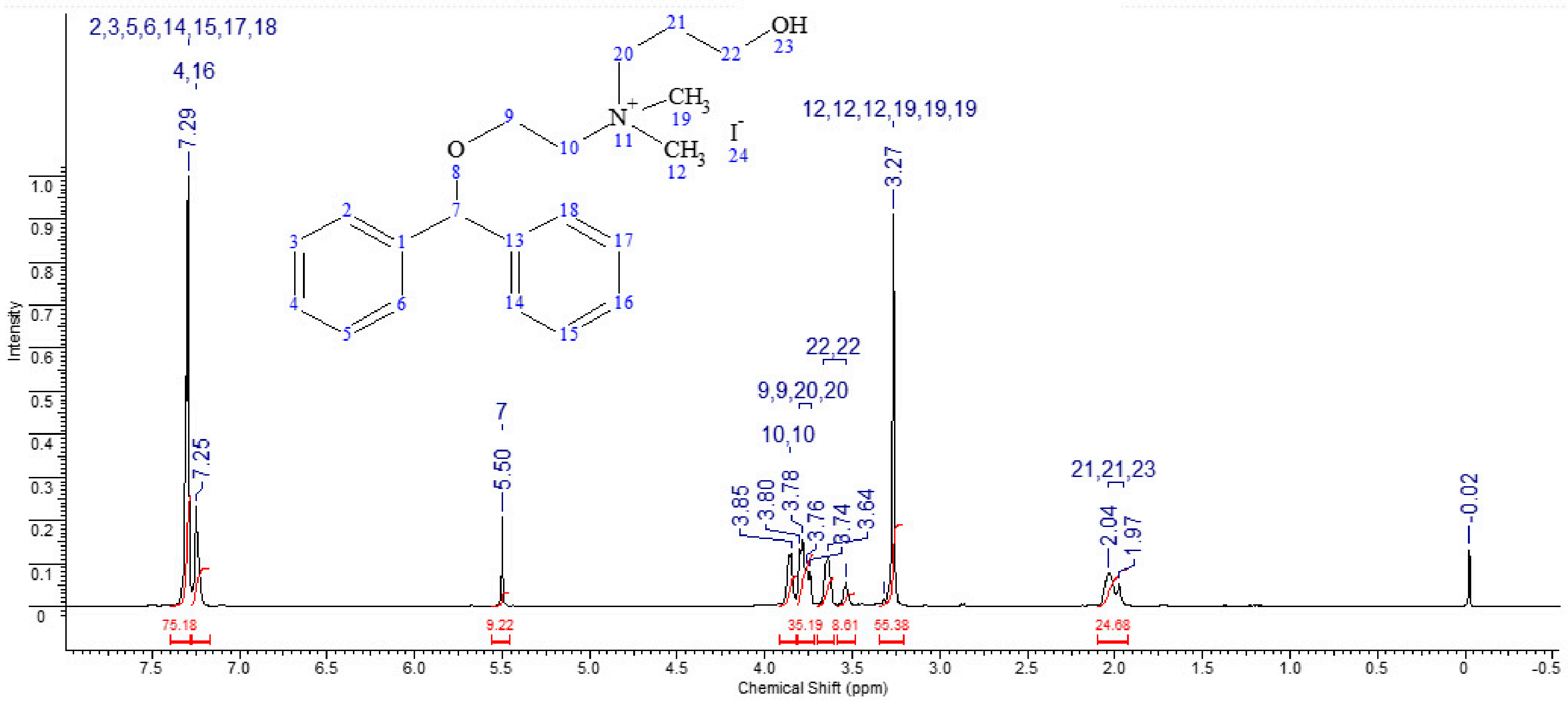

Figure S13. ^{1}H NMR spectrum ($CDCl_3$) of compound **1g**

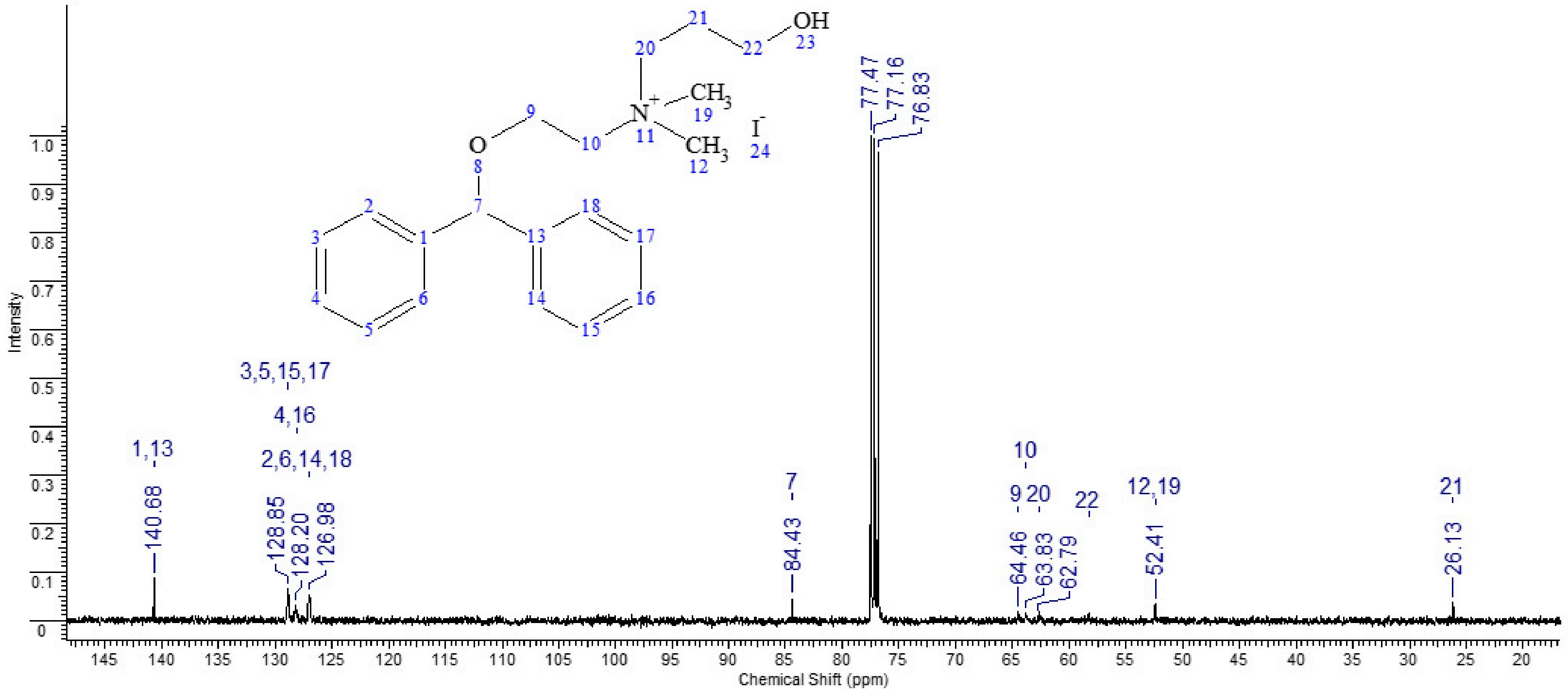

Figure S14. ^{13}C NMR spectrum ($CDCl_3$) of compound **1g**

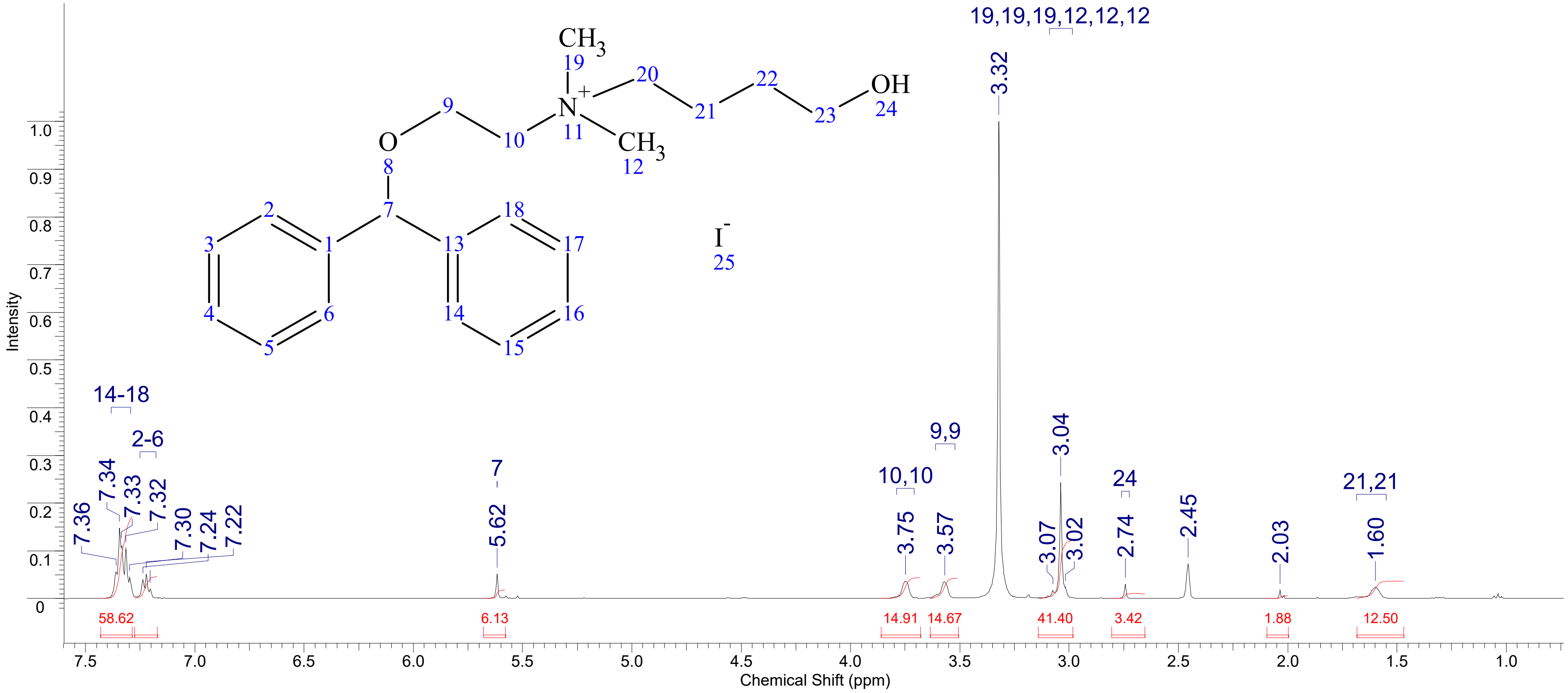

Figure S15. ^1H NMR spectrum (DMSO-d_6) of compound **1h**

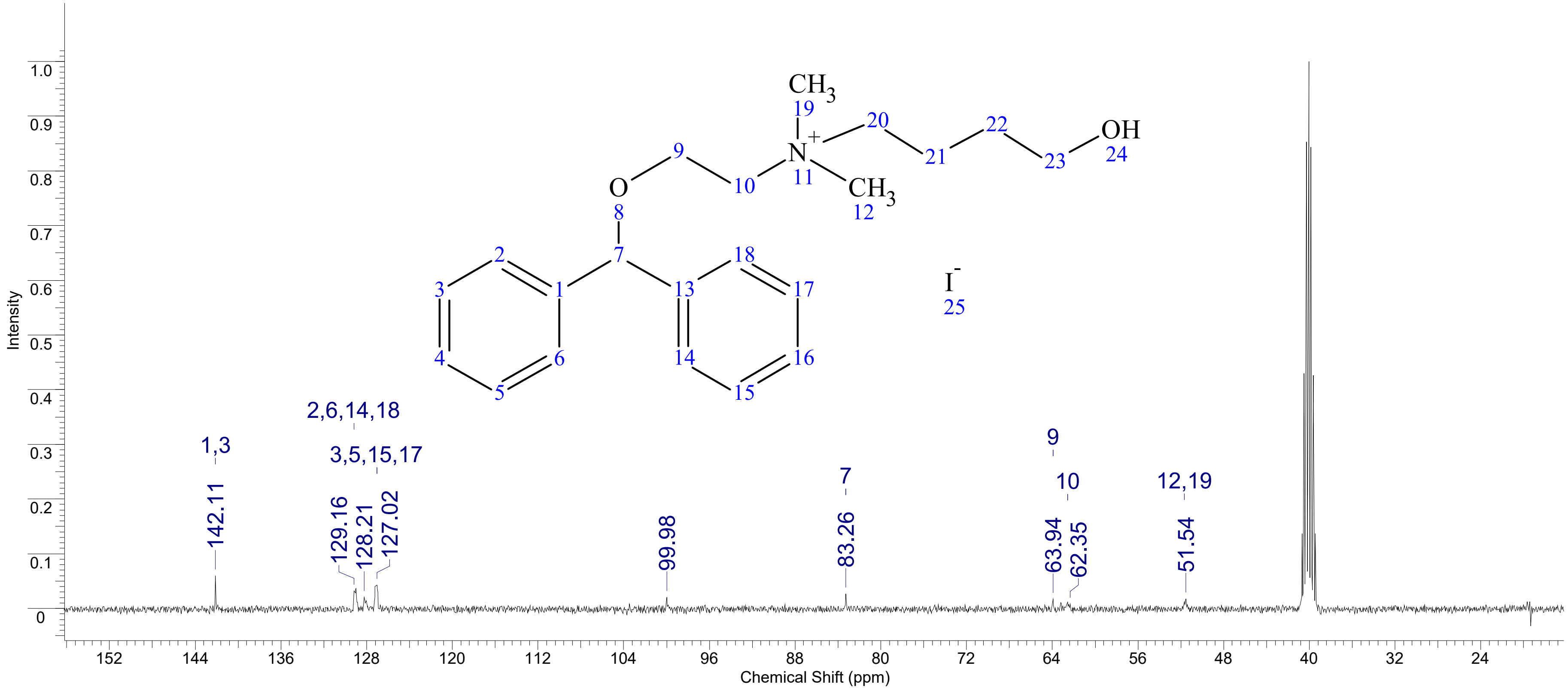

Figure S16. ^{13}C NMR spectrum (DMSO-d_6) of compound **1h**

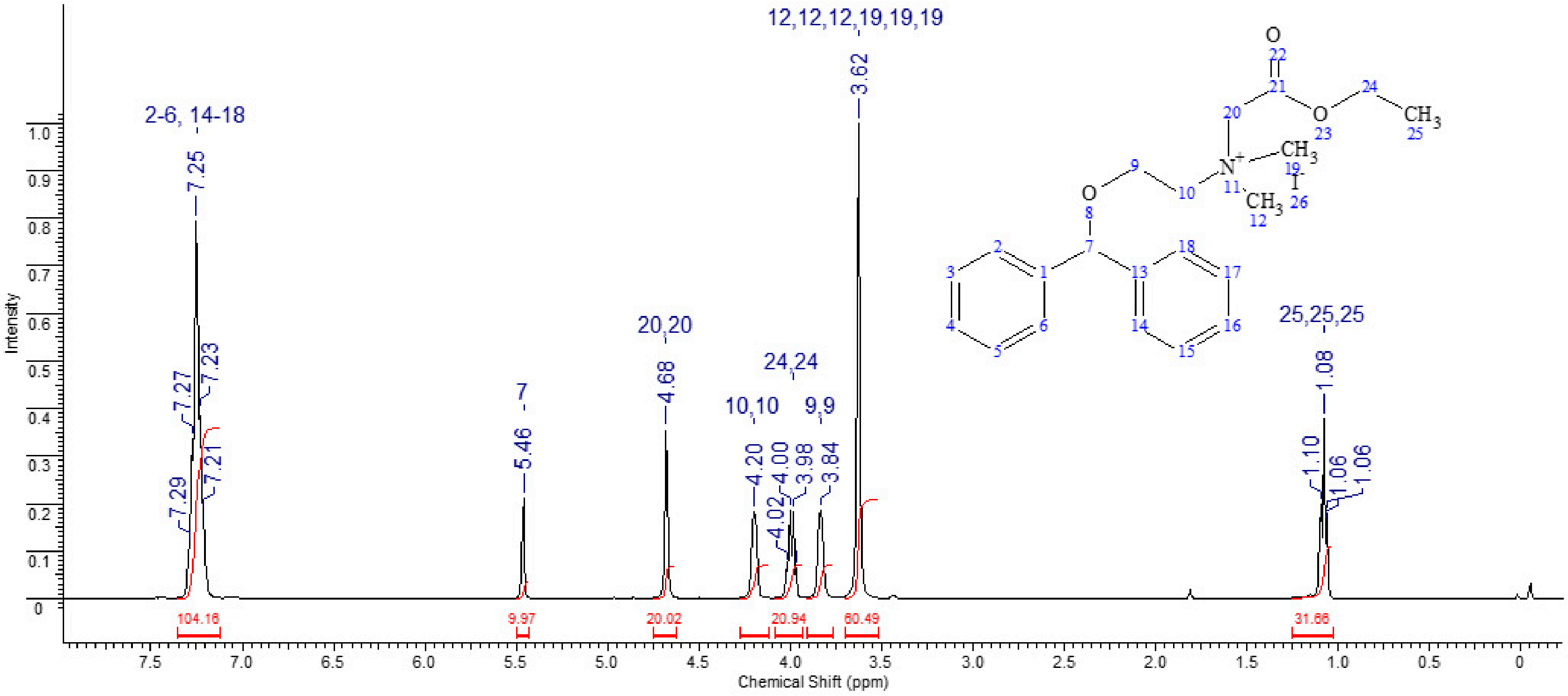

Figure S17. ^{1}H NMR spectrum ($CDCl_3$) of compound **1i**

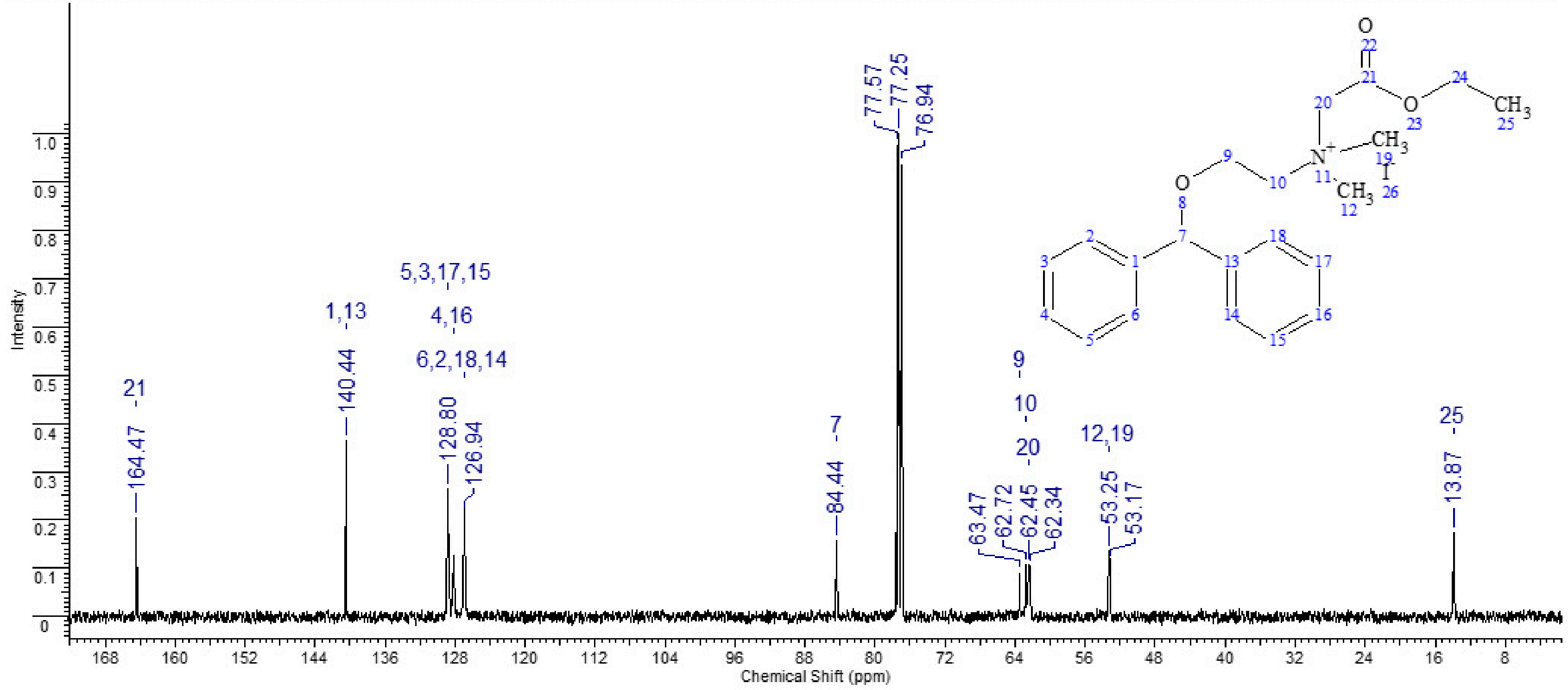

Figure S18. ^{13}C NMR spectrum ($CDCl_3$) of compound **1i**

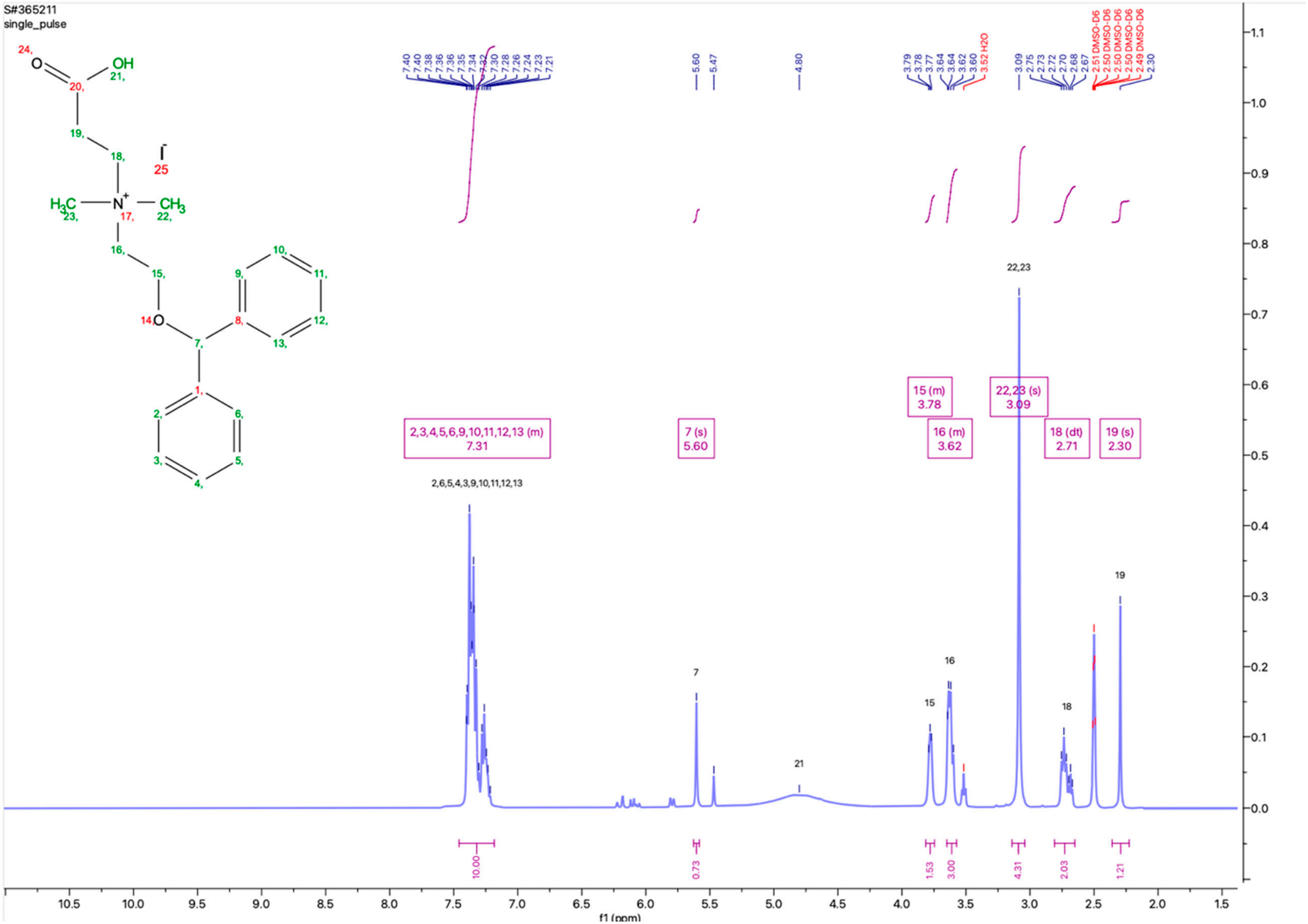

Figure S19. ^{1}H NMR spectrum (DMSO-d_6) of compound **1j**

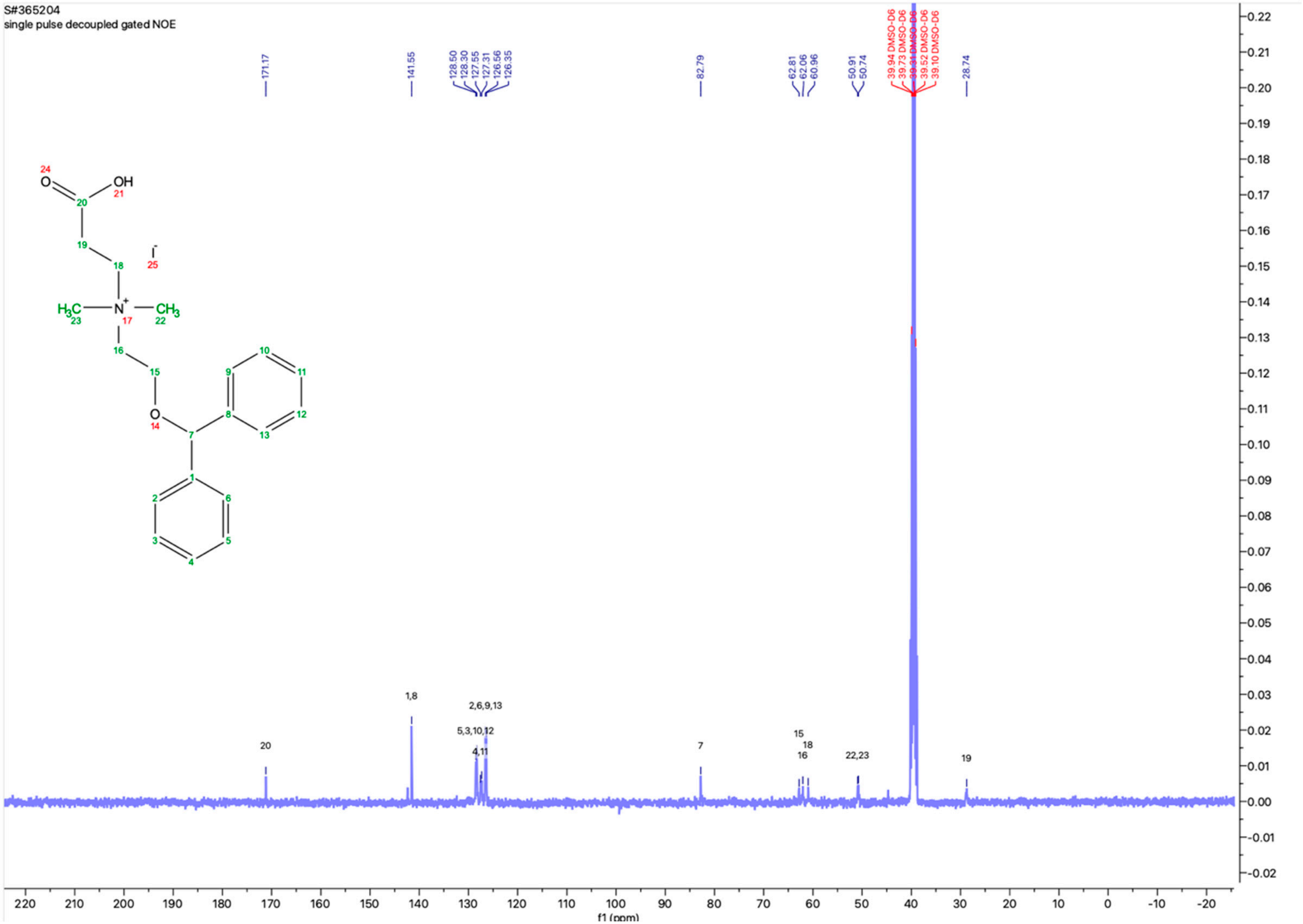

Figure S20. ${}^{13}C$ NMR spectrum (DMSO-d_6) of compound **1j**

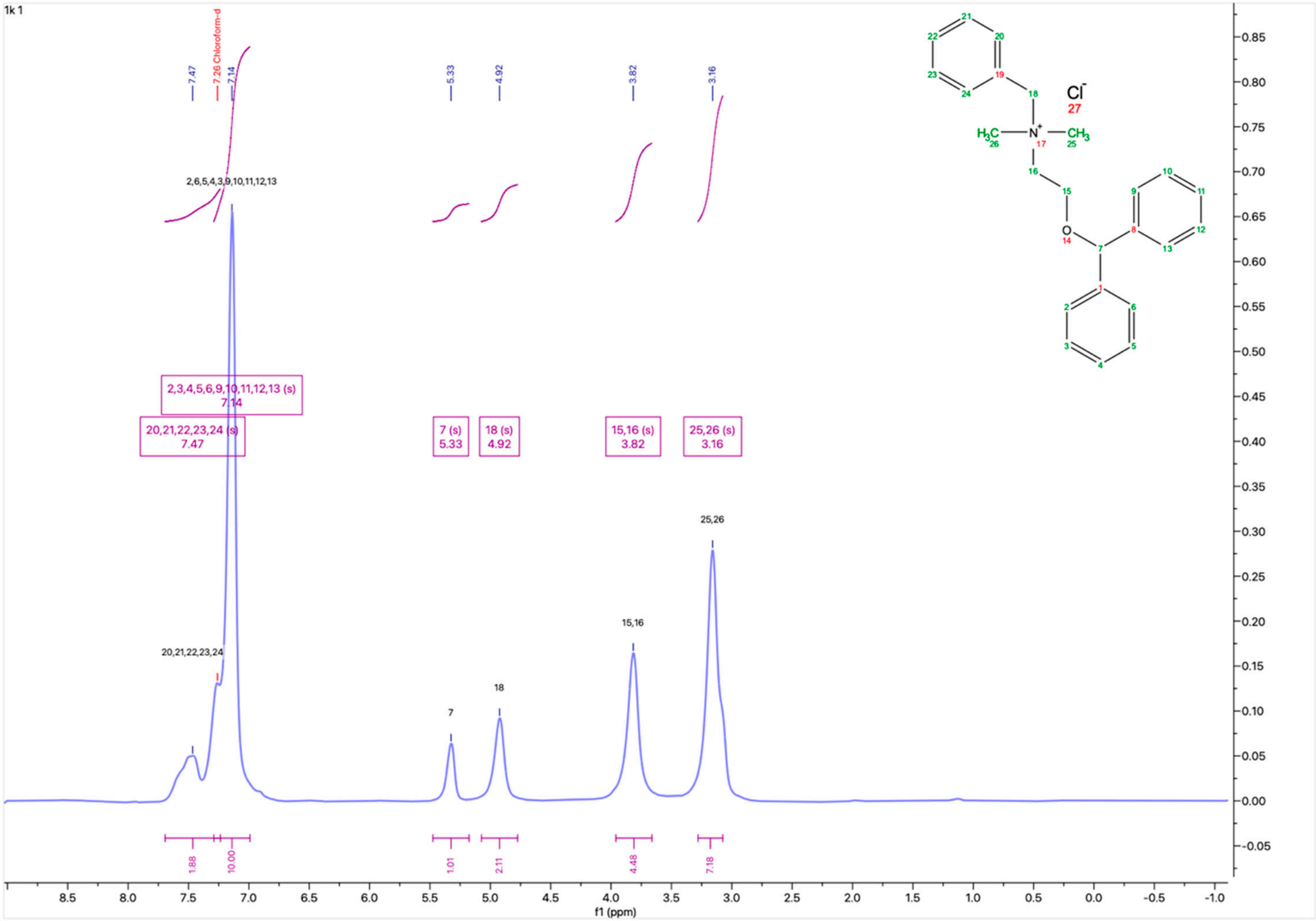

Figure S21. ^{1}H NMR spectrum ($CDCl_3$) of compound **1k**

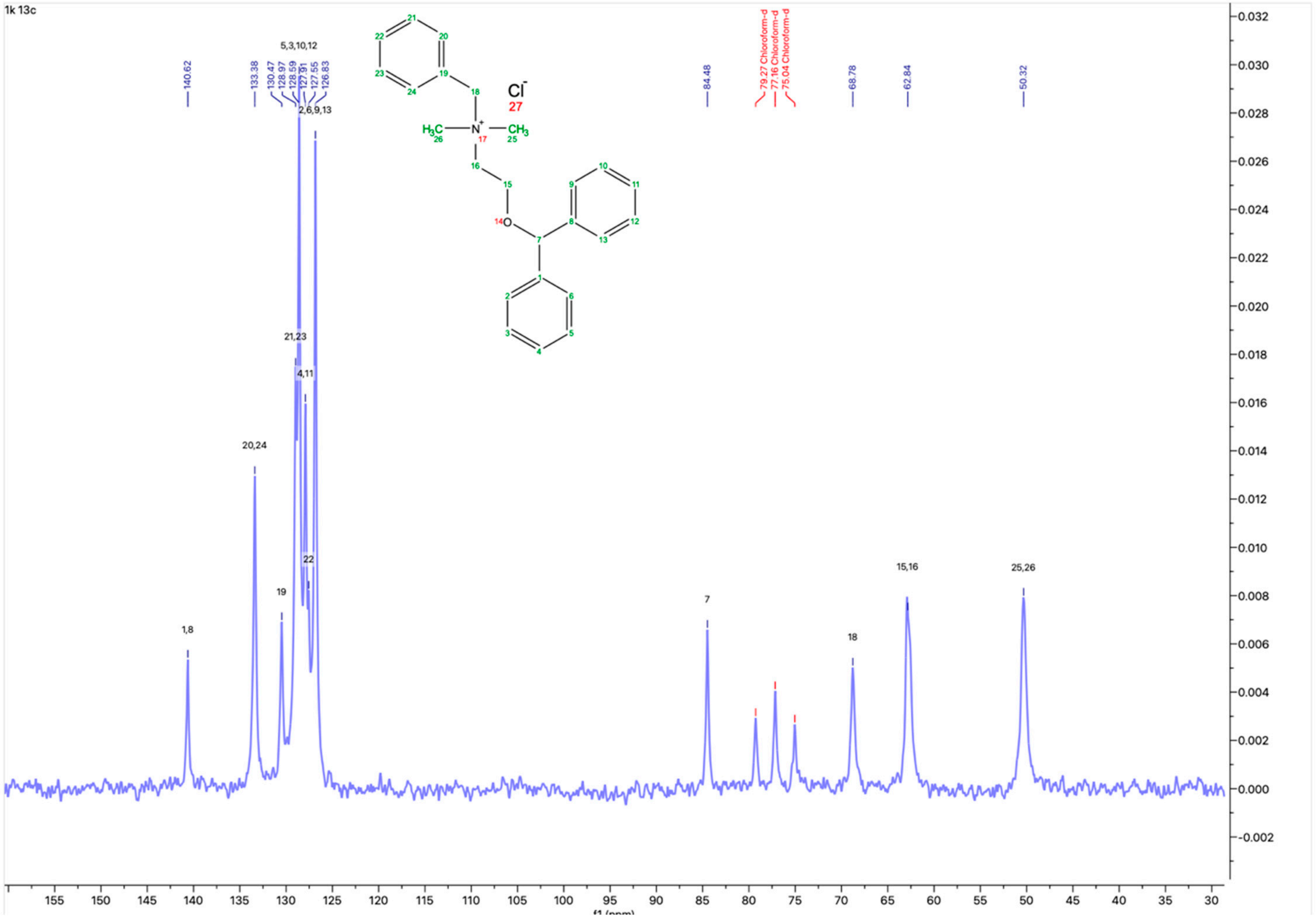

Figure S22. ^{13}C NMR spectrum ($CDCl_3$) of compound **1k**

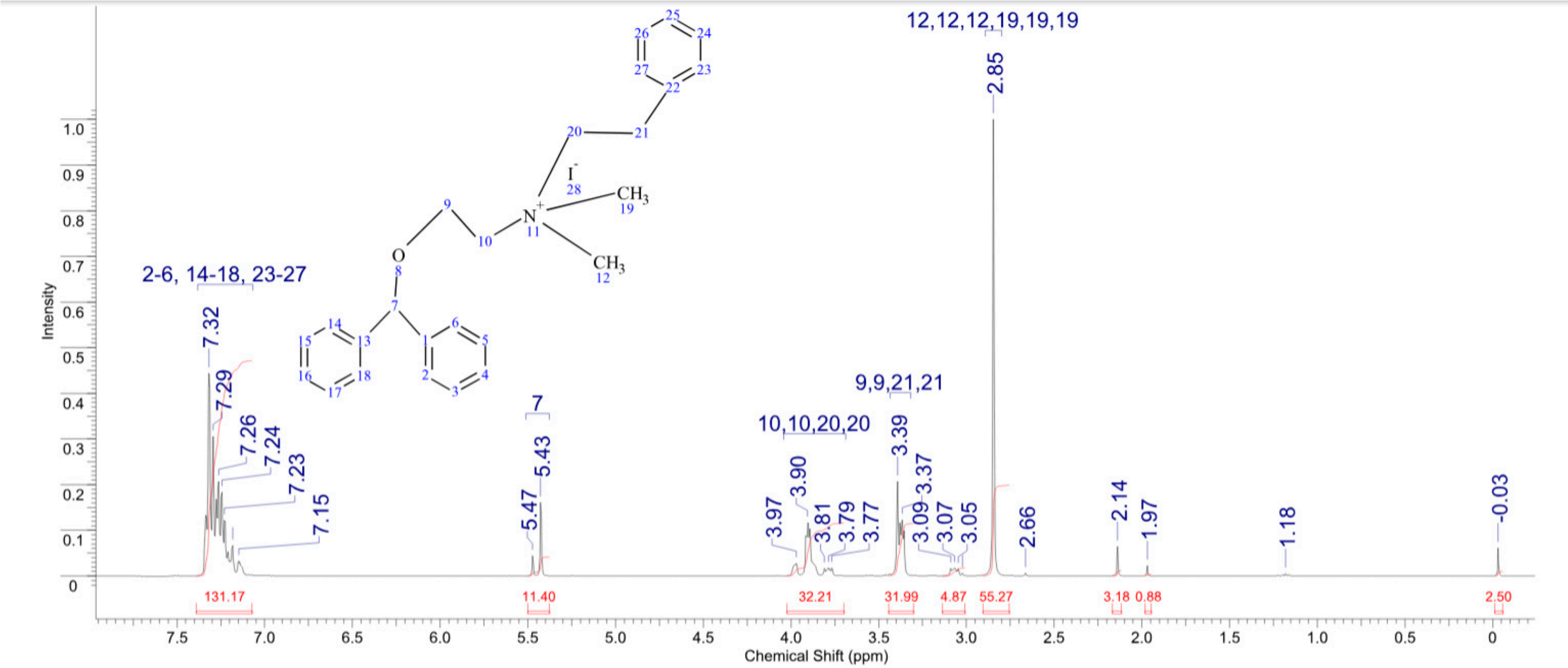

Figure S23. ^{1}H NMR spectrum ($CDCl_3$) of compound **1l**

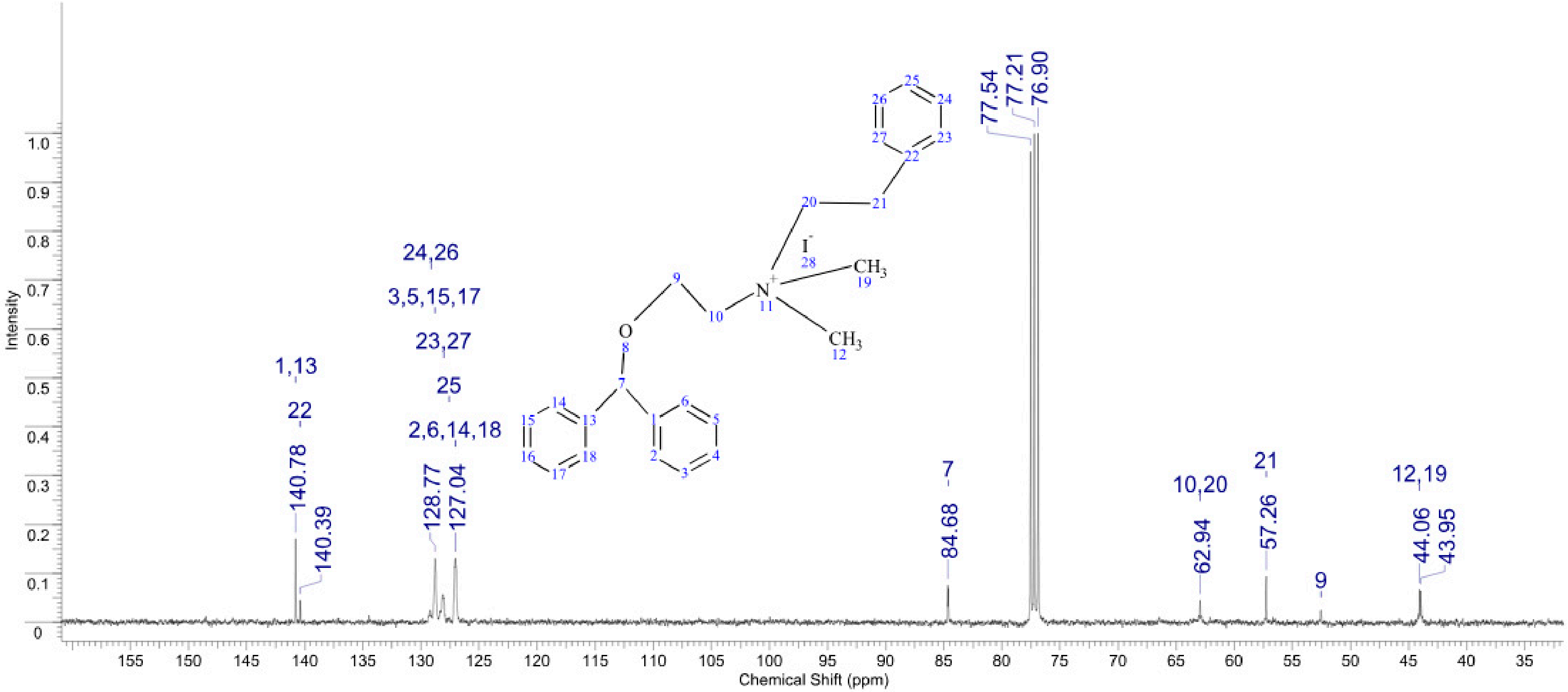

Figure S24. ^{13}C NMR spectrum ($CDCl_3$) of compound **1l**

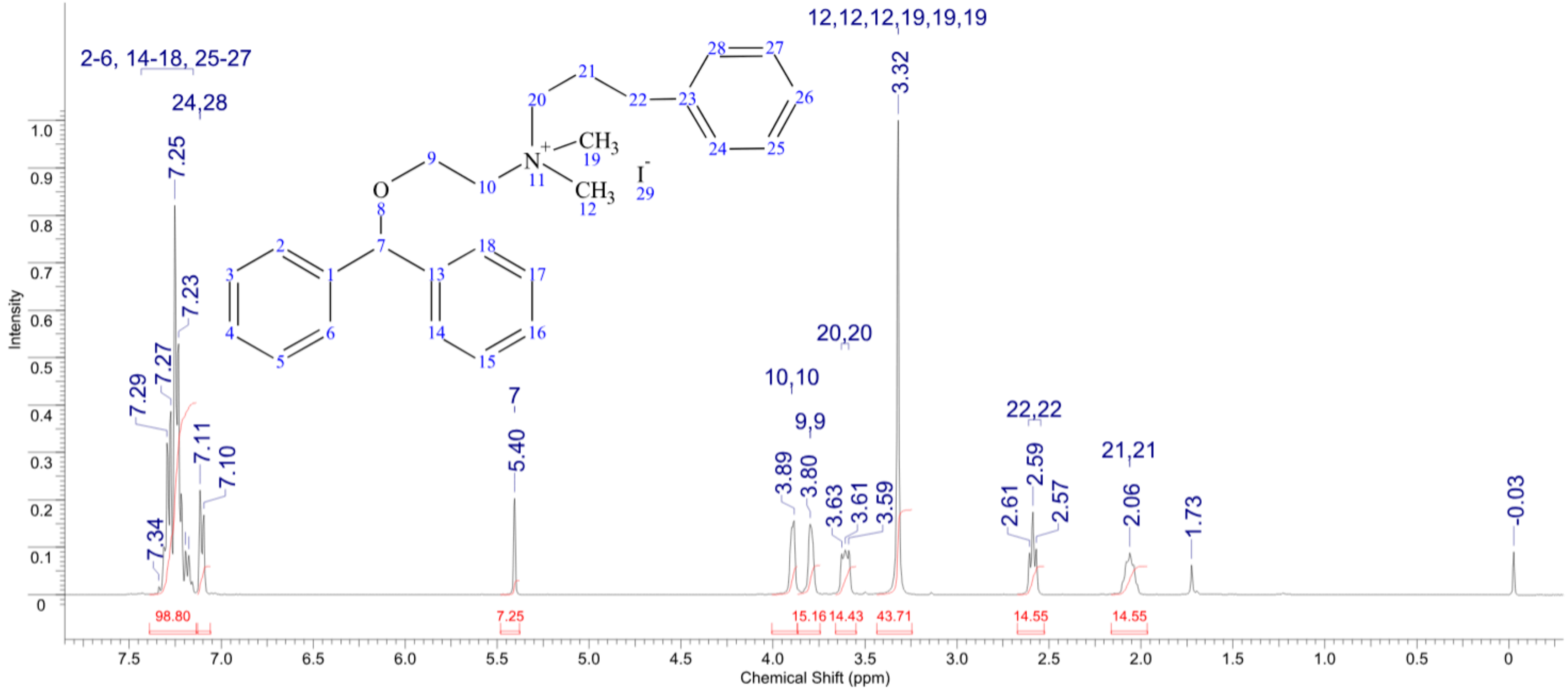

Figure S25. ^{1}H NMR spectrum ($CDCl_3$) of compound **1m**

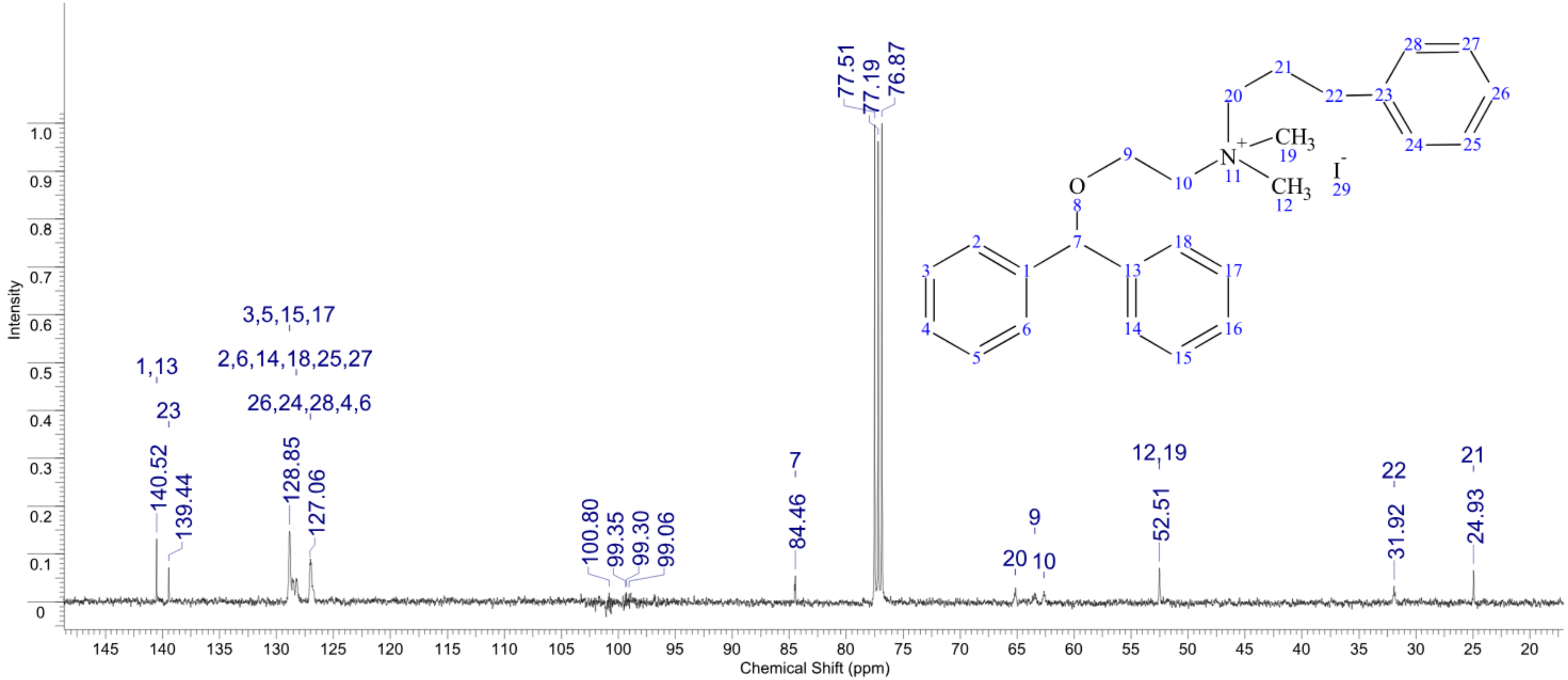

Figure S26. ^{13}C NMR spectrum ($CDCl_3$) of compound **1m**

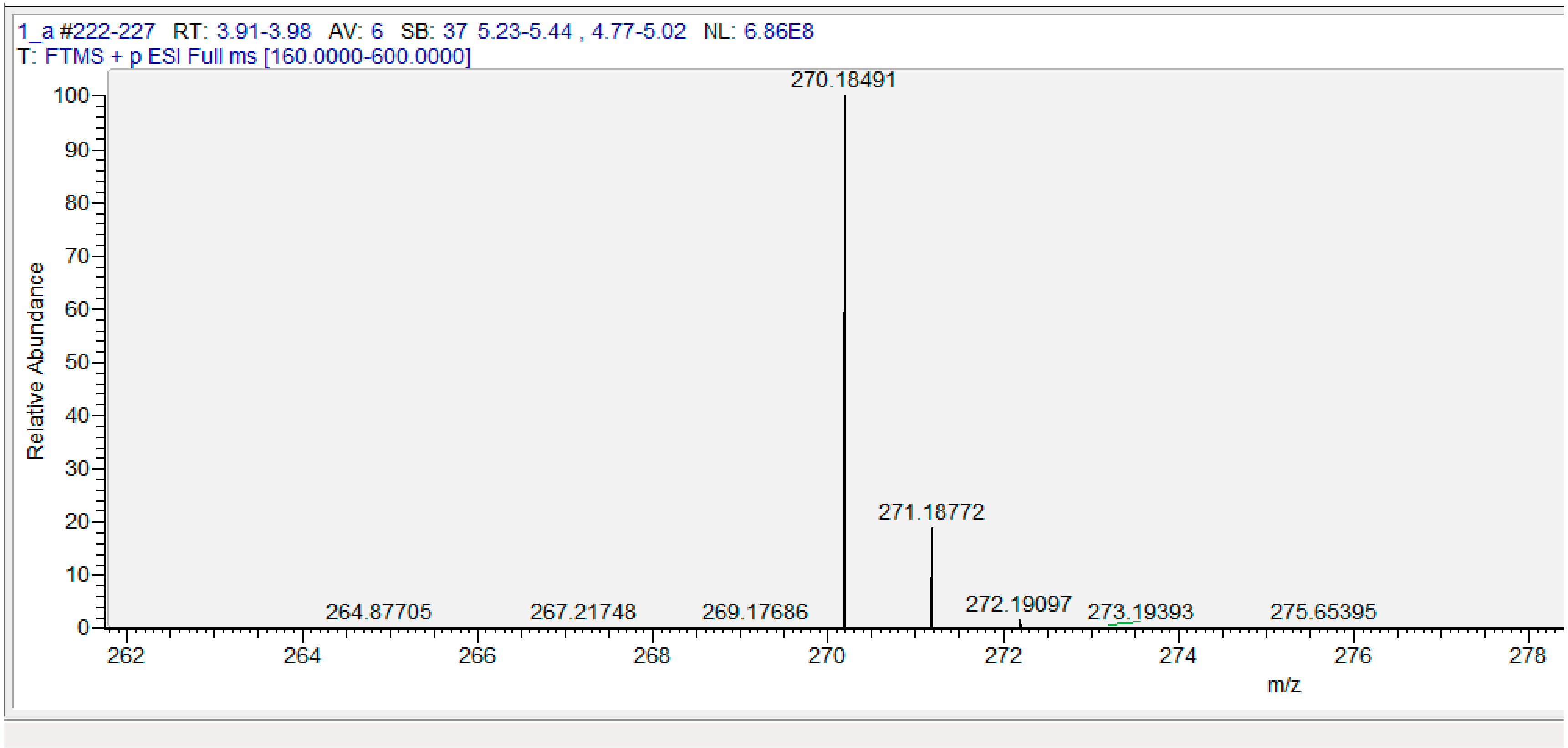

Figure S27. Mass-spectrum of compound **1a**

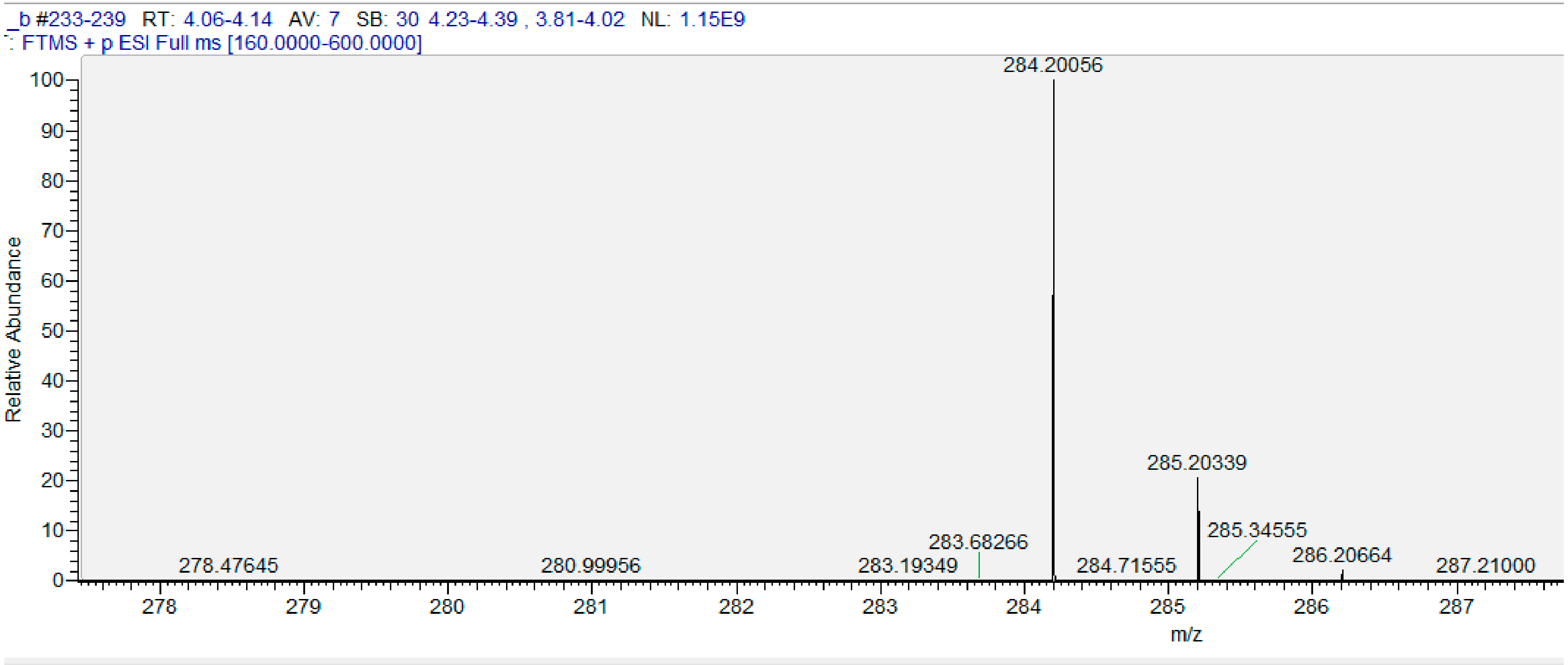

Figure S28. Mass-spectrum of compound **1b**

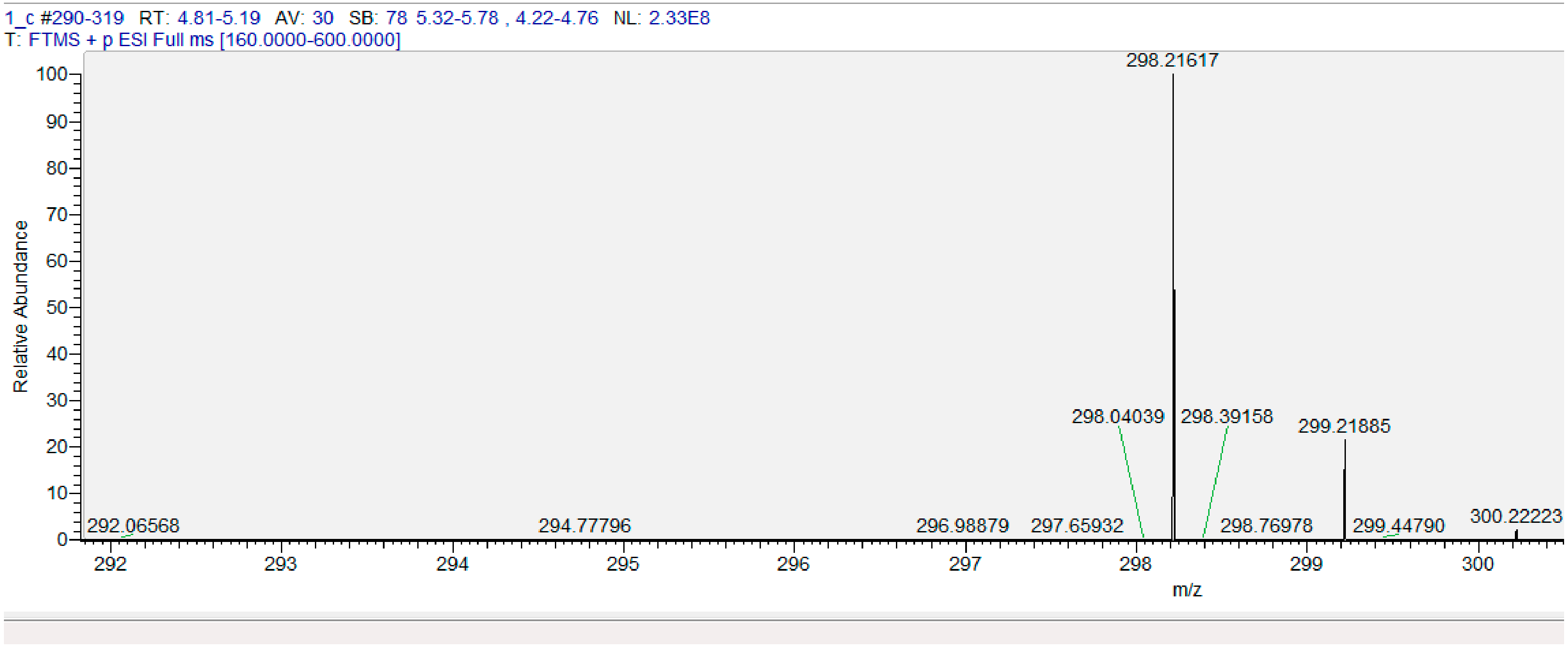

Figure S29. Mass-spectrum of compound **1c**

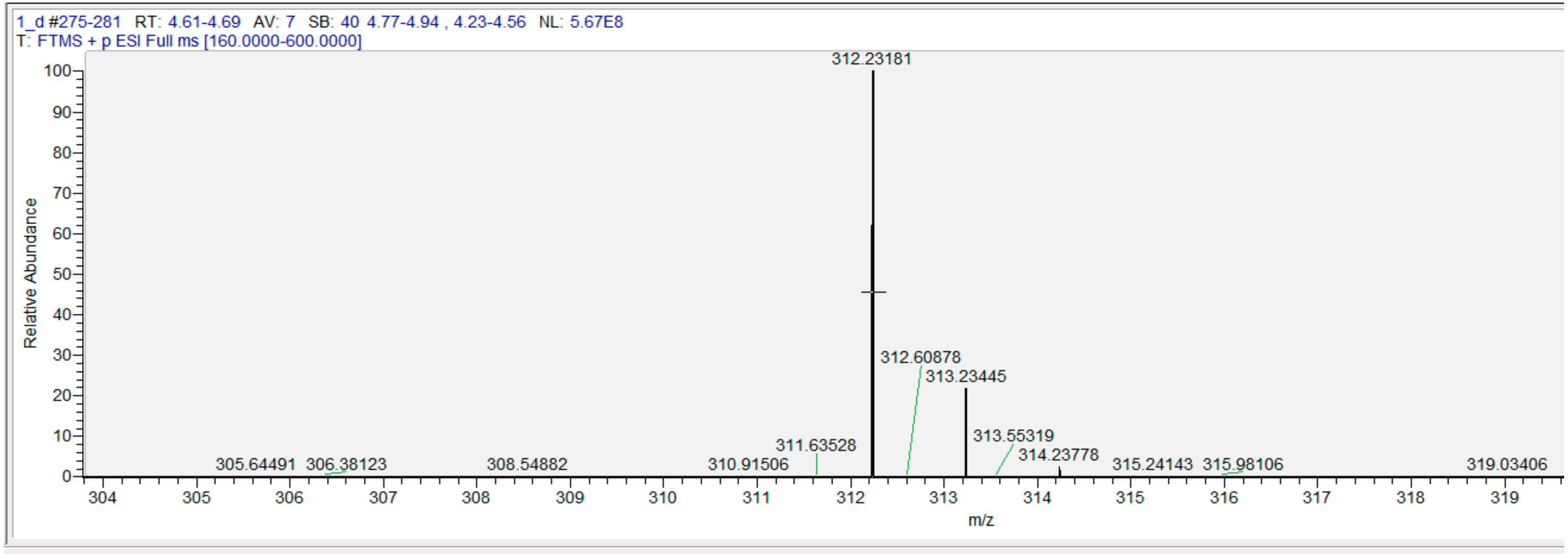

Figure S30. Mass-spectrum of compound **1d**

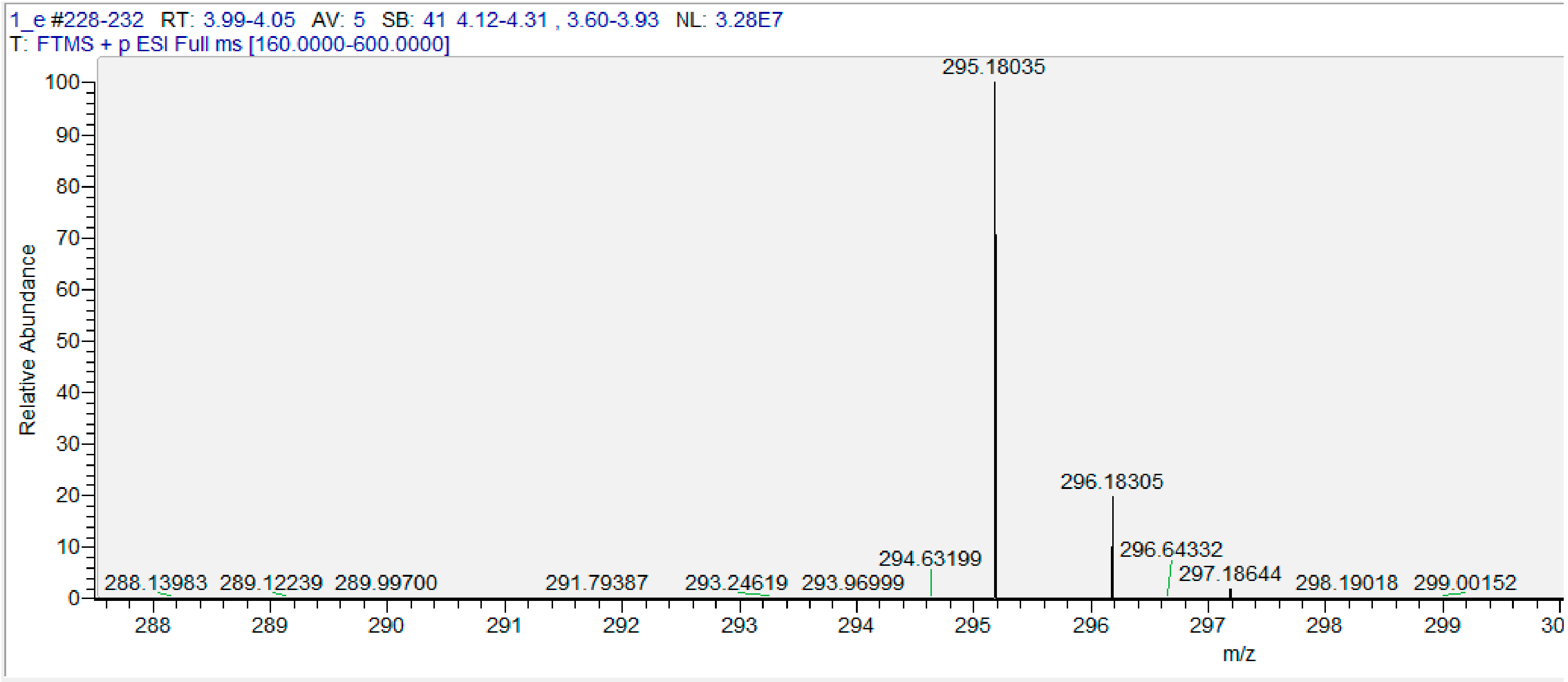

Figure S31. Mass-spectrum of compound **1e**

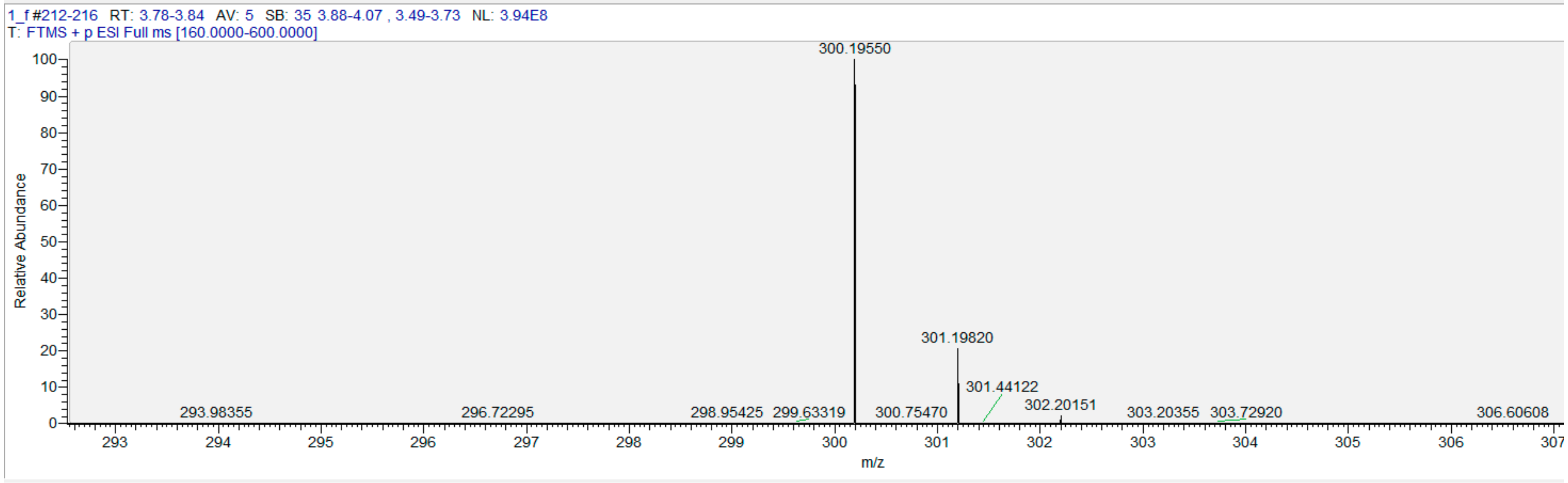

Figure S32. Mass-spectrum of compound **1f**

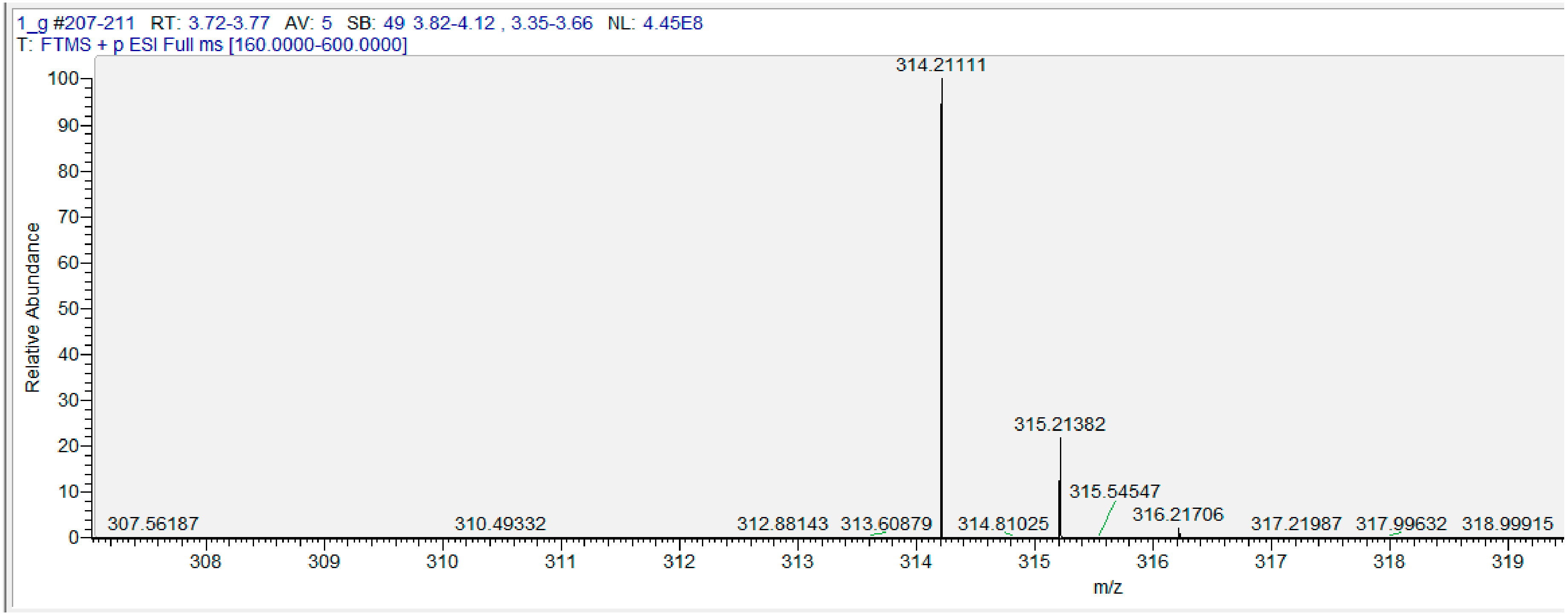

Figure S33. Mass-spectrum of compound **1g**

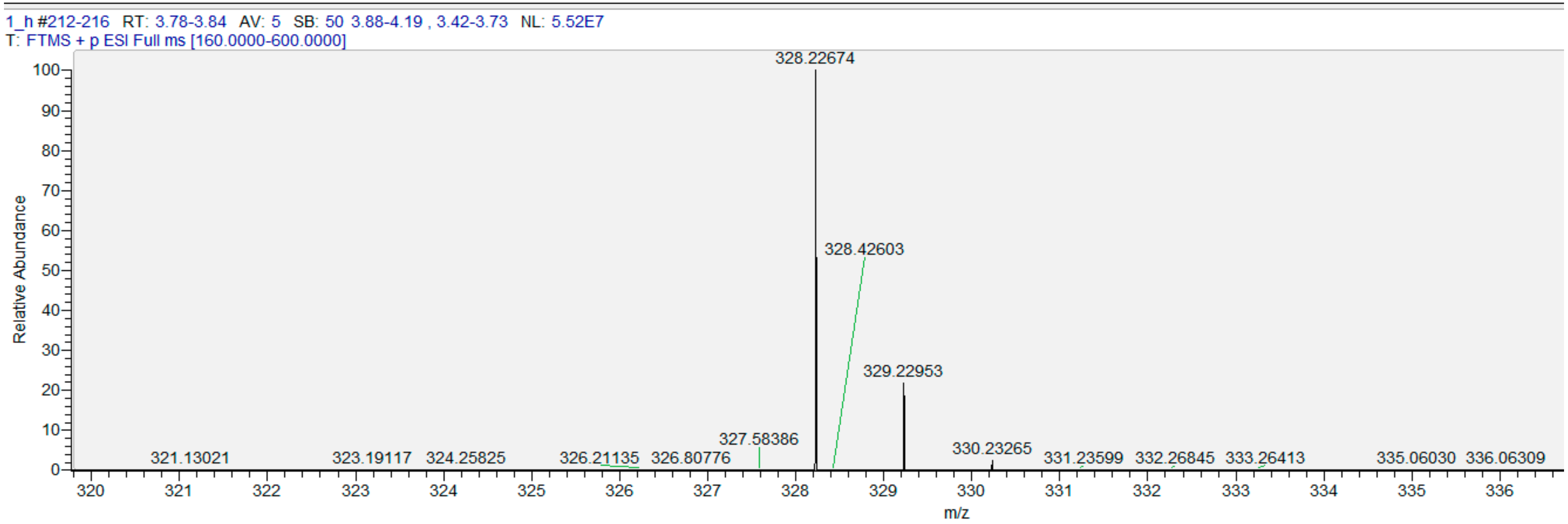

Figure S34. Mass-spectrum of compound **1h**

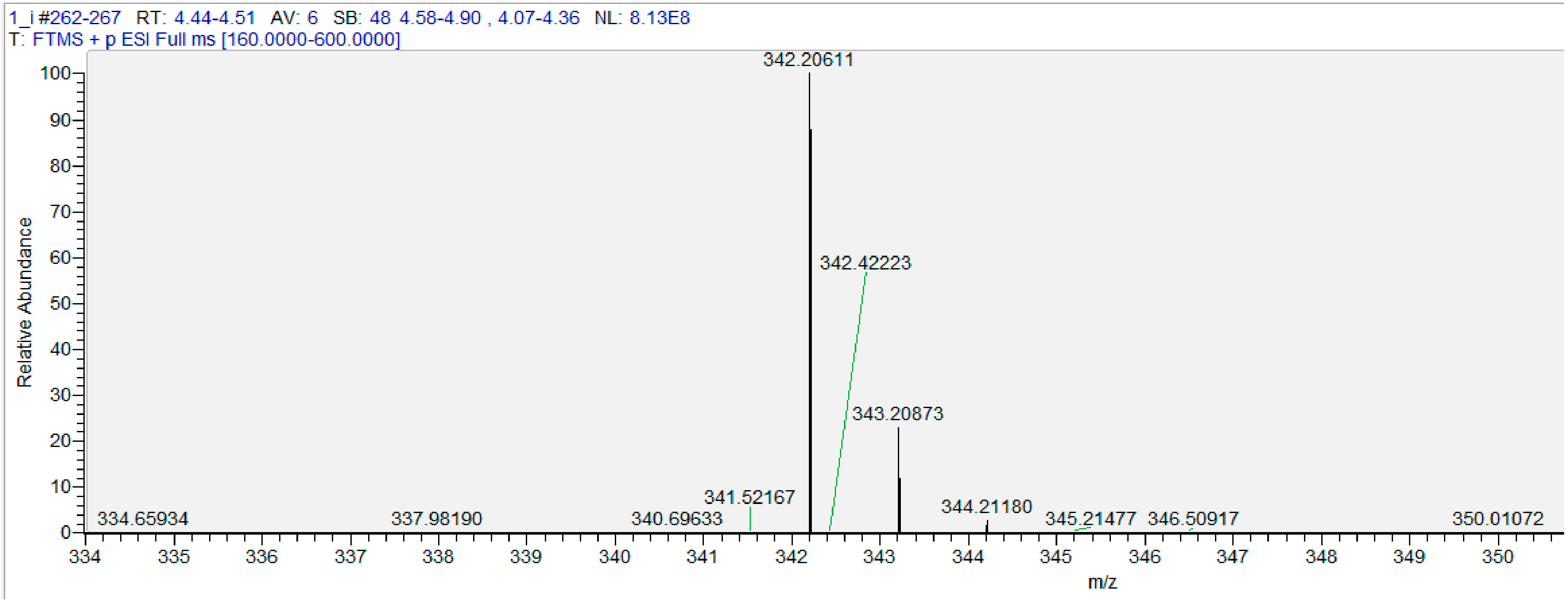

Figure S35. Mass-spectrum of compound **1i**

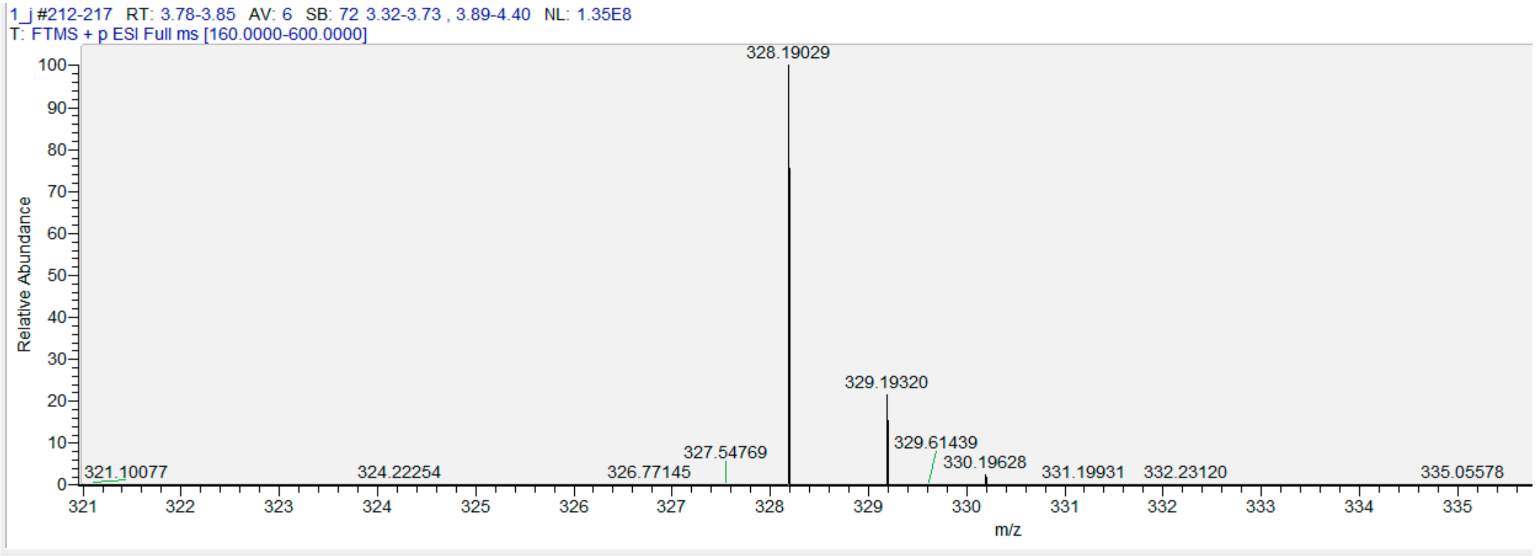

Figure S36. Mass-spectrum of compound **1j**

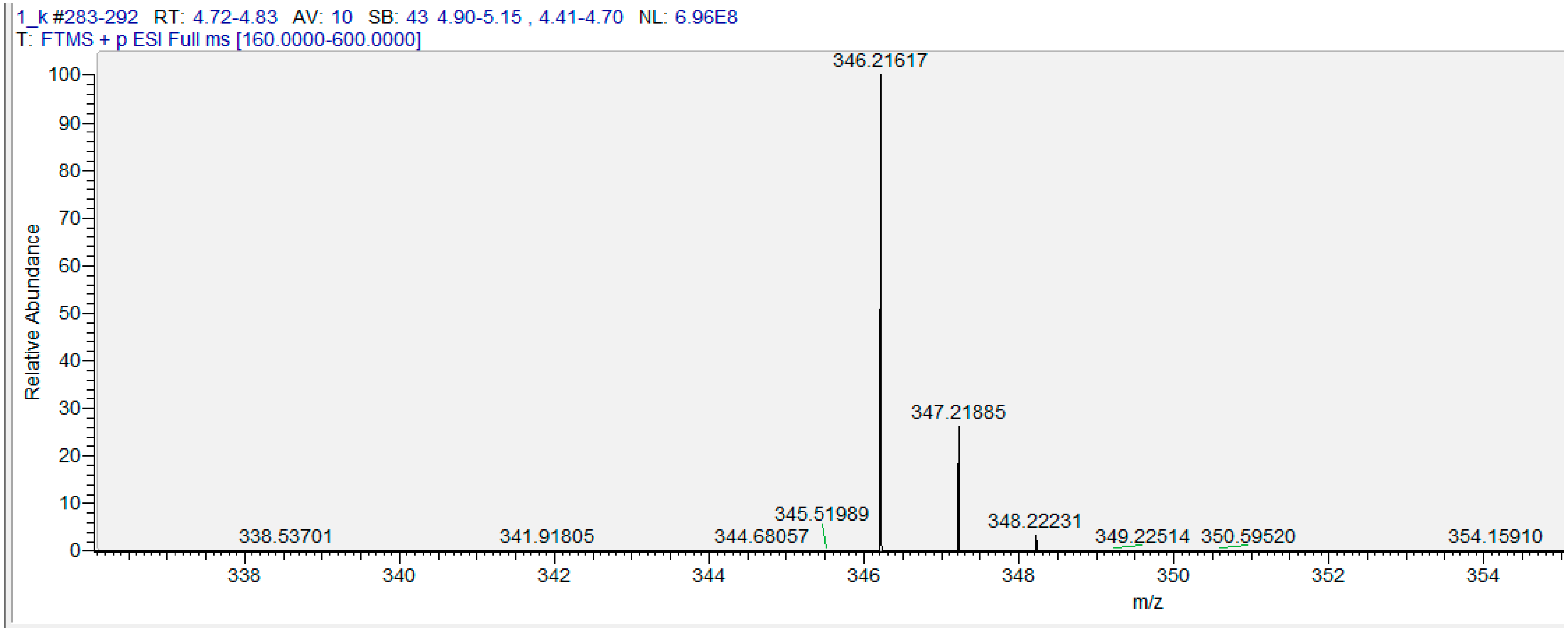

Figure S37. Mass-spectrum of compound **1k**

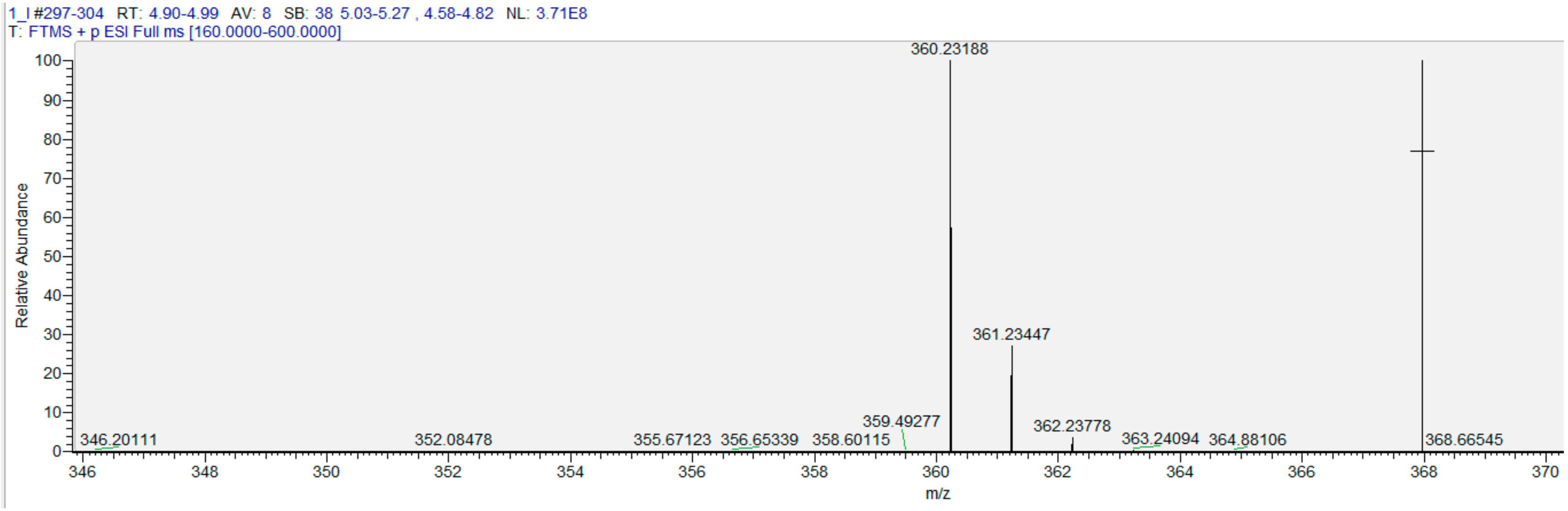

Figure S38. Mass-spectrum of compound **1l**

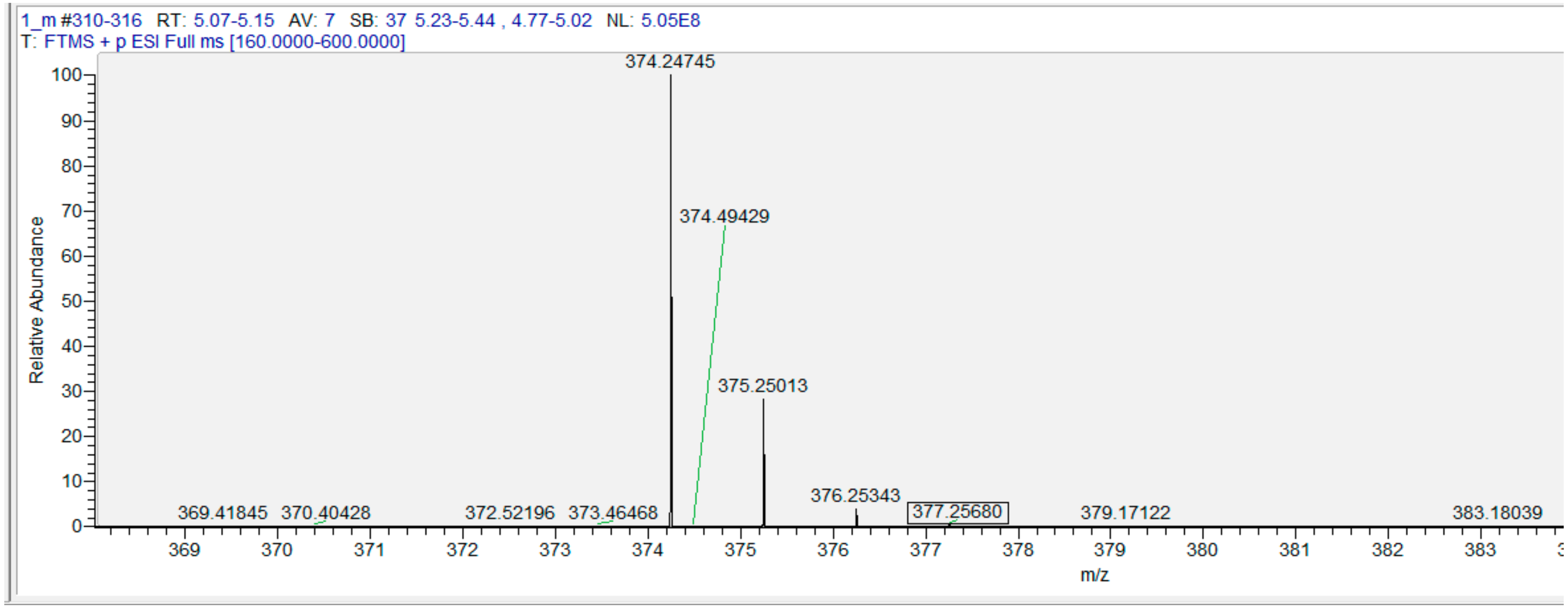

Figure S39. Mass-spectrum of compound **1m**

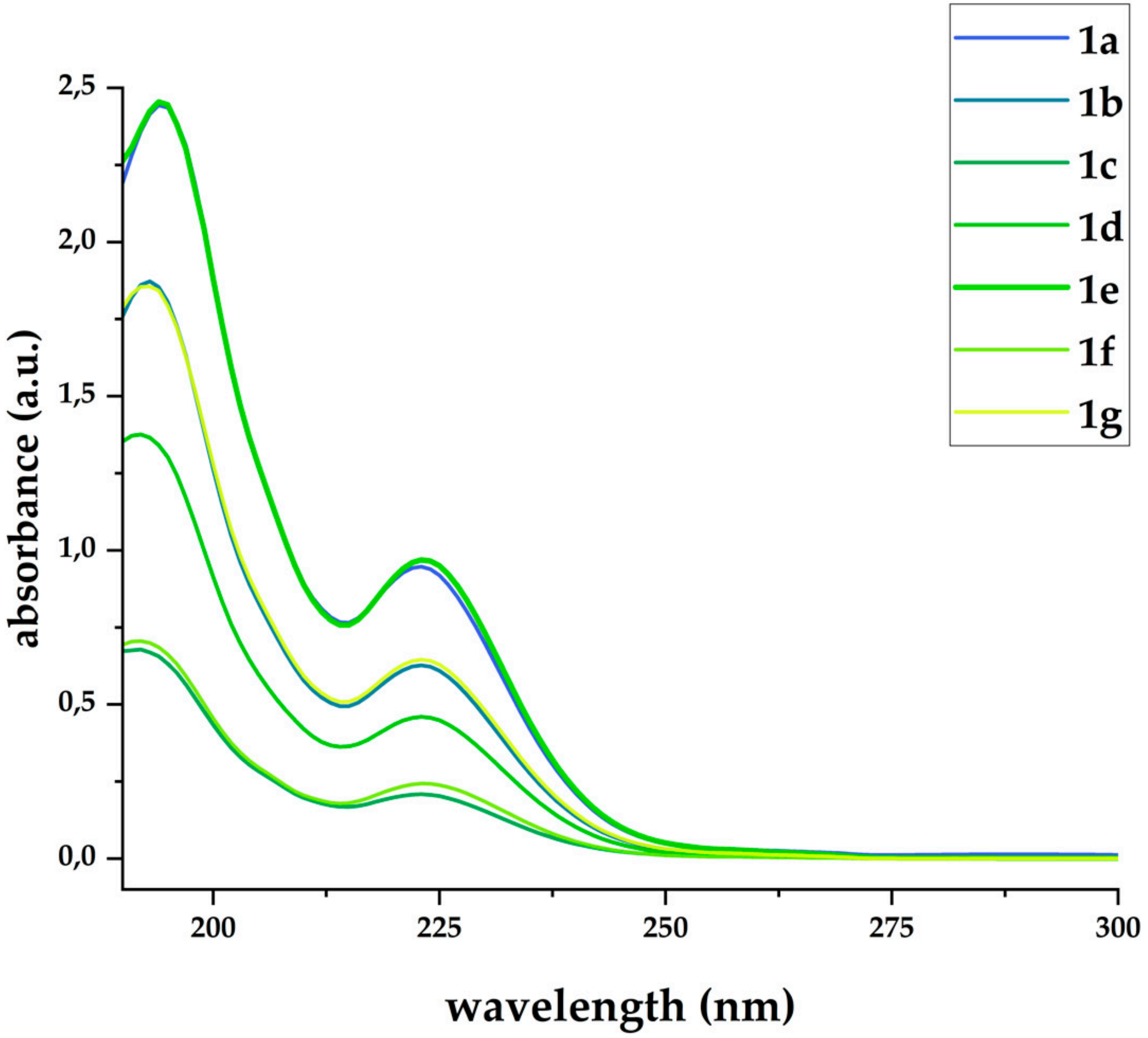

Figure S40. UV spectra of compounds **1a-1g**.

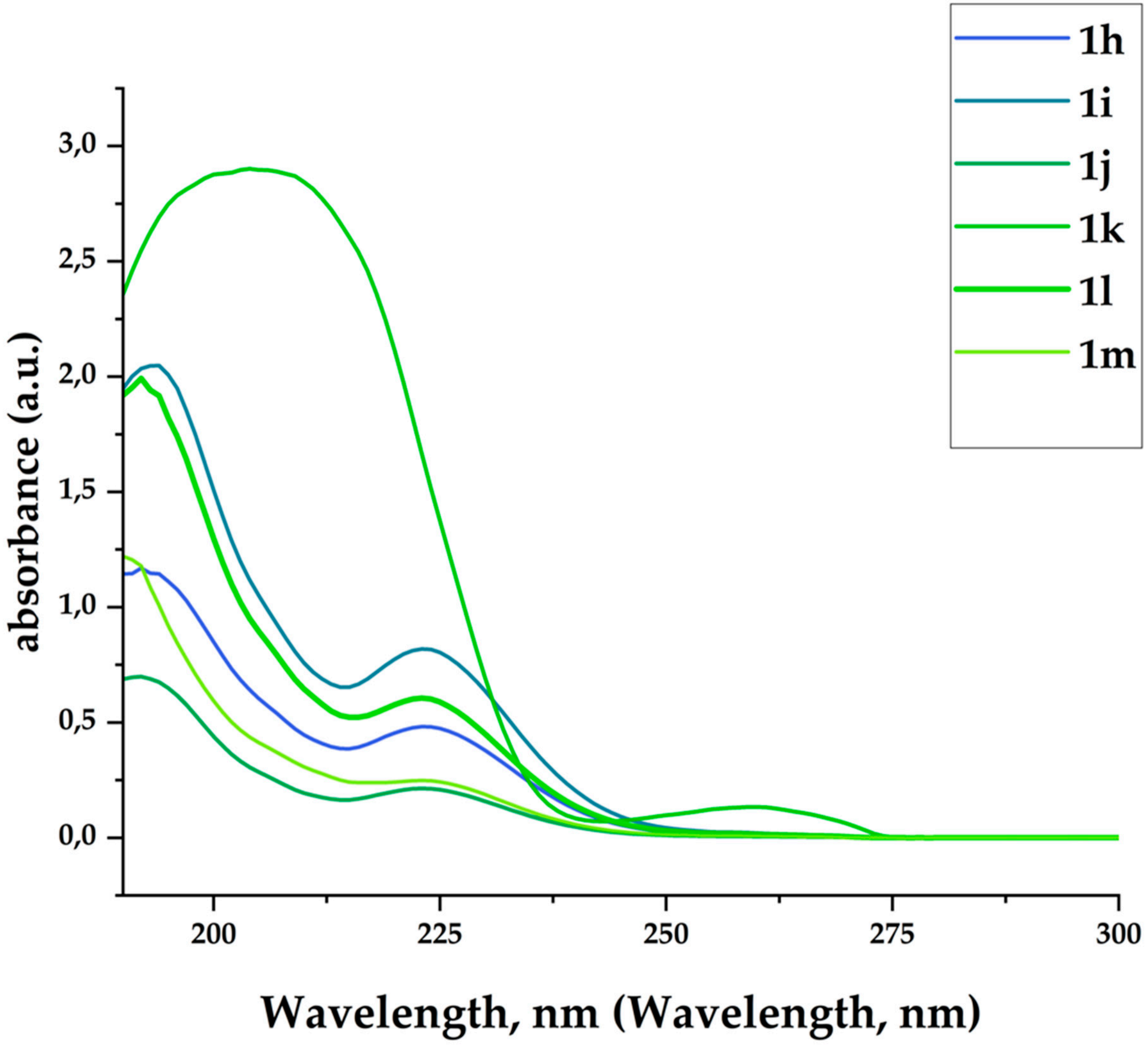

Figure S41. UV spectra of compounds **1h-1m**.

Table S1. Diphenhydramine hydrochloride and synthesized ionic derivatives of diphenhydramine.

Code	R, the substituent on the tertiary nitrogen	Structure	Chemical name
1·HCl		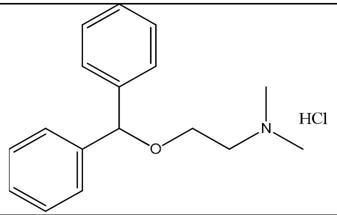	2-(benzhydryloxy)-N,N-dimethylethanamine hydrochloride (Diphenhydramine hydrochloride)
**1a**	$-CH_3$	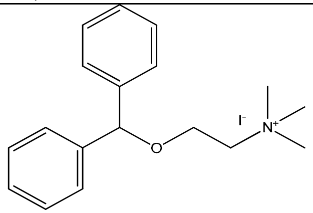	2-(benzhydryloxy)-*N*,*N*,N-trimethylethanammonium iodide
**1b**	$-C_2H_5$	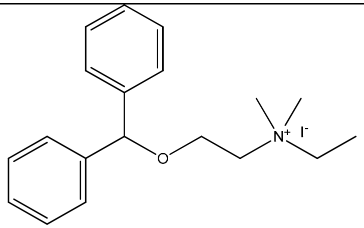	2-(benzhydryloxy)-*N*-ethyl-N,N-dimethylethan ammonium iodide
**1c**	-n-C_3H_7	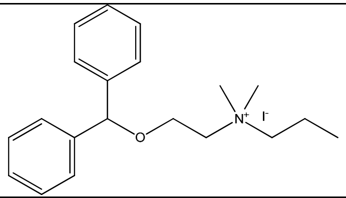	*N*-(2-(benzhydryloxy)ethyl)-*N*,*N*-dimethylpropan-1-ammonium iodide
**1d**	-n-C_4H_9	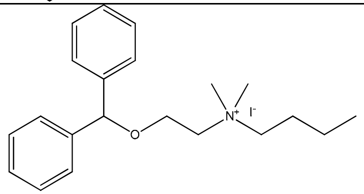	*N*-(2-(benzhydryloxy)ethyl)-*N*,*N*-dimethylbutan-1-ammonium iodide
**1e**	$-CH_2CN$	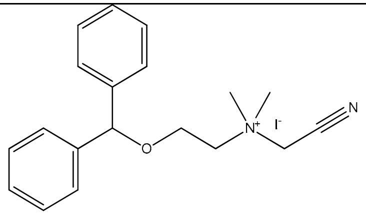	2-(benzhydryloxy)-*N*-(cyanomethyl)-*N*,*N*-dimethylethanammonium iodide

**1f**	$-CH_2CH_2OH$	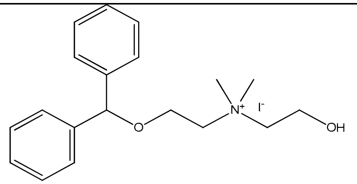	2-(benzhydryloxy)-N-(2-hydroxyethyl)-N,N-dimethylethanammonium iodide
**1g**	$-(CH_2)_3OH$	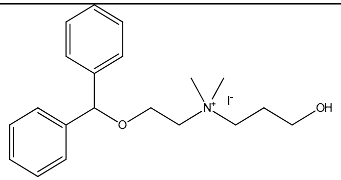	*N*-(2-(benzhydryloxy)ethyl)-3-hydroxy-*N,N*-dimethylpropan-1-ammonium iodide
**1h**	$-(CH_2)_4OH$	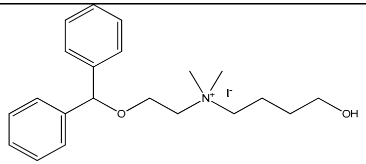	*N*-(2-(benzhydryloxy)ethyl)-4-hydroxy-*N,N*-dimethylbutan-1-ammonium iodide
**1i**	$-CH_2COOEt$	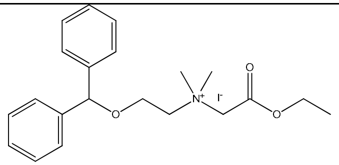	*N*-(2-(benzhydryloxy)ethyl)-2-ethoxy-*N,N*-dimethyl-2-ammonium iodide
**1j**	$-(CH_2)_3COOH$	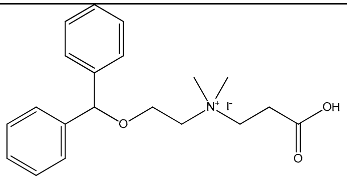	2-(benzhydryloxy)-*N*-(2-carboxyethyl)-*N,N*-dimethylethanammonium iodide
**1k**	$-CH_2C_6H_5$	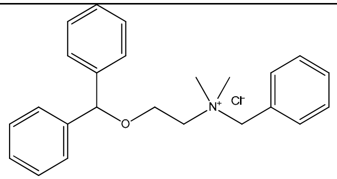	2-(benzhydryloxy)-*N*-benzyl-*N,N*-dimethylethanammonium chloride
**1l**	$-CH_2CH_2C_6H_5$	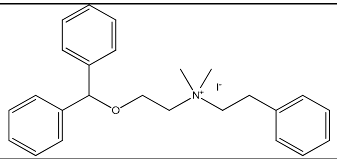	2-(benzhydryloxy)-*N,N*-dimethyl-*N*-phenethylethanammonium iodide
**1m**	$-(CH_2)_3C_6H_5$	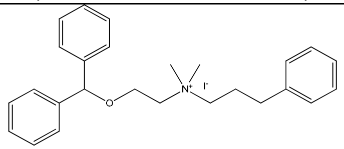	*N*-(2-(benzhydryloxy)ethyl)-*N,N*-dimethyl-3-phenylpropan-1-ammonium iodide

Table S2. PASS prediction of hematopoietic activities for the studied compounds 1*HCl and **1a-1m**

**Activity**	**P_a***	**P_i***	**Compound**
Leukopoiesis stimulant	0.464	0.059	1HCl
Erythropoiesis stimulant	0.319	0.159	1HCl
Leukopoiesis stimulant	0.427	0.077	**1a**
Erythropoiesis stimulant	0.286	0.197	**1a**
Leukopoiesis stimulant	0.377	0.108	**1b**
Leukopoiesis stimulant	0.397	0.094	**1c**
Leukopoiesis stimulant	0.417	0.082	**1d**
Leukopoiesis stimulant	0.278	0.207	**1e**
Leukopoiesis stimulant	0.467	0.058	**1f**
Erythropoiesis stimulant	0.271	0.219	**1f**
Leukopoiesis stimulant	0.408	0.087	**1g**
Leukopoiesis stimulant	0.436	0.072	**1h**
Leukopoiesis stimulant	0.497	0.046	**1i**
Leukopoiesis stimulant	0.460	0.061	**1j**
Erythropoiesis stimulant	0.346	0.131	**1j**
Leukopoiesis stimulant	0.332	0.145	**1k**
Erythropoiesis stimulant	0.251	0.249	**1k**
Leukopoiesis stimulant	0.331	0.146	**1l**
Leukopoiesis stimulant	0.285	0.198	**1m**

Table S3. Hemogram parameters of peripheral blood

	WBC, ·10^9/L	**NEU ·10^9/L**	**LYM ·10^9/L**	**MON ·10^9/L**	**EO, ·10^9/L**	**BAS, ·10^9/L**	**NEU, %**	**LYM %**	**MON %**	**EO, %**	**BAS, %**	**RBC, ·10^{12}/L**	**HGB, g/L**	**HCT**	**MCV**	**MCH**	**MCHC**	**RDW sd**	**RDW cv**	**PLT, ·10^9/L**	**MPV**
**1a**	5,77±0,00	1,92±0,23	3,75±0,42	0,07±0,01	0,01±0,00	0,03±0,00	33,3±0,11	65,0±1,4	1,3±0,1	0,0±0,0	0,3±0,0	8,88±0,12	158,5±3,3	36,3±0,2	40,85±1,2	17,85±1,3	436,0±12,1	12,9±0,4	18,5±1,3	225,5±28,5	4,5±1,1
**1c**	2,46±0,4	0,9±0,01	1,31±0,2	0,19±0,01	0,04±0,00	0,01±0,0	36,6±4,01	53,4±1,64	7,8±0,20	1,8±0,2	0,4±0,01	8,46±0,31	149±12,4	35,7±1,2	42,1±1,4	17,6±1,6	417±13,4	15,8±1,2	16,8±1,4	330±13,5	5,2±1,4
**1d**	7,24±1,03	2,32±0,40	4,1±0,03	0,68±0,01	0,1±0,0	0,02±0,0	32,1±3,21	56,7±4,64	9,5±0,30	1,4±0,02	0,3±0,0	8,21±1,4	147±12,51	35,6±4,3	43,3±2,21	17,9±1,12	414±13,4	17,9±1,7	16,9±1,7	155,0±16,9	8,1±1,7
**1e**	5,45±0,51	1,74±0,31	2,89±0,01	0,65±0,0	0,11±0,0	0,02±0,0	32,0±4,02	53,3±3,04	11,8±0,71	2,25±0,21	0,6±0,0	7,28±1,24	135±14,81	31,15±3,24	42,7±8,41	18,55±1,64	434,0±16,2	14,4±1,14	16,85±1,02	207,0±16,0	6,85±0,4
**1i**	5,43±0,31	1,26±0,04	3,88±0,35	0,16±0,01	0,10±0,0	0,01±0,0	23,2±2,05	71,4±2,4	3,05±0,45	2,0±0,0	0,2±0,1	8,20±0,51	149,5±14,5	35,05±2,75	42,7±0,7	18,15±0,65	424,5±7,5	16,9±2,0	18,4±0,2	291,5±74,5	7,05±2,2
**1j**	5,64±0,44	2,04±0,04	3,33±0,49	0,06±0,04	0,2±0,08	0,01±0,0	36,2±3,15	59,0±3,6	1,0±0,6	3,6±1,6	0,2±0,5	7,93±0,09	143,0±2,0	32,6±0,7	41,1±1,4	18,0±0,5	438±12,4	14,3±0,85	19,2±0,5	243±64,5	3,4±0,4
**1m**	9,65±2,85	3,55±1,08	5,79±1,72	0,14±0,03	0,16±0,02	0,08±0,0	36,7±0,35	59,9±0,05	1,52±0,15	1,72±0,25	0,8±0,21	8,07±0,09	148,5±4,5	34,25±0,95	42,45±0,65	18,4±0,04	433,5±15,1	18,25±0,71	20,5±0,24	204,2±3,54	5,55±1,8
Control	7,28±1,26	2,17±0,64	4,57±0,19	0,31±0,0	0,2±0,0	0,03±0,0	29,8±0,65	62,8±1,75	4,2±0,72	2,8±0,0	0,4±0,0	7,42±1,12	139,56±12,17	30,2±2,34	40,8±1,02	18,7±1,03	459,0±22,5	10,1±0,0	17,0±1,05	340,2±26,1	4,1±0,0
Placebo	3,88±0,92	1,72±0,18	1,57±0,13	0,47±0,24	0,07±0,0	0,03±0,0	44,4±1,61	40,6±1,44	12,2±0,92	2,0±0,0	0,8±0,0	4,09±1,64	71,0±6,04	11,0±0,31	26,9±1,62	17,4±0,02	647,0±28,8	11,5±0,05	31,2±0,31	381,0±19,6	3,5±0,0
Intact	10,74±1,11	0,99±0,79	8,84±1,51	0,29±0,0	0,38±0,0	0,2±0,0	9,3±1,05	82,4±3,16	2,7±0,07	3,5±0,85	2,0±0,0	7,09±1,17	158,5±16,54	36,95±3,21	43,5±2,31	19,45±1,65	446,5±16,5	19,8±4,65	20,95±2,05	561,2±12,2	3,9±0,3
